# Non-Endemic Leishmaniases Reported Globally in Humans between 2000 and 2021—A Comprehensive Review

**DOI:** 10.3390/pathogens11080921

**Published:** 2022-08-16

**Authors:** Rafael Rocha, André Pereira, Carla Maia

**Affiliations:** 1 Global Health and Tropical Medicine (GHTM), Instituto de Higiene e Medicina Tropical (IHMT), Universidade NOVA de Lisboa, 1349-008 Lisbon, Portugal; 2 Faculdade de Medicina Veterinária, Universidade Lusófona, 1749-024 Lisbon, Portugal; 3 Escola Superior de Saúde, Proteção e Bem Estar Animal, Instituto Politécnico da Lusofonia, 1749-024 Lisbon, Portugal

**Keywords:** leishmaniasis, imported, travel, migrants, refugees, humans, One Health

## Abstract

Leishmaniases are human and animal parasitic diseases transmitted by phlebotomine sand flies. Globalization is an important driver of the burden and in the current dynamics of these diseases. A systematic review of articles published between 2000 and 2021 was conducted using the PubMed search engine to identify the epidemiology and clinical management of imported human leishmaniases as a fundamental step to better manage individual cases and traveler and migrant health from a global perspective. A total of 275 articles were selected, representing 10,341 human imported cases. Identified drivers of changing patterns in epidemiology include conflict and war, as well as host factors, such as immunosuppression, natural and iatrogenic. *Leishmania* species diversity associated with different clinical presentations implies diagnostic and treatment strategies often complex to select and apply, especially in non-endemic settings. Thus, diagnostic and management algorithms for medical clinical decision support are proposed. Increased surveillance of non-endemic cases, whether in vulnerable populations such as refugees/migrants and immunocompromised individuals or travelers, could improve individual health and mitigate the public health risk of introducing *Leishmania* species into new areas.

## 1. Introduction

Leishmaniases are a group of diseases caused by protozoa belonging to the genus *Leishmania*. The parasites are transmitted by phlebotomine sand flies, and the disease is zoonotic in most settings. Leishmaniases are worldwide distributed and can be separated geographically into Old World (OW) and New World (NW) diseases, with different species occurring in different areas [1]. Over 20 species have been recognized as human pathogens and clinical manifestations of leishmaniases vary largely but are often divided into two clinically distinct forms: visceral leishmaniasis (VL) and cutaneous leishmaniasis (CL). VL is caused by parasites of the *Leishmania donovani* complex (*L. donovani* in the Old World and *L. infantum* in both the Old and New Worlds) being responsible for causing a severe disease which is lethal when untreated. While *L. donovani* transmission is anthroponotic, the life cycle of *L. infantum* (synonym *L. chagasi* in NW) is mostly zoonotic, with domestic dogs as the main reservoirs of human infection. CL, caused by several species of *Leishmania* and responsible for considerable morbidity in endemic foci, ranges from a benign form with spontaneous resolution, to a disfiguring skin condition involving mucosal tissues [2]. According to [3], 98 countries and territories are endemic to leishmaniasis, with more than 12 million infected people, and with an estimated annual incidence of 50,000 to 90,000 VL cases and 600,000 to 1 million CL cases [4]. 

Reporting policies and practices in endemic countries are inconsistent, with a lack of systematic reporting of all human clinical forms of leishmaniases, leading to an underestimation of the local and global burden of leishmaniasis [5]. On the other hand, the available information shows an increase in the number of imported cases in endemic and non-endemic countries [6,7,8,9,10,11,12], which can be explained by a combination of factors, such as increased human traveling, migration, or population displacement from or to endemic areas and an increase in the number of susceptible populations due to immunosuppressive factors, co-morbidities, and aging. Altogether, increased human mobility and globalization have expanded the at-risk population for leishmaniasis and, simultaneously, pose a risk of geographic expansion of *Leishmania* species. 

The present study aimed to summarize and analyze the epidemiology, clinical presentation, diagnosis, and management of non-endemic human leishmaniasis, through a comprehensive review of the literature in the last 22 years (2000–2021) to raise awareness of the medical community regarding the challenges associated with the diagnosis and management of this parasitic disease.

## 2. Search Strategy, Eligibility, and Review

A comprehensive literature search was performed on 12 November 2021 by sourcing National Library of Medicine (NLM) resources through PubMed (https://pubmed.ncbi.nlm.nih.gov/; accessed on 12 November 2021) using the following Boolean string: (“leish*”[All Fields] AND (“travel”[MeSH Terms] OR “travel”[All Fields] OR “trip”[All Fields] OR “trips”[All Fields] OR “traveling”[All Fields] OR “travelling”[All Fields] OR “travels”[All Fields] OR “traveled”[All Fields] OR “traveler”[All Fields] OR “traveler s”[All Fields] OR “travelers”[All Fields] OR “travelled”[All Fields] OR “traveller”[All Fields] OR “traveller s”[All Fields] OR “travellers”[All Fields] OR (“migrate”[All Fields] OR “migrated”[All Fields] OR “migrates”[All Fields] OR “migrating”[All Fields] OR “migration”[All Fields] OR “migrational”[All Fields] OR “migrant”[All Fields] OR “migrants”[All Fields] OR “migrations”[All Fields] OR “migrator”[All Fields] OR “migrators”[All Fields]) OR (“import”[All Fields] OR “importation”[All Fields] OR “importations”[All Fields] OR “imported”[All Fields] OR “importer”[All Fields] OR “importers”[All Fields] OR “importing”[All Fields] OR “imports”[All Fields]) OR (“refugee s”[All Fields] OR “refugees”[MeSH Terms] OR “refugees”[All Fields] OR “refugee”[All Fields])) AND (“human s”[All Fields] OR “humans”[MeSH Terms] OR “humans”[All Fields] OR “human”[All Fields]) OR “child*”[All Fields] OR “man”[All Fields] OR “woman”[All Fields] OR “patient”[All Fields] OR “patients”[All Fields]).

**Figure 1:**
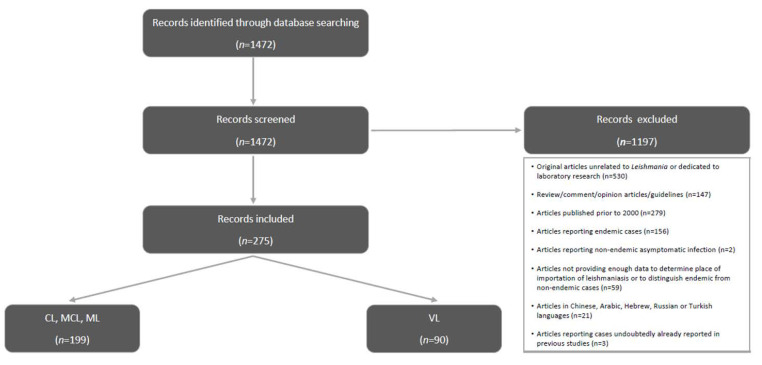
A flow diagram of the study search and selection process.

Search results were saved as a comma-separated value (CSV) file and imported into Microsoft Excel^®^. Study eligibility was manually assessed by two independent researchers in a blind manner. All records were screened according to the title, and abstract, if available. Only studies published between 2000 and 2021 were included, even if the cases reported were diagnosed in previous years. Only original research articles reporting humans with non-endemic *Leishmania* infection (i.e., reportedly infected by *Leishmania* parasites in a country different than the one they were living in at the moment of diagnosis) were retained, including those published in some languages other than English (Figure 1).

The presence of repeated cases in different articles was assessed—either confirmed, when explicitly mentioned in the text, or suspected, based on the authors, place of infection, year, and place of diagnosis (including hospital or center). Articles, where all or most cases reported, had (certainly or likely) been previously described in the literature were mostly discarded (except if they contained clinical or epidemiological details not published in previous works). This was the case for two GeoSentinel articles [13,14]. Articles, where some cases in a series had (certainly or likely) been previously described, were retained, but some cases were discarded, either entirely or in part of the information. This verification process of repeated cases was performed manually and for all the selected articles.

Some records had missing data, and the denominators mentioned in the text and tables count only those where data was available. Articles, where the place of infection included a list of several countries, were counted for the region of infection, but not for the country of infection. The same principle was applied to the place of diagnosis. Regions of infection/diagnosis were defined based on the World Bank Group proposed regions. New World cases grouped all cases infected in the American continent; Old World grouped all the remaining cases. Activity (travel, migration, refugee) was classified based on original articles’ information and considering travel as a broader category including military and missionary service, tourism, visiting friends and relatives, work stays and exchange student programs.

Only laboratory-confirmed leishmaniasis cases were included in this review. Methods and samples for diagnosis were included only when specified for each individual in the article. Besides counting the number of individuals in which each test was performed, the result of the test for each individual was registered: positive (suggestive or confirmatory of current *Leishmania* infection) or negative. Species/complex identification was only considered when the articles mentioned laboratory confirmation, even though the exact technique may not be specified. Even though some articles mentioned identification to the species level, for result analysis and discussion purposes, cases of species of the same complex are presented together, following the classification proposed by [1]:

- *Leishmania (Leishmania)* subgenus: *L. mexicana* complex (*L. mexicana*, *L. amazonensis*, *L. venezuelensis*); *L. donovani* complex (*L. donovani*, *L. infantum* (=*L. chagasi*); *L. tropica* complex (*L. tropica*, *L. killicki*, *L. aethiopica*); *L. major*;
- *Leishmania (Mundinia)* subgenus: *L. martiniquensis*; *L.* “siamensis”;
- *Leishmania* (*Viannia*) subgenus: *L. braziliensis* complex (*L. braziliensis*, *L. peruviana*); *L. guyanensis* complex (*L. guyanensis*, *L. panamensis*); *L. lainsoni*; *L. naiffi*.

Clinical signs, symptoms, and laboratory findings were extracted, whenever available, using the terms contained in the original articles. Patients with splenomegaly, hepatomegaly or both were grouped under the same category. Patients with unspecific/constitutional symptoms were also grouped. For CL, lesion type was classified into four categories, following classical dermatological lesion classification nomenclature: ulcerated lesions; papular/nodular lesions; macular lesions, plaques and crusts; other (whenever this term was used in the original articles). Classification of mucosal (ML) and mucocutaneous (MCL) leishmaniasis cases was performed by the authors, following the definition proposed in the “Manual on case management and surveillance of the leishmaniases in the WHO European Region” [5], whenever clinical information provided allowed, and regardless of the article’s original classification. In articles with insufficient clinical information, ML/MCL classification was assumed according to the article [11,15]. Immunosuppression status included diabetes mellitus, malignancy, transplant, HIV infection and pharmacological immunosuppression (not-transplant related). Post-Kala-azar Dermal Leishmaniasis (PKDL), disseminated and diffuse CL were considered specifically when this term was used in the original article.

## 3. Results and Discussion

### 3.1. Human Leishmania Infection and Leishmaniasis

#### 3.1.1. Visceral Leishmaniasis

**Figure 2:**
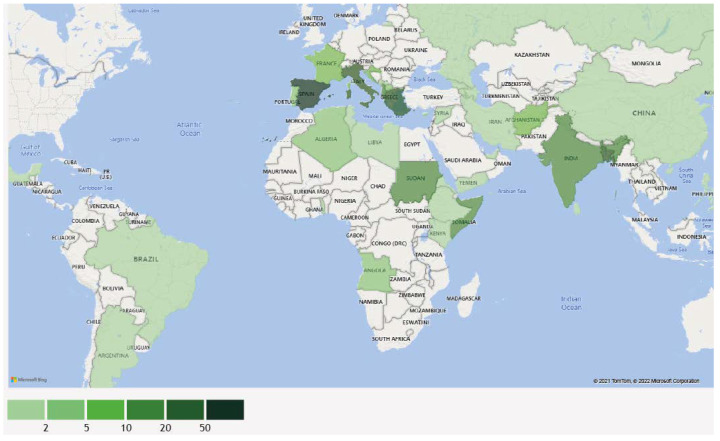
The number of cases of visceral leishmaniasis diagnosed, by country of travel or migration.

Five hundred forty cases of non-endemic human VL were described within the selected period and criteria. In most cases for which the place of infection was described, it likely occurred in Europe (60.8%), followed by South Asia and East Asia (14.8%) and Sub-Saharan Africa (9.8%) (Table 1). Forty-one countries or territories were identified as likely places of infection for 302 patients (Figure 2; Supplementary Table S1).

Eighty-five percent of patients traveled to these regions, whereas 15% were migrants or refugees from these areas. Among travelers, most people were tourists, traveling for work or visiting friends and relatives; only three VL cases were described as military personnel: two deployed in Afghanistan [21] and one in Bangladesh [19]. Refugees were from Somalia (*n* = 17; [16]) and Ethiopia (*n* = 1; [17]), while migrants with VL were from Europe and Central Asia (*n* = 19; [25,36,44,60,208,214,216]), Sub-Saharan Africa (*n* = 4; [20,28,29]), Latin America (*n* = 2; [55,199]) and Middle East (*n* = 2; [10,20]). In one case, a transplacental transmission was described [45]. Patients were diagnosed in 22 countries, mostly in Europe (*n* = 434). Outside the European region, cases were diagnosed in Australia (*n* = 10, [28,36,45,57,64,211,251]), Kenya (*n* = 16, [16]), Kuwait (*n* = 36, [19]), Saudi Arabia (*n* = 35, [259]) and the USA (*n* = 8, [21,43,49,51,55,60,215]). The predominance of cases from the Mediterranean region likely reflects more intensive travel to southern Europe, especially in people from countries where most cases were diagnosed such as France (*n* = 107, [6,18,61,68]), the UK (*n* = 80, [22,38,50,52,58,201,204,207,210,214,219,252,253,261]) and Germany (*n* = 68, [8,20,25,27,33,35,37,40,44,46,47,53,56,63,198,203,250]). Additional factors for this finding could be stronger monitoring and reporting systems in countries where cases of endemic leishmaniasis also occur [283] and the presence of networks such as LeishMan [284].

Around 72% (164/227) of patients were male and the median age was 34.5 years old (range 4 months–86 years), with 29% (*n* = 42) of patients less than 10 years old and 18% of patients over 65 (*n* = 26) (Table 1; Supplementary Figure S1). 

Clinical presentation of non-endemic cases showed many similarities with the classical description of VL from endemic areas [285,286,287]: fever, isolated or combined hepatomegaly/splenomegaly and constitutional symptoms were the signs/symptoms most commonly described (Table 2). Dermatologic manifestations were reported in nine cases, of which three were described as CL (with typical ulcerated lesions) [40,66,257], while six presented other symptoms such as hyperpigmentation, rash, erythroderma [19,31,35,55,204,252]. Rare and atypical manifestations, with the exception of two patients with isolated lymphadenopathy [37,198] and one with kidney involvement [29], were reported in immunocompromised patients, namely involvement of the eye (*n* = 1, [35]), lung (*n* = 2, [32,34]) gastrointestinal tract (*n* = 6 gastroduodenal, [28,197,261]; *n* = 4 colorectal, [48,204,252,261]; *n* = 1 with both, [35]).

Similarly, laboratory findings of 125 patients translated the natural history of the disease well known in endemic countries [286]: most patients had single or multiple lineage cytopenias, while elevation of liver enzymes and renal failure were less commonly described (Table 2). In 22 patients, laboratory findings were consistent with hemophagocytic lymphohistiocytosis (HLH) [17,26,44,49,50,52,59,68,206,214]. Although the association between leishmaniasis and HLH seems to be a rare finding in the pediatric population of endemic areas [288], cases occurring in infants have been reported in imported cases [44], so in children under 2 years of age traveling to endemic areas, leishmaniasis should be included in the differential diagnosis of secondary HLH.

The diagnostic approach was described for 343 patients (Table 3): microscopy and serology were the techniques most often used. Bone marrow aspiration or biopsy was the most commonly used biological samples for microscopy, polymerase chain reaction (PCR) and culture. Positivity in these samples was higher using PCR (96.6%), followed by microscopy (91.5%) and culture (88.6%). For PCR, blood was also commonly used (30.5%). Other samples where positive parasitological results were occasionally obtained included liver and lymph nodes, the last ones possibly representing part of the path for investigation of alternative diagnoses, such as lymphoma.

**Table 3:** The diagnostic approach of human leishmaniasis.

Description	VL			CL/MCL/ML		
	Frequency Patients Tested	Frequency Tested Positive	References	Frequency Patients Tested	Frequency Tested Positive	References
Microscopy	84.8% (291/343)	91.8% (302/329)		63.4% (1072/1690)	84.3% (904/1072)	
Bone marrow	84.5% (246/291)	91.5% (225/246)	[12,17,19,20,24,26,27,29,31,32,35,38,40,42,43,44,46,47,49,51,53,55,57,58,59,60,61,62,63,64,65,66,68,199,200,201,202,203,205,207,209,210,211,212,213,214,215,216,217,218,219,249,250,251,256,257,258,260,261,262]	NR	NA	NA
Liver	10.3% (30/291)	90.0% (27/30)	[17,21,26,32,36,202,216,257,261]	NR	NA	NA
Spleen	8.6% (25/291)	92.0% (23/25)	[16,17,23,38,206,216,252,257,261]	NR	NA	NA
Lymph node	1.7% (5/291)	100% (5/5)	[35,37,198,261]	0.1% (1/1072)	100% (1/1)	[82]
Blood	1.0% (3/291)	100% (3/3)	[21,24,29]	NR	NA	NA
Skin	1.0% (3/291)	100% (3/3)	[66,257,261]	96.7% (1037/1072)	84.2% (873/1037) ^a^	[7,11,12,20,27,30,38,66,69,70,71,72,73,75,77,78,79,80,81,82,84,85,86,87,88,89,90,91,92,95,96,97,98,99,100,101,103,104,105,106,110,113,115,116,117,119,120,121,122,123,124,126,127,128,129,131,134,137,139,140,141,143,144,145,147,148,149,150,151,152,153,156,157,159,160,162,163,164,165,166,167,168,169,170,171,174,175,176,179,181,182,183,184,185,186,187,191,194,195,196,197,221,222,224,225,226,227,228,229,230,231,234,235,236,237,238,239,240,241,242,243,244,246,247,254,255,263,268,271,275,279,280,281]
Other	5.5% (16/291) ^b^	93.8% (15/16)	[32]	NR	NA	NA
Mucosa ^c^	NR	NA	NA	3.1% (33/1072)	90.9% (30/33)	[11,26,30,38,76,82,107,108,109,111,114,130,135,146,152,158,161,180,190,223,233,245]
Serology	68.8% (236/343)	95.2% (318/334)		6.8% (115/1690) ^d^	69.1% (85/123)	
DAT	45.1% (97/215)	96.9% (94/97)	[16,38,42,52,257,259,261]	6.1% (6/99)	66.7% (4/6)	[103,158]
rK39	44.2% (95/215)	93.7% (89/95)	[38,52,60,204,210,211,213,257,259,261,262,281]	17.2% (17/99)	64.7% (11/17)	[114,119,158,174,190,277]
IFAT	41.4% (89/215)	94.4% (84/89)	[18,19,20,25,29,32,33,40,43,44,47,59,198,202,203,212,216,250,257]	73.7% (73/99)	71.2% (52/73)	[11,71,80,89,101,104,107,119,129,135,138,143,179,226]
ELISA	13.0% (28/215)	92.9% (26/28)	[18,20,33,37,40,41,44,47,68,199,203,250]	7.1% (7/99)	57.1% (4/7)	[70,89,135,144,170,176,179]
WB	1.9% (4/215)	100% (4/4)	[25,68,203,256]	4.0% (4/99)	100% (4/4)	[104,121,135]
PCR	51.6% (177/343)	94.1% (206/219)		84.9% (1435/1690)	97.2% (1398/1438)	
Bone marrow	83.7% (118/141)	96.6% (114/118)	[12,20,21,22,24,26,27,29,32,33,40,42,44,45,47,48,49,50,51,52,57,58,59,61,62,63,64,65,66,68,199,209,210,211,212,214,215,250,253,260]	NR	NA	NA
Blood	30.5% (43/141)	93.0% (40/43)	[18,20,24,25,29,32,41,44,48,60,61,62,66,202,217,250,260]	0.1% (2/1435)	100% (2/2)	[20]
Liver	7.1% (10/141)	90.0% (9/10)	[21,26,32,48,202]	NR	NA	NA
Spleen	4.3% (6/141)	100% (6/6)	[16,23,54,59,66,206]	NR	NA	NA
Other	2.8% (4/141) ^e^	100% (4/4)	[28,29,48,204]	NR	NA	NA
Lymph node	1.4% (2/141)	100% (2/2)	[37,56]	0.1% (2/1435)	100% (2/2)	[82,180]
Mucosa ^c^	NR	NA	NA	4.0% (57/1435)	96.5% (55/57)	[11,30,38,76,77,82,107,109,111,114,129,130,133,135,142,146,158,161,173,179,180,190,223,233,245,273]
Skin	NR	NA	NA	96.0% (1378/1435)	97.2% (1340/1378) ^f^	[7,9,11,12,20,26,28,30,38,45,61,65,66,70,75,76,77,78,79,82,83,87,89,91,92,93,94,95,96,97,98,100,102,104,105,106,107,109,110,111,114,116,117,119,121,122,123,124,126,127,128,129,130,132,133,134,135,136,137,138,139,140,141,142,143,144,145,146,147,149,150,151,153,156,157,158,159,161,162,164,165,166,167,168,170,171,172,173,174,175,176,177,178,179,180,181,182,185,186,187,188,189,190,191,192,193,194,195,196,197,223,225,226,227,229,230,231,233,235,236,238,239,240,241,242,243,244,245,246,247,255,266,268,271,273,275,278]
Culture	17.5% (60/343)	76.2% (48/63)		48.7% (823/1690)	86.8% (714/823)	
Bone marrow	76.1% (35/46)	88.6% (31/35)	[12,18,21,27,29,32,44,51,63,181,199,209,250]	NR	NA	NA
Spleen	19.6% (9/46)	100% (9/9)	[16,206]	NR	NA	NA
Lymph node	4.3% (2/46)	100% (2/2)	[11,198]	0.2% (2/823)	100% (2/2)	[82,180]
Blood	2.2% (1/46)	100% (1/1)	[29]	NR	NA	NA
Liver	2.2% (1/46)	100% (1/1)	[21]	NR	NA	NA
Other	2.2% (1/46) ^g^	100% (1/1)	[32]	NR	NA	NA
Mucosa	NR	NA	NA	1.8% (15/823)	86.7% (13/15)	[12,43,73,94,111,207,222,236,259]
Skin	NR	NA	NA	98.2% (808/823)	86.5% (699/808) ^h^	[7,9,12,28,30,69,70,71,72,73,75,77,80,81,82,85,87,89,90,91,95,97,98,100,104,110,116,117,119,120,121,122,123,127,128,129,131,134,137,145,148,149,150,156,159,163,166,169,175,176,181,183,187,221,226,227,228,234,235,242,247,263,266,268,271,275,278]
Species/complex						
*L. donovani* complex	98.8% (167/169)	NA	[9,10,11,16,20,21,22,24,25,26,27,28,29,32,33,37,40,44,45,47,48,49,51,52,54,57,59,60,61,62,63,64,66,68,198,199,202,206,209,210,211,212,214,256,260,261]	See Figure 3	NA	NA
*L. infantum + L. major*	0.6% (1/169)	NA	NA	NR	NA	NA
*L. subgenus Mundinia*	0.6% (1/169)	NA	NA	NR	NA	NA

^a^ Skin biopsy in 474/581 (81.6%) patients; skin smear/scraping in 195/581 (33.6%); positivity in 419/474 (88.4%) and 145/195 (74.4%) of samples, respectively; unspecified technique (*n* = 491); ^b^ Gastric and/or duodenal (*n* = 9); colorectal (*n* = 5); 1 renal (*n* = 1); 1 bronchial biopsy (*n* = 1); ^c^ Excluding gastrointestinal and lower respiratory tract mucosa; ^d^ Including in 21 patients with MCL/ML; in these, positivity was reported in 82.1% of tests performed (23/28); ^e^ Colorectal (*n* = 2); gastric (*n* = 1); renal biopsy (*n* = 1); ^f^ Skin biopsy in 613/690 (88.8%) patients; skin smear/scraping in 115/690 (16.7%); positivity in 603/613 (98.4%) and 115/115 (100%) of samples, respectively; unspecified technique (*n* = 745); ^g^ Lung biopsy (*n* = 1); ^h^ Skin biopsy in 460/525 (87.6%) patients; skin smear/scraping in 67/525 (12.8%); positivity in 423/460 (92.0%) and 61/67 (91.0%) of samples, respectively; unspecified technique (*n* = 298); Abbreviations: VL, visceral leishmaniasis; CL, cutaneous leishmaniasis; MCL, mucocutaneous leishmaniasis; ML, mucosal leishmaniasis; PCR, polymerase chain reaction; ELISA, enzyme-linked immunosorbent assay; IFAT, immunofluorescence antibody test; DAT, direct agglutination test; rK39, rapid immunochromatographic test; NA, not applicable, NR, not reported.

**Figure 3:**
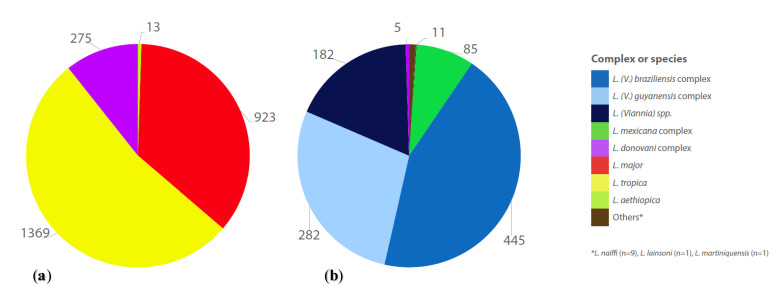
Infecting species/complex in cases of cutaneous, mucocutaneous and mucosal leishmaniasis in: (**a**) Old World; (**b**) New World.

Among serological techniques, indirect fluorescent antibody test (IFAT), direct agglutination test (DAT) and immunochromatographic test (ICT, rK39-based) were employed with similar frequency (40–45%), probably reflecting their more widespread availability in general, and especially in non-endemic countries/areas. The sensitivity and specificity of these tests in immunocompetent patients are considered to be high and therefore good choices for initial diagnosis [5]; in addition, quantitative serological tests (such as IFAT and DAT) are also useful for follow-up, as antibody titers tend to decay after successful treatment [5]; qualitative tests (such as ICT), on the other hand, offer a fast, point-of-care alternative for serological diagnosis.

All cases in which *Leishmania* species/complex was identified (*n* = 169) belonged to the *L. donovani* complex, except for one case caused by the subgenus *Mundinia* [253] (previously identified by the *nomen nudum L.* “siamensis” and later suggested to be *L. martiniquensis* [213]) and a co-infection caused by *L. infantum/L. major* [40]. Visceral disease caused by *L.* (*Mundinia*) species [289] and *L. major* has been rarely reported [290]. *Leishmania major/L. donovani* complex co-infections have been associated with visceral [291] and cutaneous disease [292], including disseminated CL in the context of HIV infection [293,294]. The case identified in this review was a patient chronically medicated with steroids and methotrexate.

Treatment strategies were reported in 256 cases (Table 4): formulations of amphotericin B (AmB) were used in 72% of these cases, pentavalent antimonials in 21%, which is in line with the WHO recommendations for treatment of VL in *L. donovani* complex endemic countries [5]; two cases were initially treated with combined therapy (liposomal amphotericin B-LAmB and miltefosine). Relapse or treatment failure occurred in 14% of the cases where treatment was known (*n* = 35) in 12% of patients initially treated with LAmB and 19% of patients initially treated with antimonials. Retreatments (*n* = 19, [21,32,35,44,47,51,55,58,66,252,260,261] often consisted of additional single or multiple courses of the same treatment strategy (53%, [44,47,51,55,58,66,260,261]) and rarely consisted in combined therapy [35]. Seventy-seven percent of the failures/relapses occurred in immunosuppressed patients [32,35,47,55,58,66,252,257,260,261]. Patients medically immunosuppressed by chemical or biological drugs or presenting immunodeficiencies (such as HIV infection/AIDS) are prone to VL relapses [295,296]; therefore, close monitoring until a sustained immune reconstitution has occurred is advisable [297].

#### 3.1.2. Cutaneous, Mucocutaneous and Mucosal Leishmaniasis

**Figure 4:**
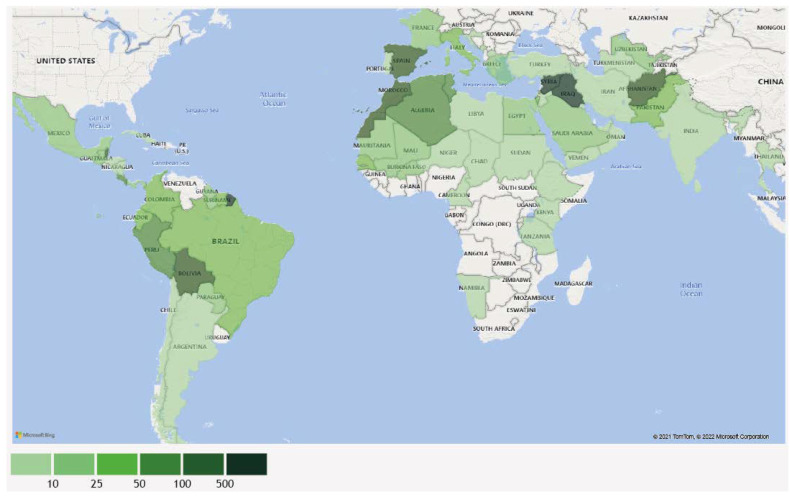
The combined number of cases of cutaneous, mucocutaneous and mucosal leishmaniasis diagnosed, by country of travel or migration.

According to selected criteria, 9771 cases of non-endemic human cutaneous/mucosal disease (Table 1) were described: 9642 CL, 90 MCL and 39 ML. Old World cases represented over 80% of the total. The two regions where most patients were presumably infected were the Middle East and Latin America and the Caribbean. Sixty-seven countries and territories were identified as likely places of infection for 8553 patients (Figure 4; Supplementary Table S2).

Two thousand three hundred thirty-eight (32.7%) patients traveled to these regions, whereas 67.7% were migrants or refugees from these areas. Among travelers, 1027 (43.9%) were described as military personnel: 521 (50.7%) deployed in Iraq [46,292], 183 (17.8%) in Afghanistan [45,112,279] and 167 (16.2%) and 21 (2.0%) in military training in Belize [69,87,96,105,147,163,267,269] and French Guiana [69,136,151], respectively. Most of CL in refugees was reported in individuals from Syria (*n* = 4600, 99.6% of cases, [157,172,181,188,195,246,274]). Migrant individuals (*n* = 186) were infected most often in Latin America (40.6%, [30,101,107,132,139,149,154,193,197,224,276]) and South Asia (27.3%, [20,28,45,68,95,168,187,220]). Overall, migrants represented a small percentage (<15%) of CL, MCL and ML cases from the respective countries, except for: Paraguay (82%, [276]), Colombia (71%, [101]), Nicaragua (33%, [149]), Algeria (32%, [95]), Panama (27%, [139]) and Burkina Faso (26%, [30,95]). Around 58% (3820/6569) of patients were male and the median age was 31 years old (ranging from 9 months to 86 years). The age distribution is represented in Supplementary Figure S2. Reports of the demography of military personnel and refugees likely explain why people aged 20–30 years old were the most affected by cutaneous and mucosal disease [298].

Patients were diagnosed in 35 countries; most of the cases were in the Middle East, Europe, and North America (Table 1). In these three regions, countries with the largest share of diagnosis were Lebanon (57.0%, [192,282]), France (13.4%, [6,61,81,108,121,134,136,193], the USA (6.1%, [72,80,84,88,92,93,110,114,117,120,122,123,127,139,141,142,149,150,159,163,169,175,179,180,197,221,224,229,234,235,238,239,243,244,247,263,266,279]), the Netherlands (5.0%, [12,65,90,106,112,137,162,190,229,240,269]) and the United Kingdom (4.8%, [7,38,82,87,91,94,105,109,130,140,143,147,155,159,165,171,220,222,223,225,267]). Countries outside these three regions where cases were diagnosed were: Australia (*n* = 85, [28,45,85,98,115,196,230]), Venezuela (*n* = 29, [101]), China (*n* = 12, [156,268,271]), Brazil (*n* = 10, [276,281]), Bangladesh (*n* = 6, [103,145,160,174]), Cuba (*n* = 6, [166,237]), Japan (*n* = 4, [190,219,242]), Singapore (*n* = 2, [69]), India (*n* = 1, [183]), Mexico (*n* = 1, [176]), South Korea (*n* = 1, [184]) and Taiwan (*n* = 1, [186]).

The diagnostic approach was described for 1690 patients (Table 3). Although skin biopsy allows for differential diagnosis in suspected cases of CL, the sensitivity of well-performed skin scrapings is similar [299], and the procedure is less invasive, which could explain why these biological samples were used in most cases. The positivity rates reported with PCR (97.2%) were considerably higher than with microscopy (84.2%) or culture (86.5%). Other biological samples occasionally used for diagnosis included mucosal biopsies (*n* = 61, in ML/MCL cases), blood (*n* = 2) and lymph node (*n* = 2, in MCL/ML cases).

**Figure 5:**
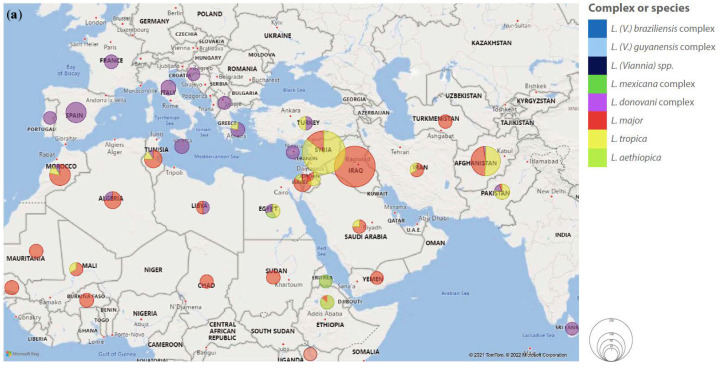

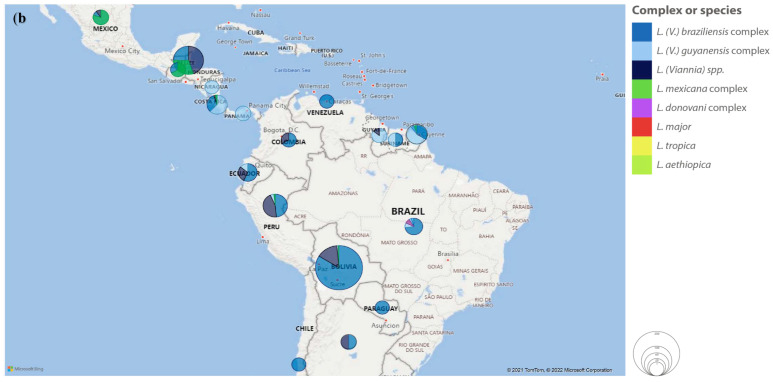
The number and percentage of identifications of species/complex by country in: (**a**) Old World; (**b**) New World.

Even though the use of serological methods for the detection of antibodies against *Leishmania* in CL should be discouraged because of their low sensitivity and variable specificity [5], the performance of serological testing was reported in 115 patients, yielding positive results in 69.1% of the samples tested. IFAT was the technique most commonly used (73.7% of serological tests), followed by ICT (17.2%). Reporting of serological testing was proportionally more common in MCL/ML cases (21/61 versus 94/1629 in CL) and positivity rates were also higher (82.1%); this finding is in line with the WHO recommendation in the European region to include serological methods in the laboratory diagnosis of MCL/ML [300]. Infecting *Leishmania* species/complex was described in 3495 individuals and followed the relative distribution shown in Figure 3, for Old World and New World disease. The distribution of species/complex for each country of infection is represented in Figure 5 and Supplementary Table S3.

Overall, the results demonstrated that human movements have led to an increase in the number of imported CL cases due to non-indigenous *Leishmania* species in both endemic and non-endemic countries. Although the potential risk of introducing these exotic *Leishmania* species into non-endemic areas is low, since for most of them their main reservoirs hosts are absent, the vectorial competence of local sand fly species must be considered, as it may allow for successful adaptation of non-indigenous *Leishmania* species with important epidemiological consequences [301]. The most expressive group of imported cutaneous cases was represented by refugees from war zones in the Middle East diagnosed mostly in neighboring countries (such as Lebanon and Turkey) and reflecting how the ongoing Syrian war has dramatically increased the incidence of CL in these countries [302]. The refugee status of these people in the host countries could be an important factor deterring an early diagnosis of disease. Some countries and centers, such as the ECDC [303], have produced and implemented guidelines and recommendations for the initial healthcare screening of migrants and refugees. Although leishmaniasis is only briefly addressed in these documents, promoting a complete assessment that includes skin checks [304] will probably help shorten onset to treatment intervals in CL. This shortening could be particularly relevant in the European context since untreated CL lesions harbor vector infective parasites [305], which could infect competent/permissive vectors and allow the establishment of anthroponotic cycles for non-endemic *Leishmania* species. *Phlebotomus sergenti*, a specific vector of *L. tropica*, the species most imported with refugees, is widely distributed in Southern Europe [306] and new endemic foci could emerge through the introduction of infected humans in areas where the sand fly species are present [307]. Other phlebotomine species present in the Mediterranean region, namely *P. perniciosus* and *P. tobbi*, have also been shown to be susceptible to *L. tropica* infection under experimental conditions [308,309] and capable of transmitting viable parasites to vertebrate hosts (for *P. perniciosus*), representing an additional threat of its introduction in countries where these permissive sand fly species are present [310]. The movement of refugees across borders has also been linked to the detection of new *L. tropica* zymodemes in endemic areas. In Crete, where *L. tropica* infection is diagnosed sporadically [310], a new zymodeme was detected from an Afghan refugee and later on a local (non-traveler) dog [138]. DNA of non-endemic *Leishmania* species has been detected in phlebotomine sand flies in refugee camps in Greece [311].

Although the risk of introduction of *L. major* seems to be low, as its gerbillids reservoirs are not present in Europe [310,312], voles of the genus *Microtus* have recently been implicated as *L. major* reservoir hosts in a CL focus in northern Israel [313]. As such, the possibility that *L. major*, having adapted to voles, spreads north into Turkey and southern Europe, where reservoir hosts and vectors exist sympatrically should not be neglected. Furthermore, the presence of European sand fly species permissive to this parasite species, such as *P. perniciosus* [314] and *P. tobbii* [315], should also be kept in mind. Indeed, the presence of *L. major* in Europe has already been reported, namely in Portugal, through the detection of its DNA in sand flies [316] and a cat [317], and of *L. infantum*/*L. major* hybrids in HIV-infected patients [291] and a cat [317].

Phlebotomine vectors for *L. infantum* are also permissive for *L. donovani* and are widely distributed across Southern Europe, raising concern for the introduction of this parasite species, as has already been documented in Cyprus in humans and dogs [318]. Hybridization between *L. infantum* and *L. donovani* is also concerning and has been demonstrated in Turkey [319].

#### 3.1.3. Clinical Aspects and Management of Cutaneous Leishmaniasis

Regarding clinical presentation (Table 5), OW disease presented as single lesions in 58.8% of cases, preferentially located in the head and neck (50.6%) (especially in the cheeks—13/36, [119,126,137,138,148,153,168,175,232,242,246]—and nose—12/36, [45,113,126,132,134,145,152,153,196,230,246]) with the region of the trunk being the least affected (2.0%). Patients presented ulcerated lesions in 56.1% of cases, but other lesion types were also frequently seen, namely papules (24.9%) and plaques (20.2%). Besides cutaneous lesions, some patients presented lymphadenopathy (*n* = 21; 1.2%). Management strategy was described in 506 patients (Table 6): 282 (55.7%) received systemic treatment, 172 (34.0%) local treatment, 4 (0.8%) combined local and systemic treatment and 48 (9.5%) no treatment. A total of 65 failures or relapses were reported, representing 12.8% of cases [30,69,83,85,86,95,112,115,123,132,134,135,145,152,153,164,176,180,227,235,238,244]. When retreatment was described (*n* = 28, [30,83,85,86,112,115,123,134,135,145,152,164,176,180,227,235,238,244]), it most often consisted of a different strategy and/or drug (93.1%, [83,85,86,112,115,123,132,134,135,145,152,153,164,176,180,227,237,238,244]), especially systemic therapy (58.6%, [83,112,115,123,132,134,135,145,164,180,227,235,244]) (with LAmB being the drug of choice for 64.7% of these cases, [113,122,135,164,180,235]). Combination of drugs represented 34.5% of retreatments [30,112,115,135,238].

The clinical presentation for each of the three most common OW species/complex is summarized as follows:

- *L. donovani* complex: predominantly single lesions; involving mostly the head/neck and upper limbs, often non-ulcerative (especially plaques/crusts);
- *L. major*: predominantly multiple lesions; involving mostly the limbs, mostly ulcerative;
- *L. tropica*: single or multiple lesions; predominantly in the head/neck and upper limbs, often non-ulcerative.

Other species/complexes were less often reported, and their epidemiological and clinical features were:

- *L. aethiopica*: 13 cases reported [7,10,11,119,132,133,136,247,275,277], six of them imported from Ethiopia [10,11,136,247]; only three cases described lesions [119,132,247]: two of them as single lesions in the face or trunk [121,247] and one as multiple lesions in the face [132]; in two patients the lesions were papular/nodular [119,247] and in one it was a plaque/crust [132]; management strategy was described in six cases (one no treatment, [247], two topical with intralesional antimonial and cryotherapy, [277], and three systemic with miltefosine or LAmB, [119,132,133]). Relapses were not reported in any of the cases.

Most of the CL caused by NW *Leishmania* species were presented as single lesions (79.0%; *n* = 290) and was preferentially located in the upper (41.2%) and lower (34.5%) limbs (Table 5). The head and neck regions were affected in 28.1% of cases (especially the ears—11/31, [69,93,122,128,129,159,162,177,189,194,228]). Patients frequently presented ulcerated lesions (77.2% of cases). One hundred and twenty-eight patients (30.5%) presented lymphadenopathy. Management strategy was described in 603 patients: 521 (86.4%) received systemic treatment, 42 (7.0%) local treatment, 6 (1.0%) combined local and systemic treatment and 34 (5.6%) no treatment (Table 7). A total of 85 failures or relapses were reported, representing 14.1% of cases [20,30,69,77,82,92,96,97,100,116,120,121,125,126,128,129,139,140,146,150,165,173,177,178,179,180,194,228,269]. When retreatment was described (*n* = 44, [30,82,92,96,97,100,116,120,121,125,126,128,129,139,140,146,150,173,177,178,179,180,194,228,269]), it most often consisted of a different strategy and/or drug (92.3%, [30,82,92,96,97,100,116,120,121,125,126,128,129,139,140,146,150,173,177,178,179,180,194,228,269]), especially systemic therapy (89.7%, [30,77,82,92,96,97,100,116,120,121,126,128,129,139,146,168,177,179,180,194,228,269]) (with LAmB used in 51.4% of systemic retreatments, [97,100,113,126,129,180,194,228,269]). Combination of drugs represented 17.9% of retreatments [30,92,116,121,129,152,179,194].

For New World disease, relevant findings by species/complex can be summarized as follows, for the three most common species/complexes:

- *L. braziliensis* complex: predominantly single lesions; most often in the lower and upper limbs; commonly ulcerative; more frequently associated with lymphadenopathy;
- *L. guyanensis* complex: predominantly multiple lesions; involving mostly the upper limbs; ulcerative; often associated with lymphadenopathy;
- *L. mexicana* complex: predominantly single lesions; mostly in the head/neck; mostly ulcerative, but also frequently plaques/crusts.

Other species/complexes were less often reported, and their epidemiological and clinical features were:

- *L. naiffi*: nine cases were reported [12,69,106,133,136], two from French Guiana [69,136], 2 from Surinam [106] one from Brazil [133] and four from unspecified countries of Latin America; only three cases described lesions (2/3 single, 1/3 multiple; 1/3 upper limbs, 1/3 lower limbs, 1/3 face; 1/3 ulcer, 2/3 plaque/crust, [16,108]); of the three cases where management strategy was described [108], only one was treated, with topical paromomycin [133], and no failures/relapses were reported;
- *L. lainsoni*: only one case reported [69], imported from Brazil; multiple ulcerated lesions in the upper limb; no treatment description available;
- *L. martiniquensis*: only one case reported [69], imported from the French Caribbean; multiple papular lesions, in the upper limbs; classified as PKDL in the original article; no treatment description available.

Atypical cutaneous presentations were reported in 14 cases in both OW/NW: nine disseminated leishmaniasis [10,69,133,185,192,235,260], four PKDL [11,28,69] and one diffuse leishmaniasis [8]. Skin coinfection or superinfection was described in eight patients: seven bacterial [94,121,185,196,197] and one fungal [185]. *Staphylococcus aureus* [185,197] and *Streptococcus pyogenes* [185] were the most common pathogens identified (2 cases each). 

#### 3.1.4. Particular Aspects of Mucocutaneous and Mucosal Leishmaniasis

Ninety cases of MCL and 39 cases of ML were reported (Table 8). Infection in these cases occurred in 22 different countries, with approximately half of MCL/ML cases (when country of infection was known) reported from Bolivia (*n* = 25|5, [30,77,82,107,108,173,180,228] and Spain (*n* = 6|12, [11,69,109,130,132,135,153,155,158,245]. Other countries (1 to 7 cases) were: Greece [11,69], Italy [69], France [20,69], Croatia [111], Peru [11,20,80,177], Ecuador [8,11], Brazil [9,102], Colombia [82], Venezuela [95,281], Suriname [12,64], French Guiana [66,69], Argentina [179], Belize [82,223], Nicaragua [69], Panama [154], Costa Rica [14,69,74], Cameroon [11], India [6], Turkey [69], Saudi Arabia [114].

Percentage of cases presenting as MCL/ML varied by species: 8.8% of individuals infected with *L. braziliensis* complex presented MCL/ML; for *L. donovani* complex, a disease with mucosal involvement was present in 6.0% of total cases (including VL), but 9.6% of skin/mucosal infection. In the OW, MCL/ML represented 0.6% of cutaneous/mucosal cases, although it represented 14.3% when considering Europe alone; in the NW, MCL/ML represented 3.8%. In countries with over 20 cases of leishmaniasis reported, presentation of the disease with mucosal involvement or progression to this form of disease was more common in the following countries: Suriname (where it represented 15.9% of total cases), Bolivia (12.7%), Spain (9.7%), France (12.5%) and Greece (13.9%).

For MCL, the median age was 37 years old and 74.6% of patients were male. Infection likely occurred in the New World for 80.6% of individuals, mostly in South America. Old World cases mostly originated from Europe/Central Asia (85.7% of OW MCL cases). 

Of 38 patients where the description of the location of mucosal lesions was available, exclusively nasal involvement was more common (26/38). In 43 cases, clinical aspects of cutaneous lesions were described. As the time of onset of mucosal lesions after cutaneous lesions varied from simultaneous to 50 years [102], travelers, especially those traveling to the NW, who develop cutaneous lesions should be warned to remain vigilant for symptoms of mucosal involvement, including many years after travel. Cutaneous lesions in MCL cases were often multiple (52.6% of patients; average 3.58 lesions), ulcerative (69.6%) and located in the head or neck (60.0%). Nasal obstruction was the most reported symptom (57.9%), followed by nasal discharge (34.2%, with epistaxis in 30.8% of these cases). Other symptoms included lacrimal gland obstruction [171] and deafness [161] in one case each. Though lymphadenopathy and/or lymphangitis were reported in 11.1% of patients, lymph node samples were rarely used for the diagnosis (3.6%). Species/complex identification was performed and reported in 49 cases (Table 8). All cases of MCL where management strategy was known were treated (*n* = 51); systemic treatment was used in 96.0% of cases, most often LAmB (43.1%, [20,77,173,192]) and intramuscular antimonials (33.1%, [38,79,81,107,108,142,173,223]). A combination of drugs was used initially in five cases (9.8%, [30,102,179]). Fourteen failures or relapses were reported (27.5%, [20,30,82,129,146,153,173,177,179,180,228]); all of them were retreated with a different secondary regimen, including combination therapy [30,179].

For ML, the median age was 64 years old and 78.8% of patients were male. Infection likely occurred in the Old World for 76.9% of individuals (85.7% of these from Europe/Central Asia). Eleven different countries were identified as likely places of infection for 29 patients.

Of 27 patients where the description of the location of mucosal lesions was available, 10 (37.0%) had exclusively nasal involvement, eight had exclusively oral involvement (including four cases of tongue leishmaniasis), eight had laryngeal involvement and one intestinal involvement (no evidence of visceral disease) [26]. Hoarseness, nasal obstruction, and nasal discharge were frequently reported signs/symptoms (16–42%). In three cases, mucosal coinfection with *Candida* species was reported (oral/pharyngeal candidiasis, [158,177,204]). 

Species/complex identification was performed and reported in 30 cases (Table 8). Systemic therapy was used in all cases of ML where therapeutic approach was described (*n* = 24), most often LAmB (33.3%, [111,114,130,135,154,161,190]), miltefosine (33.3%, [132,152,158,173,180]) and intramuscular antimonials (25.0%, [76,82,109,133,233]). A combination of drugs was used initially in two cases (8.3%, [30]). Three failures or relapses were reported (12.5%, [30,133,135]); two of these cases were subsequently treated with a combination of LAmB and miltefosine [30,135].

**Figure 6:**
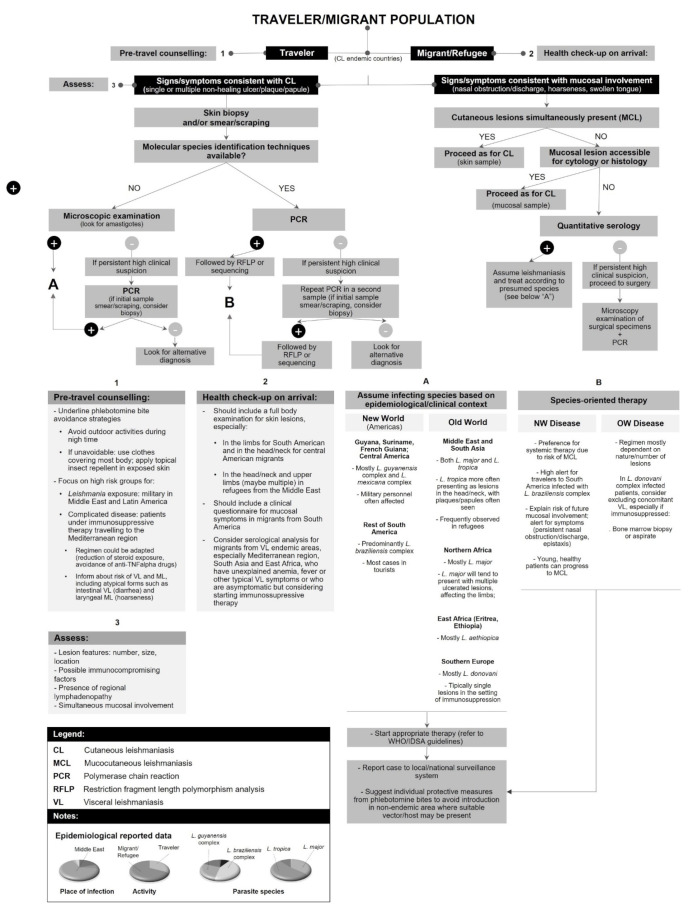
The proposed algorithm for the approach and management of CL/MCL/ML in non-endemic settings.

The analysis of all the CL cases allowed for a comparison of clinical presentation and outcome, which differed not only between OW and NW cases but also between species, as outlined in previous works [15,320]. It is also important to note that travel between endemic regions for different *Leishmania* species/strains can potentiate coinfection and lead to the generation of new hybrids, with different pathogenicity [291], the different clinical presentations with the consequent increase in the difficulty of diagnosing and management [321,322]. By combining this information with the relative abundance of different species in imported cases from each country, an approach for the diagnosis and management of CL, MCL and ML cases in non-endemic settings is suggested (Figure 6). This algorithm represents the authors’ opinions based on the results of this review and aims to help clinicians to judge the likely infecting species and adapt treatment strategies accordingly, in situations where laboratory confirmation of the infecting species/complex cannot be performed in clinically relevant time. Additionally, it should be noted that recommended treatments are not always available in non-endemic settings and, when available, medical teams may not be very familiar with their use.

#### 3.1.5. Particular Aspects in Immunosuppressed Patients

Data on the immunological status of patients was gathered whenever available, to emphasize the contribution of new immunosuppression factors to the progression of the disease and understand the changing epidemiology of leishmaniasis in non-endemic countries, similarly to endemic areas [323]. In immunosuppressed individuals, even when asymptomatic, *Leishmania* infection can be transmitted to phlebotomine vectors [324], and, in this way, these patients could play a role in the maintenance of the cycle of the parasite. 

A total of 214 leishmaniasis cases in patients with one or more recognized factors of immunosuppression were reported (Table 9). These patients represented 41.0% of ML cases, 21.7% of VL cases, 12.2% of MCL cases and 0.7% of CL cases.

Even though almost half of the immunosuppressed patients with VL were people living with HIV/AIDS, approximately one-fourth were patients chronically medicated with immunosuppressive drugs for inflammatory and auto-immune disorders. Of these, methotrexate, steroid and anti-TNFα were the most commonly implicated. In ML cases, immunosuppressive therapy was the most common form of immunosuppression (over half of cases), reinforcing that these patients represent one of the groups at risk of developing this clinical condition [5]. A high proportion of these cases (~25% of VL and ~50% of ML) were attributed to chronic medication with anti-TNFα. The association of anti-TNFα therapy with symptomatic *Leishmania* infection has been described in many case reports [325] and reviewed in previous articles [326]. Though the use of anti-TNFα has been increasing, no clinical trials have properly addressed the risk of progression to disease in previously and newly infected patients compared to non-medicated patients, and no strategies for the treatment of asymptomatic individuals have been prospectively researched, although clinical (and laboratory) monitoring could be suggested [323]. As such, no evidence currently supports the screening of individuals before starting treatment and there is no consensus on when and how to treat *Leishmania* infection in asymptomatic cases. Additionally, guidance on secondary prophylaxis in non-HIV immunosuppressed patients is lacking. Future research should address this gap of knowledge regarding appropriate prevention and management in emerging groups of immunosuppressed patients.

In addition, and as most of CL/MCL/ML in immunosuppressed patients was associated with infection in the Old World with the *L. donovani* complex, ear nose and throat clinicians should be aware of leishmaniasis as a differential diagnosis of oral and laryngeal lesions in immunocompromised people who traveled to *Leishmania* endemic areas, namely to the Mediterranean region. 

Most immunosuppressed patients received systemic treatment and relapse, or failure rates were higher than in the general population for both VL (21.6% vs. 13.7%) and CL/MCL/ML (22.2% vs. 12.8–14.1%) (Table 9). 

## 4. Conclusions

More than 10,000 cases of non-endemic leishmaniasis were reported in humans between 2000 and 2021, reflecting the impact of this disease on global tourism and migration and the movement of people (Box 1).

Drivers of changing patterns in epidemiology included the same as in endemic settings, namely conflict and war, as well as host factors such as immunosuppression (both natural and iatrogenic). Increased clinical management and surveillance of non-endemic cases (by physicians, especially dermatologists) could improve individual health and mitigate the public health risk of introducing *Leishmania* into new areas where favorable environmental conditions and permissive vectors exist. Strengthening surveillance and systematically combining animal and human data into an integrated platform, following a One Health approach (as proposed in a recent ECDC report [327]), could be the key to addressing the risk of leishmaniasis introduction associated with increased human and animal mobility.

Box 1 **Main findings.**

- Over 10,000 non-endemic cases of human leishmaniasis were reported in the literature from 2000–2021, most commonly CL, followed by VL and ML/MCL.
- VL resulted from travel to Europe in most cases; approximately half of the patients were children or elderly; fever, hepatosplenomegaly and pancytopenia were the most common findings; atypical presentations such as isolated lymphadenopathy, gastrointestinal and pulmonary involvement were described; the diagnosis was commonly made by microscopic examination of bone marrow biopsy/aspiration and/or serology; *L. donovani* complex was implied in almost all cases; LAmB was the drug most often used for treatment.
- Most CL cases were diagnosed in refugees from the Middle East, migrants from Latin America and South Asia and military personnel deployed in Asia; diagnosis relied on skin scraping and/or biopsy, with positivity rates higher for PCR than microscopy or culture; *L. tropica* and *L. major* were the two most common species in the Old World, while *L. braziliensis* complex and *L. guyanensis* complex were predominant in the New World; the number, type and location of lesions differed between species/complexes, as well as the therapeutic strategies used and the relapse rates reported; *L. aethiopica*, *L. naiffi*, *L. lainsoni* and *L. martiniquensis* infections were rarely described.
- MCL was reported in younger individuals, infected in the New World, most often by *L. braziliensis* complex; nasal mucosa was more often involved, and lymphadenopathy was common; the time between cutaneous and mucosal lesions varied from simultaneous to fifty years.
- ML was diagnosed mostly in older patients, infected in the Old World, most often by *L. donovani* complex; oral and laryngeal mucosa involvement was frequently described.
- Immunosuppressed patients represented a significant share of ML and VL cases; the two most common causes for immunosuppression were HIV/AIDS infection and chronic therapy, where anti-TNFα drugs represented the largest group; relapse/failure rates were higher in these patients.
- Non-endemic leishmaniasis represents an individual health problem, especially for refugees and immunosuppressed people; but also, a public health concern, related to the risk of introduction of the disease in new areas.

## Figures and Tables

**Table 1:** The epidemiological aspects of imported human leishmaniasis cases between 2000 and 2021.

Description	VL		CL/MCL/ML	
	Frequency	References	Frequency	References
Sex				
Male	72.2% (164/227)	[8,16,17,18,19,20,21,22,23,24,25,26,27,28,29,30,31,32,33,34,35,36,37,38,39,40,41,42,43,44,45,46,47,48,49,50,51,52,53,54,55,56,57,58,59,60,61,62,63,64,65,66,67,68]	58.2 (3820/6569)	[7,8,14,15,20,28,30,38,45,61,65,66,69,70,71,72,73,74,75,76,77,78,79,80,81,82,83,84,85,86,87,88,89,90,91,92,93,94,95,96,97,98,99,100,101,102,103,104,105,106,107,108,109,110,111,112,113,114,115,116,117,118,119,120,121,122,123,124,125,126,127,128,129,130,131,132,133,134,135,136,137,138,139,140,141,142,143,144,145,146,147,148,149,150,151,152,153,154,155,156,157,158,159,160,161,162,163,164,165,166,167,168,169,170,171,172,173,174,175,176,177,178,179,180,181,182,183,184,185,186,187,188,189,190,191,192,193,194,195,196,197]
Female	27.8% (63/227)	[8,20,26,30,31,32,44,45,65,66,67,198,199,200,201,202,203,204,205,206,207,208,209,210,211,212,213,214,215,216,217,218,219]	41.8% (2749/6569)	[8,9,20,28,30,45,61,65,72,73,75,77,81,82,85,87,90,91,95,100,101,112,115,116,118,126,132,133,134,136,139,148,152,158,164,166,169,172,173,175,178,185,188,191,192,195,220,221,222,223,224,225,226,227,228,229,230,231,232,233,234,235,236,237,238,239,240,241,242,243,244,245,246,247,248]
Median age (range)	34.5 year (4 months to 86 years)	[16,17,18,19,20,22,23,24,25,28,29,30,31,32,33,34,35,36,37,38,39,40,42,43,44,45,46,47,48,49,50,51,52,53,54,55,56,57,58,59,60,62,63,64,66,68,198,199,200,201,202,203,204,205,206,207,208,209,210,211,212,214,215,216,217,218,219,249,250,251,252,253]	31.0 year (9 months to 86 years)	[8,14,15,20,28,30,38,45,61,65,69,70,71,72,73,74,75,76,77,78,79,80,82,83,84,85,86,87,88,89,92,93,94,95,97,98,99,100,102,104,105,106,107,108,110,111,113,114,115,116,117,119,120,121,122,123,124,126,127,128,129,130,131,132,134,135,136,137,138,139,140,141,142,143,144,145,146,147,148,149,150,151,152,153,154,155,156,157,158,159,161,162,163,164,165,166,167,168,169,170,171,173,174,175,176,177,178,179,180,181,182,183,184,185,186,187,188,189,190,191,193,194,195,196,197,220,221,222,223,224,225,226,227,228,229,230,232,233,234,235,236,237,238,239,241,242,243,244,245,246,247,254,255]
Activity				
Traveler	85.2% (294/345) ^a^	[8,10,12,17,18,19,20,21,22,23,24,27,28,30,31,32,33,34,35,37,38,39,40,43,45,46,47,48,49,50,51,52,54,56,57,58,59,62,63,64,67,68,79,198,200,201,202,203,204,206,207,208,209,210,211,212,213,215,216,217,218,219,249,250,251,252,256,257,258,259,260,261,262]	32.5% (2338/7184) ^b^	[7,8,9,10,12,14,15,20,28,30,38,45,65,69,70,71,72,73,74,76,77,78,79,80,81,82,83,84,87,88,89,91,92,96,97,98,100,102,103,104,105,106,108,109,110,111,112,114,118,119,120,121,122,123,124,125,128,129,131,132,133,134,135,137,140,141,142,143,144,145,147,148,150,151,152,153,155,158,159,160,161,162,163,164,165,166,170,171,173,174,175,176,177,178,179,180,181,182,183,184,185,186,189,190,191,192,194,196,221,222,223,226,228,229,230,231,232,233,234,235,236,237,238,239,240,241,243,247,248,254,255,260,263,264,265,266,267,268,269,270,271,272,273]
Migrant/refugee	14.8% (50/345)	[7,8,16,17,20,25,28,29,30,44,55,60,208,214,216,261]	67.5% (4846/7184) ^c^	[8,9,15,20,28,30,45,85,91,95,101,108,115,117,130,132,138,139,149,154,157,158,168,172,181,187,188,193,195,197,220,242,244,246,274,275,276]
Region of infection				
Europe	61.2% (230/376)	[8,10,11,12,17,20,22,23,24,25,26,27,28,31,32,33,34,36,37,39,40,41,42,43,44,45,46,47,48,49,50,51,52,53,54,56,57,58,59,60,62,63,64,65,66,67,189,200,201,202,203,206,207,208,209,210,211,212,214,216,217,218,219,239,249,250,251,252,256,257,258,260,261,272]	2.6% (252/9619)	[8,10,11,15,20,26,28,45,61,65,66,87,104,109,111,112,124,126,130,132,133,135,143,152,155,156,158,162,190,191,192,207,217,220,222,227,228,230,240,249,263,264,268]
South Asia and East Asia	14.9% (56/376)	[10,19,20,21,259,261]	4.0% (383/9619)	[5,7,8,10,11,12,19,27,29,44,64,83,88,92,110,113,130,134,135,141,165,181,183,187,188,220,226,242,270,272,275,277,278]
Sub-Saharan Africa	9.8% (37/376)	[8,10,16,17,20,28,29,45,259]	2.2% (214/9619)	[8,9,10,11,12,15,20,30,61,81,95,99,119,123,133,136,148,185,192,234,247,254,272,273,277]
Middle East	9.0% (34/376)	[8,18,19,20,67]	64.3% (6189/9619)	[7,9,10,11,12,15,20,45,61,65,81,87,95,103,126,133,145,152,156,157,160,167,169,173,174,175,176,180,181,183,188,191,192,195,230,238,239,240,243,246,268,273,274,275,277,279]
Latin America and Caribbean	1.9% (7/376)	[10,55,57,65,199,204,253]	18.3% (1759/9619) ^d^	[7,8,9,10,11,12,14,15,20,28,30,61,65,69,70,71,72,73,74,75,76,77,78,79,80,81,82,84,87,88,89,90,91,92,93,96,97,98,100,101,102,105,106,107,108,110,116,118,120,121,122,126,128,129,131,132,133,136,139,140,141,142,143,147,149,150,151,154,159,162,163,165,166,167,170,173,175,177,178,179,180,182,184,185,186,189,190,191,193,194,197,221,223,224,228,236,237,241,263,264,266,267,269,270,272,273,276,277,280,281,282]
North Africa	1.9% (7/376)	[20,67,246]	5.6% (535/9619)	[6,8,9,10,11,12,15,20,30,61,81,83,95,132,136,137,148,161,164,169,175,192,230,231,255,268,272,273]
Central Asia	1.3% (5/376)	[44,61,67,205,215]	0.3% (26/9619)	[8,15,20,86,134,185,244,271,275,277]
Region of diagnosis, *n* (%)				
America	1.5% (8/540)	[21,43,49,51,55,60,215]	6.8% (662/9771) ^e^	[72,73,78,80,84,88,92,93,101,110,114,117,120,122,123,127,128,139,141,142,149,150,157,159,163,166,168,175,176,178,180,182,197,221,224,228,234,235,237,238,239,243,244,247,248,266,270,276,280,281]
Africa	3.1% (17/540)	[16]	NR	NA
Europe	80.4% (434/540)	[6,8,10,11,12,17,18,20,22,23,24,25,26,27,29,30,32,33,34,35,37,38,39,40,42,44,46,47,48,50,52,53,54,56,58,59,61,62,63,65,66,67,68,69,198,201,202,203,204,205,206,207,208,209,210,212,213,214,216,217,218,219,249,250,252,256,257,258,260,261,262,272]	33.1% (3235/9771)	[6,7,8,10,11,12,13,14,15,20,25,30,38,61,65,65,70,71,74,76,77,79,81,82,83,86,87,89,90,91,94,95,97,99,102,104,105,106,107,108,109,111,112,113,116,119,121,124,125,126,129,130,131,132,133,134,135,136,137,138,140,143,147,148,151,152,153,154,155,158,161,162,164,165,169,170,171,177,179,181,185,189,190,191,192,193,194,220,222,223,225,226,227,229,231,232,233,240,241,242,245,246,254,255,260,263,264,267,269,272,273,277,282]
Asia and Oceania	15.0% (81/540)	[19,28,36,45,57,64,211,251,259]	60.1% (5874/9771) ^f^	[28,45,69,75,85,88,100,102,115,118,144,145,146,156,160,167,172,173,174,183,184,186,187,188,195,196,230,264,268,271,274,275,278]

^a^ Including military (*n* = 3); ^b^ Including military (*n* = 1027); ^c^ Refugees (*n* = 4616); migrants (*n* = 186); refugee or migrant (unspecified, *n* = 44); ^d^ From South America (*n* = 1126); from Central America (*n* = 392); unspecified (*n* = 241); ^e^ North America (*n* = 612); Latin America and Caribbean (*n* = 50); ^f^ In the Middle East (*n* = 5762); in East Asia and Pacific (*n* = 105); in South Asia (*n* = 7); Abbreviations: VL, visceral leishmaniasis; CL, cutaneous leishmaniasis; MCL, mucocutaneous leishmaniasis; ML, mucosal leishmaniasis; NR, not reported.

**Table 2:** A clinical presentation of visceral leishmaniasis cases.

Description	Frequency	References
Clinical signs		
Fever	92.5% (172/186)	[16,17,18,19,20,21,22,23,25,27,29,30,31,32,33,36,38,40,43,44,46,47,48,49,50,51,52,53,54,56,57,59,60,62,63,64,66,68,199,200,202,203,207,208,209,210,211,212,214,215,216,218,219,249,250,251,252,256,258,259,260]
Hepatosplenomegaly	82.3% (153/186)	[16,17,18,19,20,21,22,23,25,27,29,30,31,32,33,36,38,40,43,44,46,47,48,49,50,51,52,53,54,56,57,59,60,62,63,64,66,68,199,200,202,203,207,208,209,210,211,212,214,215,216,218,219,249,250,251,252,256,258,259,260]
Constitutional signs	42.5% (79/186)	[35,48,55,217,252,259]
Gastrointestinal signs	15.6% (29/186)	[19,32,35,48,55,56,204,217,251,256,259]
Respiratory signs	18.3% (34/186)	[19,31,34,46,50,203,211,251,259]
Skin and mucosal haemorrhage	12.4% (23/186)	[35,48,55,217,252,259]
Lymphadenopathy	10.2% (19/186)	[35,37,38,56,59,198,205,213,250,256,259]
Skin lesions	4.8% (9/186)	[19,31,35,40,55,66,204,252,257]
Genitourinary signs	1.6% (3/186)	[19,29,64]
Edema	1.1% (2/186)	[35,218]
Ocular lesions	0.5% (1/186)	[35]
Laboratory findings		
Pancytopenia	64.0% (80/125)	[17,20,21,22,23,24,25,27,30,31,32,33,35,36,38,40,42,43,46,47,49,50,51,52,53,54,56,57,58,59,60,62,63,66,68,200,201,202,203,206,209,210,212,214,215,216,217,218,219,249,250,251,256,258]
Bicytopenia	23.2% (29/125) ^a^	[17,20,29,44,55,59,64,199,205,253]
Monocytopenia	10.4% (13/125) ^b^	[19,20,31,44,48,204,207,216,252]
Elevated liver enzymes	16.0% (20/125)	[19,21,22,32,36,43,48,50,51,59,62,64,202,203,212]
Hyperglobulinemia	11.2% (14/125)	[21,22,25,32,59,199,201,216,250,251,256]
Hypoalbuminemia	6.4% (8/125)	[21,22,29,46,201,204,210]
Renal failure	1.6% (2/125)	[39,48]
Others	20.0% (25/125) ^c^	[17,26,44,49,50,59,68,206,214,218]

^a^ Leukopenia + anemia (*n* = 13); anemia + thrombocytopenia (*n* = 13); ^b^ Anemia (*n* = 10); leukopenia (*n* = 1); thrombocytopenia (*n* = 2); ^c^ Hemophagocytic lymphohistiocytosis (*n* = 22); leucocytosis (*n* = 2); secondary myelofibrosis (*n* = 1).

**Table 4:** The therapeutic aspects of visceral leishmaniasis cases.

Treatment Strategy	Frequency of Total Cases	Frequency of Failure/Relapse Cases	References
Systemic	100% (256/256)	13.7% (35/256)	
Amphotericin B	74.8% (184/246)	12.0% (22/184)	[17,20,21,22,198,200,249,250,251]
Pentavalent antimonials	22.0% (54/246)	18.5% (10/54)	[8,16,18,19,32,40,44,216,256,257,260,261]
Miltefosine	0.8% (2/246)	50.0% (1/2)	[8,58]
Pentamidine	0.8% (2/246)	50.0% (1/2)	[17,257]
Paromomycin	0.8% (2/246)	50.0% (1/2)	[257]
Combination	0.8% (2/246) ^a^	NR	[36,50]
Local	NR	NA	NA
None	NR	NA	NA

^a^ Liposomal amphotericin B + miltefosine (*n* = 2); Abbreviations: NA, not applicable; NR, not reported.

**Table 5:** The clinical presentation of cutaneous New World and Old World leishmaniasis cases—global and by infecting species/complex.

Clinical Signs	New World								Old World							
	Total Cases	References	*L. braziliensis* Complex	References	*L. guyanensis* Complex	References	*L. mexicana* Complex	References	Total Cases	References	*L. donovani* Complex	References	*L. major*	References	*L. tropica*	References
Number of lesions																
Single	79.0% (290/367)	[9,15,29,69,70,72,73,75,77,79,82,87,88,92,96,98,100,101,106,116,121,122,128,129,131,139,150,151,158,165,166,167,173,175,179,182,185,186,189,193,194,197,221,223,263,266]	58.8% (57/97)	[15,28,75,78,87,98,100,116,121,129,166,167,173,175,181,221,223,265]	44.0% (22/50)	[9,15,73,117,139,151,179,182,266]	76.9% (10/13)	[15,87,92,122,129,151,186,189,194]	58.8% (321/546)	[9,15,30,61,66,85,87,95,115,119,132,137,138,143,144,152,153,162,167,169,171,175,185,196,220,222,225,227,232,240,242,246,247,255,260,273,275]	67.3% (33/49)	[15,87,95,116,133,137,143,144,153,162,227,229,231,235,265]	23.2% (26/112)	[9,15,30,94,169,171,185,240,255,273]	46.0% (23/50)	[9,15,87,95,139,167,175,246,275]
Multiple	21.0% (77/367)	[9,15,30,71,72,74,75,77,79,82,84,89,93,96,97,100,105,106,110,116,120,129,139,140,142,146,149,159,162,163,166,170,173,179,184,185,191,224,236,237,241,273]	41.2% (40/97)	[9,15,30,75,79,89,97,100,105,116,129,142,146,162,170,173,273]	56.0% (28/50)	[9,15,74,93,110,117,120,139,149,159,163,178,236,241]	23.1% (3/13)	[15,163]	41.2% (225/546)	[15,30,61,83,85,86,94,95,99,103,104,113,115,123,132,134,145,153,157,164,168,169,174,175,176,181,183,185,187,226,230,231,234,238,239,240,243,254,260,268,273,275]	32.7% (16/49)	[15,95,132,235]	76.8% (86/112)	[15,83,95,123,134,156,174,175,238,239,240,243,268,273]	54.0% (27/50)	[15,95,104,145,157,164,168,169,176,181,187,226,234]
Location of lesions																
Upper limbs	41.2% (142/345)	[15,30,45,71,72,77,82,89,93,96,97,98,100,105,110,116,120,126,129,139,141,149,150,165,166,173,175,179,182,184,185,191,193,197,221,228,241,263,266]	39.5% (34/86)	[15,30,45,89,97,98,100,105,116,129,141,166,173,175,221,228,263]	47.9% (23/48)	[15,73,93,110,117,120,126,139,149,163,178,182,241,266]	25.0% (4/16)	[15,45,150,166]	34.9% (531/1522)	[15,30,45,83,85,99,104,115,123,126,127,132,134,145,157,162,164,169,171,172,174,176,183,184,186,195,220,230,231,234,235,238,240,243,244,246,248,254,274,275]	31.3% (15/48)	[15,126,132,162,235]	51.2% (42/82)	[15,45,83,123,126,134,171,174,185,238,240,243]	47.1% (32/68)	[15,45,104,127,145,157,164,169,176,182,234,244,246]
Lower limbs	34.5% (119/345)	[15,30,45,69,70,72,73,74,77,82,84,87,106,116,120,121,126,129,139,140,146,151,159,163,166,167,170,173,185,191,224,228,236]	44.2% (38/86)	[15,30,45,116,120,129,166,167,170,173,228]	35.4% (17/48)	[15,73,74,117,120,139,151,159,163,236]	18.8% (3/16)	[15,87,163]	17.7% (270/1522)	[15,30,45,83,85,94,104,113,115,123,126,134,156,164,169,172,175,183,185,187,195,231,238,239,240,243,248,255,274,275]	22.9% (11/48)	[15,45,126]	40.2% (33/82)	[15,30,45,83,123,126,134,156,169,175,238,239,240,243,255]	20.6% (14/68)	[15,45,104,164,169,187]
Head and/or neck	28.1% (97/345)	[15,45,72,74,77,82,87,88,92,93,96,97,100,106,107,108,116,120,122,126,128,129,131,139,140,142,146,147,159,162,166,173,177,184,186,189,194,223,228,241]	20.9% (18/86)	[15,45,87,97,100,107,116,129,142,146,162,173,177,223,228]	33.3% (16/48)	[15,73,74,93,117,120,126,139,159,241,266]	56.3% (9/16)	[15,92,122,128,147,186,189,194]	50.6% (770/1522)	[15,45,85,86,87,113,115,119,123,126,132,134,137,139,143,144,145,152,153,157,164,167,168,172,175,183,184,185,195,206,220,222,225,226,227,230,232,235,240,242,244,245,246,248,274,275]	45.8% (22/48)	[15,87,132,137,143,144,153,181,196,225,227,229,232,235,245]	18.3% (15/82)	[15,123,126,134,240]	44.1% (30/68)	[15,45,87,139,145,157,164,167,168,175,181,226,244,246]
Trunk	8.1% (28/345)	[15,30,45,82,116,120,166,173,179,185,190,228]	5.8% (5/86)	[15,116,166,173,228]	6.3% (3/48)	[15,120,178]	6.3% (1/16)	[45]	2.0% (30/1522)	[15,45,85,123,132,134,149,164,171,172,185,195,231,240,243,244,247,248]	6.2% (3/48)	[15,132]	9.8% (8/82)	[15,45,134,149,240]	5.9% (4/68)	[164,244]
Type of lesions																
Ulcer	77.2% (271/351)	[15,30,61,69,70,71,72,73,77,78,79,82,84,87,88,89,93,96,97,98,101,105,106,107,108,110,116,120,122,127,129,132,139,140,147,151,159,165,166,167,170,175,179,185,191,194,197,223,236,237,241,245,263]	84.1% (65/77)	[15,30,78,79,87,97,98,105,107,116,121,129,167,170,175,223,263]	77.8% (35/45)	[15,73,93,110,117,120,139,151,159,178,236,241]	53.8% (7/13)	[122,128,166,178]	56.1% (270/481)	[15,30,61,83,85,94,99,103,104,113,122,127,132,134,143,145,156,160,162,164,168,169,171,174,176,182,183,185,187,231,234,235,238,239,240,242,243,244,245,246,248,254,255]	40.9% (18/44)	[15,116,132,143,162,235,245]	71.8% (61/85)	[15,30,83,123,134,156,171,174,185,238,239,240,243,255]	51.1% (24/47)	[15,104,127,145,164,167,168,169,176,181,187,234,244,246]
Lymphadenopathy	30.5% (128/420)	[15,75,77,79,82,97,100,105,108,110,121,125,129,142,147,159,165,166,177,178,228]	16.5% (15/91)	[75,79,97,100,105,121,129,142,177,228]	12.8% (6/47)	[110,117,159,179]	NR	NA	1.2% (21/1707)	[15,153,159,163,224,271]	3.4% (2/58)	[162,229]	NR	NA	1.4% (1/71)	[234]
Plaque/macular/crust	14.8% (52/351)	[15,61,82,106,107,116,120,131,141,142,150,159,162,163,179,197,241]	9.1% (7/77)	[15,107,116,141,142,162,166]	8.9% (4/45)	[15,120,159,178,241]	23.1% (3/13)	[15,150]	20.2% (97/481)	[15,85,115,132,134,137,152,153,157,164,168,169,175,196,227,235,240,244]	31.8% (14/44)	[15,132,137,153,196,227,235]	12.9% (11/85)	[15,134,169,240]	34.0% (16/47)	[15,157,164,168,169,175,244]
Papule/nodule	10.0% (35/351)	[15,82,84,92,93,109,116,139,140,149,162,163,179,183,184,185,193,221,224,266]	9.1% (7/77)	[116,162,221]	17.8% (8/45)	[15,93,110,139,149,163,182,266]	15.4% (2/13)	[15,93]	24.9% (120/481)	[15,30,83,86,87,104,119,132,134,144,145,152,169,171,172,174,175,185,187,190,220,222,225,226,234,245,246,247,268]	29.5% (13/44)	[15,87,132,144,190,225,229,232,245]	18.8% (16/85)	[15,134,171,174,175]	36.2% (17/47)	[15,88,104,145,169,187,226,234,246]
Other	1.7% (6/351)	[15,74,87]	1.3% (1/77)	[15]	NR	NA	7.7% (1/13)	[87]	1.5% (7/481)	[15]	NR	NA	NR	NA	2.1% (1/47)	[15]
Atypical presentations																
Disseminated	0.1% (2/1759)	[14,182]	NR	NA	0.7% (2/282)	[15,192]	NR	NA	0.06% (5/7860)	[10,15,235,260]	1.4% (4/280)	[10,15,235,260]	0.1% (1/923)	[192]	NR	NA
PKDL	0.1% (1/1759)	[15]	NR	NA	NR	NA	NR	NA	0.04% (3/7860)	[11,15,28]	0.7% (2/280)	[11,27]	NR	NA	NR	NA

Abbreviations: PKDL, Post Kala-azar dermal leishmaniasis; NA, not applicable; NR, not reported.

**Table 6:** Therapeutic aspects of cutaneous Old World leishmaniasis cases—global and by infecting species/complex.

Treatment Strategy	Total Cases			*L. donovani* Complex			*L. major*			*L. tropica*		
	Frequency of Cases	Frequency of Failure/Relapse Cases	References	Frequency of Cases	Frequency of Failure/Relapse Cases	References	Frequency of Cases	Frequency of Failure/Relapse Cases	References	Frequency of Cases	Frequency of Failure/Relapse Cases	References
Systemic	55.7% (282/506)	20.2% (57/282)		53.4% (47/88)	17.0% (8/47)		76.3% (87/114)	19.5% (17/87)		58.5% (24/41)	12.5% (3/24)	
Azole	28.7% (81/282)	40.7% (33/81)	[15,85,86,95,115,126,127,133,134,152,153,169,185,230,235,246]	10.6% (5/47)	100% (5/5)	[158,166,187]	31.0% (27/87)	33.3% (9/27)	[95,116,169]	37.5% (9/24)	22.2% (2/9)	[122,160,166,207,219,222]
Miltefosine	29.8% (84/282)	935% (8/84)	[15,112,132,133,155,158,160,162,180,183,232,240,255,260]	31.9% (15/47)	6.7% (1/15)	[132,133,162,229,232,255]	42.5% (37/87)	10.8% (4/37)	[128,240,255]	NR	NA	NA
Amphotericin B	24.5% (69/282)	23.2% (16/69)	[18,20,28,111,114,119,126,130,132,133,135,160,164,168,187,190,196,244,245,260]	42.5% (20/47)	10.0% (2/20)	[20,28,66,111,130,132,133,135,190,192,196,245]	11.5% (10/87)	40.0% (4/10)	[28,126,161,192]	62.5% (15/24)	6.7% (1/15)	[28,164,168,187,192,244]
Antimonials (intramuscular)	13.8% (39/282)	NR	[15,94,109,132,133,143,156,230,233]	10.6% (5/47)	NR	[109,132,133,143,233]	2.3% (2/87)	NR	[133,156]	NR	NA	NA
Combination	1.8% (5/282) ^a^	NR	[30,174,185]	NR	NR	NA	4.6% (4/87) ^b^	NR	[174,185]	NR	NA	NA
Pentamidine	1.4% (4/282)	NR	[20]	2.1% (1/47)	NR	[20]	8.0% (7/87)	NR	[81,135]	NR	NA	NA
Local	34.0% (172/506)	7.0% (12/172)		25.0% (22/88)	9.1% (2/22)		20.2% (23/114)	8.7% (2/23)		36.6% (15/41)	26.7% (4/15)	
Antimonials (intralesional)	50.6% (87/172)	8.0% (7/87)	[15,20,30,83,85,87,132,133,144,145,164,167,172,176,231,246,260]	36.4% (8/22)	NR	[20,87,132,144]	8.7% (2/23)	50% (1/2)	[30,83]	46.7% (7/15)	42.9% (3/7)	[133,145,164,167,176,246]
Paromomycin	20.3% (35/172)	8.6% (3/35)	[15,20,104,124,133,226,227,239,243,260]	22.7% (5/22)	20.0% (1/5)	[124,133,227]	69.6% (16/23)	NR	[166,190,202]	40.0% (6/15)	16.7% (1/6)	[20,104,133,226]
Combination	18.6% (32/172) ^c^	6.3% (2/32)	[15,123]	NR	NA	NA	4.3% (1/23) ^d^	100% (1/1)	[123]	NR	NA	NA
Cryotherapy, thermotherapy, excision	10.5% (18/172)	NR	[15,126,133,138,157,242,260]	40.9% (9/22)	11.1% (1/9)	[126,133,182]	17.4% (4/23)	NR	[126,133]	13.3% (2/15)	NR	[138,157]
None	9.5% (48/506)	8.3% (4/65)	[15,20,87,115,126,171,240,247,260]	21.6% (19/88)	NR	[20,126,260]	2.6% (3/114)	NR	[126,171,240]	2.4% (1/41)	NR	[87]
Systemic + local	0.8% (4/506) ^e^		[126,133,238]	NR	NA	NA	0.9% (1/114) ^f^	100% (1/1)	[238]	2.4% (1/41) ^g^	NR	[133]

^a^ Liposomal amphotericin B + miltefosine (*n* = 2); intramuscular pentavalent antimonial + ketoconazole (*n* = 3); ^b^ Liposomal amphotericin B + miltefosine (*n* = 1); intramuscular pentavalent antimonial + ketoconazole (*n* = 3); ^c^ Cryotherapy + intralesional pentavalent antimonial (*n* = 32); ^d^ Cryotherapy + intralesional pentavalent antimonial (*n* = 1); ^e^ Liposomal amphotericin B + intralesional pentavalent antimonial (*n* = 1); fluconazole + paromomycin + cryotherapy (*n* = 1); fluconazole + paromomycin (*n* = 1); itraconazole + cryotherapy (*n* = 1); ^f^ Fluconazole + paromomycin (*n* = 1); ^g^ Itraconazole + cryotherapy (*n* = 1); Abbreviations: NA, not applicable; NR, not reported.

**Table 7:** Therapeutic aspects of cutaneous New World leishmaniasis cases—global and by infecting species/complex.

Treatment Strategy	Total Cases			*L. braziliensis* Complex			*L. Guyanensis* Complex			*L. mexicana* Complex		
	Frequency of Cases	Frequency of Failure/Relapse Cases	References	Frequency of Cases	Frequency of Failure/Relapse Cases	References	Frequency of Cases	Frequency of Failure/Relapse Cases	References	Frequency of Cases	Frequency of Failure/Relapse Cases	References
Systemic	86.4% (521/603)	12.5% (52/521)		88.8% (199/224)	12.7% (25/199)		85.1% (40/47)	25.0% (10/47)		60.0% (9/15)	33.3% (3/9)	
Antimonials (intramuscular)	53.7% (280/521)	7.1% (20/280)	[15,20,30,69,71,72,73,75,76,77,78,79,80,82,87,89,92,100,101,105,107,108,116,120,125,133,142,165,166,173,223,263,266,269]	40.1% (81/199)	8.6% (7/81)	[30,75,76,79,87,89,100,105,107,116,133,142,166,173,221,223,263]	22.5% (9/40)	NR	[73,120,133,266]	11.1% (1/9)	100% (1/1)	[92]
Amphotericin B	28.4% (148/521)	16.9% (25/148)	[15,28,61,77,98,100,116,120,121,128,139,149,154,159,163,166,167,170,173,177,194,224,237]	52.3% (104/199)	14.8% (12/81)	[20,28,98,116,121,133,167,170,173,177]	35.0% (14/40)	42.9% (6/14)	[117,120,139,149,159,163]	66.7% (6/9)	33.3% (2/6)	[28,128,166,194]
Pentamidine	10.4% (26/521)	11.5% (3/26)	[15,61,96,133,151,241]	0.5 (1/199)	NR	[133]	17.5% (7/40)	NR	[133,151,241]	NR	NA	NA
Miltefosine	8.4% (44/521)	34.1% (15/44)	[15,93,116,126,129,133,140,162,173,178,180,228]	6.0% (12/199)	41.7% (5/12)	[116,129,162,173,180,228]	15.0% (6/40)	33.3% (2/6)	[93,126,133,178]	NR	NA	NA
Azole	2.1% (11/521)	9.1% (1/11)	[15,20,30,61,74,88,97,133]	0.5% (1/199)	100% (1/1)	[97]	10.0% (4/40)	50.0% (2/4)	[74,133]	11.1% (1/9)	NR	[20]
Combination	2.3% (12/521) ^a^	8.3% (1/12)	[15,30,84,102,132,179,185,186]	NR	NA	NA	NR	NA	NA	11.1% (1/9) ^b^	NR	[186]
Local	7.0% (42/603)	4.7% (2/42)		0.4% (1/224)	100% (1/1)		6.4% (3/47)	NR		6.7% (1/15)	NR	
Antimonials (intralesional)	45.2% (19/42)	5.3% (1/19)	[15,30,61,87,133,146,236,269]	NR	NA	NA	66.7% (2/3)	NR	[133,236]	100% (1/1)	NR	[87]
Combination	23.8% (10/42)	NR	[125,132]	NR	NA	NA	NR	NA	NA	NR	NA	NA
Paromomycin	11.9% (5/42)	20.0% (1/5)	[15,133]	100% (1/1)	100% (1/1)	[133]	NR	NA	NA	NR	NA	NA
Cryotherapy, thermotherapy, excision	9.1% (8/42)	NR	[110,185,191,193,269]	NR	NA	NA	33.3% (1/3)	NR	[110]	NR	NA	NA
None	5.6% (34/603)	2.9% (1/34)	[15,106,110,122,126,133,150,173,189]	9.8% (22/224)	NR	[173]	NR	NA	NA	26.7% (4/15)	25.0% (1/4)	[122,133,150,189]
Systemic + local	1.0% (6/603) ^c^	33.3% (2/6)	[30,61,96,146,147,182,184]	0.9% (2/224)	NR	[30,146]	2.1% (1/47)	NR	[182]	6.7% (1/15)	NR	[147]

^a^ Liposomal amphotericin B + miltefosine (*n* = 3); pentamidine + ketoconazole (*n* = 1); miltefosine + ketoconazole (*n* = 1); ^b^ Liposomal amphotericin B + azole (*n* = 1); ^c^ Cryotherapy + intralesional pentavalent antimonial (*n* = 8); excision + intralesional pentavalent antimonial (*n* = 2); Abbreviations: NA, not applicable; NR, not reported.

**Table 8:** The clinical aspects of mucocutaneous and mucosal leishmaniasis cases.

Description	Frequency in MCL	References	Frequency in ML	References
Sex				
Male	74.6% (47/63)	[14,30,38,65,74,77,80,82,102,107,108,142,146,153,173,177,179,180]	78.8% (26/33)	[30,76,109,111,114,130,132,135,154,155,158,161,173,180,190]
Female	25.4% (16/63)	[20,65,82,173,223,228,245]	21.2% (7/33)	[20,30,158,233]
Median age (range)	37 years (17 to 75 years)	[9,11,14,20,30,38,65,74,77,80,82,102,107,108,142,146,173,177,179,180,223,228,272]	64 years (24 to 84 years)	[20,30,76,82,109,111,114,130,132,135,154,155,158,161,173,180,190,233]
Region of infection				
New World	80.6% (58/72)	[9,11,12,20,30,65,74,77,80,82,102,107,108,142,146,173,177,179,180,223,228,272]	23.1% (9/39)	[30,76,82,133,154,173]
South America	89.5% (51/57)	[9,11,12,20,30,65,77,80,82,102,107,108,142,146,173,177,179,180,228,272]	87.5% (7/8)	[30,76,82,154,173]
Central America	10.5% (6/57)	[14,74,82,223]	12.5% (1/8)	[154]
Old World	19.4% (14/72)	[6,11,153,245]	76.9% (30/39)	[20,26,30,109,111,114,130,132,133,135,158,161,180,190,233,273]
Europe and Central Asia	85.7% (12/14)	[11,153,245]	85.7% (24/28)	[20,26,109,111,130,132,133,135,158,190,233,273]
Subsaharan Africa	7.1% (1/14)	[11]	NR	NA
South Asia	7.1% (1/14)	[6]	3.6% (1/28)	[30]
Middle East	NR	NA	7.1% (2/28)	[114,180]
North Africa	NR	NA	3.6% (1/28)	[161]
Species/complex				
*L. braziliensis* complex	67.3% (33/49)	[12,20,30,102,107,129,142,146,173,223,228]	20.0% (6/30)	[30,76,133,173]
*L.* (*Viannia*) sp.	16.3% (8/49)	[38,77,82]	3.3% (1/30)	[82]
*L. donovani* complex	12.2% (6/49)	[153,245]	70.0% (21/30)	[20,26,109,111,130,132,133,135,158,190,233,273]
*L. guyanensis* complex	4.1% (2/49)	[74]	NR	NA
*L. major*	NR	NA	3.3% (1/30)	[161]
*L. tropica*	NR	NA	3.3% (1/30)	[180]
Mucosal location				
Nasal only	68.4% (26/38)	[30,38,74,77,80,82,102,107,142,173,177,179,223,245]	37.0% (10/27)	[30,76,132,133,154,158,161,180]
Oral and nasal	15.8% (6/38)	[108,146,173]	NR	NA
Oral only	12.2% (2/38)	[129,173]	29.6% (8/27) ^a^	[111,114,133,135,173,190,233]
Pharyngeal	7.9% (3/38)	[82,180,228]	NR	NA
Laryngeal	2.6% (1/38)	[30]	29.6% (8/27)	[30,82,109,130,155,158]
Mucosal signs/symptoms				
Nasal obstruction	57.9% (22/38)	[38,74,82,102,107,108,173]	21.1% (4/19)	[154,158,180]
Nasal discharge	34.2% (13/38)	[38,74,82,102,107,108,173]	15.8% (3/19)	[76]
Localized edema	18.4% (7/38)	[77,80,82,102,107,223]	36.8% (7/)	[26,76,109,111,114,159]
Epistaxis	10.5% (4/38)	[38,107]	10.5% (2/19)	[154,161]
Palate perforation	7.9% (3/38)	[108,146]	10.5% (2/19)	[154,233]
Odynophagia	7.9% (3/38)	[82,180,228]	5.3% (1/19)	[82]
Cough	2.6% (1/38)	[179]	5.3% (1/19)	[158]
Dysphagia	2.6% (1/38)	[179]	10.5% (2/19)	[111,130]
Hoarseness	2.6% (1/38)	[179]	42.1% (8/19)	[30,82,109,130,155,158]
Nasal septum perforation	2.6% (1/38)	[107]	10.5% (2/19)	[154,161]
Lymphadenopathy/lymphangitis	11.1% (10/90)	[38,82,108,129,142,177,228]	2.6% (1/19)	[158]

^a^ Including in the tongue (*n* = 4); Abbreviations: MCL, mucocutaneous leishmaniasis; ML, mucosal leishmaniasis; NA, not available; NR, not reported.

**Table 9:** The types of immunosuppression and therapeutic outcomes of immunosuppressed patients with leishmaniasis.

Description	Frequency in VL	References	Frequency in CL	References	Frequency in MCL	References	Frequency in ML	References
Cases	21.7% (117/540)	[8,10,20,24,26,27,28,32,34,35,36,38,39,40,42,47,48,54,55,57,58,61,66,68,202,203,204,205,207,208,209,217,250,252,257,258,260,261]	0.7% (70/9642)	[8,15,20,28,30,66,119,126,132,133,192,224,260,277]	12.2% (11/90)	[8,15,26,153,192,245]	41.0% (16/39)	[15,20,30,132,135,158,190]
Immunosuppressive condition								
HIV	49.1% (56/114)	[8,10,20,26,28,32,35,36,47,55,61,66,68,205,250,257,261]	14.8% (8/54)	[28,30,260,277]	44.4% (4/9)	[8,192]	9.1% (1/11)	[135]
Therapy	25.4% (29/114)		44.4% (24/54)		33.3% (3/9)		72.7% (8/11)	
Methotrexate (±steroid)	34.5% (10/29)	[20,40,54,66,208,260,261]	4.2% (1/24)	[20]	NR	NA	12.5% (1/8)	[158]
Steroid only	24.1% (7/29)	[26,34,54,202,252,260,261]	NR	NA	NR	NA	50.0% (4/8)	[30,158]
Anti-TNFα (±other drugs)	20.7% (6/29) ^a^	[66,209,261]	58.3% (14/24) ^b^	[119,132]	33.3% (1/3) ^c^	[245]	25.0% (2/8) ^d^	[165,194]
Azatioprine (±steroid)	10.3% (3/29)	[27,48,261]	4.2% (1/24)	[126]	NR	NA	NR	NA
Unspecified	6.9% (2/29)	[10]	33.3% (8/24)	[66,192]	66.7% (2/3)	[192]	12.5% (1/8)	[20]
Other monoclonal antibody	3.4% (1/29) ^e^	[57]	NR	NA	NR	NA	NR	NA
Malignancy	12.3% (14/114) ^f^	[20,26,39,58,61,66,203,217,257,258,261]	NR	NA	11.1% (1/9) ^g^	[26,153]	18.2% (2/11) ^h^	[158,190]
Diabetes mellitus	10.5% (12/114)	[25,26,38,42,181,202,204,207,261]	38.9% (21/54)	[129,277]	11.1% (1/9)	[179]	NR	NA
Transplant	2.6% (3/114) ^i^	[24,257,260]	1.9% (1/54) ^j^	[224]	NR	NA	NR	NA
Region of infection								
Old World	98.2% (112/114)	[8,10,20,24,26,27,28,32,34,36,38,39,40,42,47,48,54,57,58,61,66,202,203,205,207,208,209,217,250,252,257,258,260,261]	91.3% (42/46) ^l^					[8,15,20,26,28,30,66,119,126,132,133,135,153,158,190,192,245,260]
New World	1.8% (2/114)	[55,204]	8.7% (4/46) ^l^					[8,15,133,192,224]
Species/complex								
*L. donovani* complex	100% (60/60)	[10,20,24,26,27,28,40,47,48,54,57,58,61,66,68,202,204,209,250,260,261]	88.9% (24/27) ^l^					[20,66,132,135,153,158,190,245,260]
*L. aethiopica*	NR	NA	7.4% (2/27) ^l^					[119,132]
*L. braziliensis* complex	NR	NA	3.7% (1/27) ^l^					[26]
Treatment strategy								
Systemic	100% (37/37) ^k^	[8,20,24,26,27,32,34,35,36,39,40,47,48,54,55,58,61,66,68,202,203,204,205,208,209,217,250,252,257,260]	92.6% (25/27) ^l^					[20,30,66,119,126,132,135,153,158,190,192,224,245]
Local	NR	NA	7.4% (2/27) ^l^					
Relapses	21.6% (8/37)	[10,32,35,47,55,58,66,252,257,260]	22.2% (6/27) ^l^					

^a^ Methotrexate + adalimumab (*n* = 3); methotrexate + etanercept (*n* = 1); azathioprine + infliximab (*n* = 1); methotrexate + unspecified (*n* = 1); ^b^ Etanercept (*n* = 12); methotrexate + adalimumab (*n* = 1); unspecified (*n* = 1); ^c^ Methotrexate + adalimumab (*n* = 1); ^d^ Methotrexate + adalimumab (*n* = 1); unspecified (*n* = 1); ^e^ Fingolimod (*n* = 1); ^f^ Lymphoma (not specified) (*n* = 3); chronic lymphocytic leukemia (*n* = 2); thymoma (*n* = 2); non-Hodgkin lymphoma (*n* = 1); T-cell lymphoma (*n* = 1); MALT lymphoma (*n* = 1); myelofibrosis (*n* = 1); myeloma (*n* = 1); breast (*n* = 1); testicular (*n* = 1); ^g^ Hepatocellular carcinoma (*n* = 1); ^h^ Acute lymphocytic leukemia; 1 non-small cell lung cancer (*n* = 1); ^i^ Kidney (*n* = 1); kidney + pancreas (*n* = 1); Allo-HSCT (*n* = 1); ^j^ Kidney (*n* = 1); ^k^ Liposomal amphotericin B (*n* = 33); 3 intramuscular pentavalent antimonial (*n* = 3); 1 liposomal amphotericin (*n* = 1); ^l^ CL + MCL + ML; Abbreviations: VL, visceral leishmaniasis; CL, cutaneous leishmaniasis; MCL, mucocutaneous leishmaniasis; ML, mucosal leishmaniasis; TNF, Tumor Necrosis Factor; HSCT, Hematopoietic stem cell transplant; MALT, mucosa-associated lymphoid tissue; NA, not applicable.

## Supplementary Materials

The following supporting information can be downloaded at: https://www.mdpi.com/article/10.3390/pathogens11080921/s1, Figure S1: Age distribution of visceral leishmaniasis cases; Figure S2: Age distribution of cutaneous, mucocutaneous and mucosal leishmaniasis cases; Table S1: References of cases of visceral leishmaniasis diagnosed, by country of travel or migration; Table S2: References of cases of cutaneous, mucocutaneous, and mucosal leishmaniasis diagnosed, by country of travel or migration; Table S3: References of identifications of species/complex by country in the Old and New Worlds.
