# Supplementary material for: Non-Endemic Leishmaniases Reported Globally in Humans between 2000 and 2021—A Comprehensive Review
Source: Pathogens. 2022 Aug 16;11(8):921. doi: 10.3390/pathogens11080921 (PMC9415673; doi:10.3390/pathogens11080921)
Supplement: Supplementary file 1 [file pathogens-11-00921-s001.zip › Supplementary-Table S1.pdf]

Supplementary Table S1. References of cases of visceral leishmaniasis diagnosed, by country of travel or migration.

Country (or department)	References
Afghanistan	[1,2]
Albania	[3-5]
Algeria	[6]
Angola	[3,7,8]
Argentina	[9]
Armenia	[10]
Azerbaijan	[11]
Bangladesh	[1,12]
Brazil	[3]
China	[8]
Croatia	[3,13-16]
Cyprus	[17-19]
Eritrea	[3,20]
Ethiopia	[13]
France	[3,8,21-26]
French Guiana	[27]
Georgia	[6]
Greece	[3,8,10,17,20,28-37]
India	[1,12,38]
Iran	[8]
Iraq	[5]
Italy	[8,10,13,16,17,20,23,39-48]
Kenya	[20]
Kosovo	[10]
Lybia	[5]
Macedonia	[10,32,49]
Malta	[8,15,17,32,49,50]
Mexico	[51]
Montenegro	[52]
Philippines	[3]
Portugal	[8,32,53,54]
Russia	[5,55]
Togo	[8]
Tunisia	[8]
Somalia	[56]
Spain	[3,6,8,10,15,17,20,22,23,26,32,37,45,49,57-73]
Sri Lanka	[1]
Sudan	[12,65]
Syria	[5]
United Arab Emirates	[20]
Yemen	[1,74]

References

1. Iqbal, J.; Hira, P.R.; Saroj, G.; Philip, R.; Al-Ali, F.; Madda, P.J.; Sher, A. Imported Visceral Leishmaniasis: Diagnostic Dilemmas and Comparative Analysis of Three Assays. *J Clin Microbiol* **2002**, *40*, 475–479, doi:10.1128/JCM.40.3.475-479.2001.

2. Centers for Disease Control and Prevention (CDC) Two Cases of Visceral Leishmaniasis in U.S. Military Personnel--Afghanistan, 2002-2004. *MMWR Morb Mortal Wkly Rep* **2004**, *53*, 265–268.

3. di Muccio, T.; Scalone, A.; Bruno, A.; Marangi, M.; Grande, R.; Armignacco, O.; Gradoni, L.; Gramiccia, M. Epidemiology of Imported Leishmaniasis in Italy: Implications for a European Endemic Country. *PLoS One* **2015**, *10*, e0129418, doi:10.1371/JOURNAL.PONE.0129418.

4. Offenbacher, R.; Rybinski, B.; Joseph, T.; Rahmani, N.; Boucher, T.; Weiser, D.A. An 8-Year-Old Boy With Fever, Splenomegaly, and Pancytopenia. *Pediatrics* **2020**, *146*,e20192372, doi:10.1542/PEDS.2019-2372.

5. Tzani, M.; Barrasa, A.; Vakali, A.; Georgakopoulou, T.; Mellou, K.; Pervanidou, D. Surveillance Data for Human Leishmaniasis Indicate the Need for a Sustainable Action Plan for Its Management and Control, Greece, 2004 to 2018. *Euro Surveill* **2021**, *26*, 2000159, doi:10.2807/1560-7917.ES.2021.26.18.2000159.

6. Aissaoui, N.; Hamane, S.; Gits-Muselli, M.; Petit, A.; Benderdouche, M.; Denis, B.; Alanio, A.; Dellière, S.; Bagot, M.; Bretagne, S. Imported Leishmaniasis in Travelers: A 7-Year Retrospective from a Parisian Hospital in France. *BMC Infect Dis* **2021**, *21*, 1–9.

7. Beltrame, A.; Arzese, A.; Camporese, A.; Rorato, G.; Crapis, M.; Tarabini-Castellani, G.; Boscutti, G.; Pizzolitto, S.; Calianno, G.; Matteelli, A.; et al. Acute Renal Failure Due to Visceral Leishmaniasis by *Leishmania infantum* Successfully Treated with a Single High Dose of Liposomal Amphotericin B. *J Travel Med* **2008**, *15*, 358–360, doi:10.1111/J.1708-8305.2008.00220.X.

8. Harms, G.; Schönian, G.; Feldmeier, H. Leishmaniasis in Germany. *Emerg Infect Dis* **2003**, *9*, 872-875, doi:10.3201/EID0907.030023.

9. Martín-Sánchez, J.; Navarro-Mari, J.M.; Pasquau-Liaño, J.; Salomón, O.D.; Morillas-Márquez, F. Visceral Leishmaniasis Caused by *Leishmania infantum* in a Spanish Patient in Argentina: What Is the Origin of the Infection? Case Report. *BMC Infect Dis* **2004**, *4*, 20, doi:10.1186/1471-2334-4-20.

10. Bode, S.F.N.; Bogdan, C.; Beutel, K.; Behnisch, W.; Greiner, J.; Henning, S.; Jorch, N.; Jankofsky, M.; Jakob, M.; Schmid, I.; et al. Hemophagocytic Lymphohistiocytosis in Imported Pediatric Visceral Leishmaniasis in a Nonendemic Area. *J Pediatr* **2014**, *165*, 147-153 doi:10.1016/J.JPEDS.2014.03.047.

11. Ahmed, A.A.; Li, W.; Livingston, R.A. Travelling with Visceral Leishmaniasis. *Clin Microbiol Infect* **2020**, *26*, 454–455, doi:10.1016/J.CMI.2019.10.025.

12. El-Moamly, A.; El-Sweify, M.; Hafeez, M. Performance of RK39 Immunochromatography and Freeze-Dried Direct Agglutination Tests in the Diagnosis of Imported Visceral Leishmaniasis. *Parasitol Res* **2012**, *110*, 349–354, doi:10.1007/S00436-011-2499-9.

13. Chalupa P; Vanista J; Nohynkova M. The Review of Imported Visceral Leishmaniosis in the Czech Republic. *Bratisl Lek Listy* **2001**, *102*, 84–91.

14. Posch, C.; Walochnik, J.; Gschnait, A.; Feichtinger, H.; Rappersberger, K. [Kala Azar - Lethal Course of Visceral Leishmaniasis. Synchronous Infection with *Leishmania donovani/infantum* Complex and *Leishmania major* in a Patient after Mediterranean Vacation]. *Hautarzt* **2012**, *63*, 947–951, doi:10.1007/S00105-012-2446-4.

15. Vécsei, A.; Kastner, U.; Trebo, M.; Kornmüller, R.; Picher, O.; Schratzberger-Vécsei, E.; Gadner, H. Pediatric Visceral Leishmaniasis in Austria: Diagnostic Difficulties in a Non-Endemic Region. *Wien Klin Wochenschr* **2001**, *113*, 102–106.

16. Benes, J.; Kabelková, M.; Holecková, P.; Kozák, T.; Benesová, K.; Mikulenková, D.; Nohýnková, E. [Visceral Leishmaniasis (Two Case Reports)]. *Klin Mikrobiol Infekc Lek* **2012**, *18*, 43–47.

17. Malik, A.N.J.; John, L.; Bryceson, A.D.M.; Lockwood, D.N.J. Changing Pattern of Visceral Leishmaniasis, United Kingdom, 1985-2004. *Emerg Infect Dis* **2006**, *12*, 1257–1259, doi:10.3201/EID1708.050486.

18. Auyeung, P.; French, M.A.; Hollingsworth, P.N. Immune Restoration Disease Associated with *Leishmania donovani* Infection Following Antiretroviral Therapy for HIV Infection. *J Microbiol Immunol Infect* **2010**, *43*, 74–76, doi:10.1016/S1684-1182(10)60011-4.

19. Mahendiran, T.; Doolub, G.; Nisbet, A. Fever in a Returning Traveller: Visceral Leishmaniasis Triggering Haemophagocytic Lymphohistiocytosis. *BMJ Case Rep* **2018**, *2018*, doi:10.1136/BCR-2018-224775.

20. Weitzel, T.; Mühlberger, N.; Jelinek, T.; Schunk, M.; Ehrhardt, S.; Bogdan, C.; Arasteh, K.; Schneider, T.; Kern, W. v.; Fätkenheuer, G.; et al. Imported Leishmaniasis in Germany 2001-2004: Data of the SIMPID Surveillance Network. *Eur J Clin Microbiol Infect Dis* **2005**, *24*, 471–476, doi:10.1007/S10096-005-1363-1.

21. Agteresch, H.J.; van't Veer, M.B.; Cornelissen, J.J.; Sluiters, J.F. Visceral Leishmaniasis after Allogeneic Hematopoietic Stem Cell Transplantation. *Bone Marrow Transplant* **2007**, *40*, 391–393, doi:10.1038/SJ.BMT.1705728.

22. Buonomano, R.; Brinkmann, F.; Leupin, N.; Boscacci, R.; Zimmermann, A.; Müller, N.; Fux, C.A. Holiday Souvenirs from the Mediterranean: Three Instructive Cases of Visceral Leishmaniasis. *Scand J Infect Dis* **2009**, *41*, 777–781, doi:10.1080/00365540903104127.

23. Bart, A.; van Thiel, P.P.; de Vries, H.J.; Hodiamont, C.J.; van Gool, T. Imported Leishmaniasis in the Netherlands from 2005 to 2012: Epidemiology, Diagnostic Techniques and Sequence-Based Species Typing from 195 Patients. *Euro surveillance* **2013**, *18*, 20544, doi:10.2807/1560-7917.ES2013.18.30.20544.~

24. van Raalte, D.; Wesselius, H.; Klerk, G. Unexpected Diagnosis of Visceral Leishmaniasis in a Patient Presenting with an Infected ICD Lead. *Neth J Med* **2014**, *72*, 146–148.

25. Haque, L.; Villanueva, M.; Russo, A.; Yuan, Y.; Lee, E.J.; Topal, J.; Podoltsev, N. A Rare Case of Visceral Leishmaniasis in an Immunocompetent Traveler Returning to the United States from Europe. *PLoS Negl Trop Dis* **2018**, *12*, e0006727, doi:10.1371/JOURNAL.PNTD.0006727.

26. T, R.; TF, W.; G, K.; AM, V.F. Visceral Leishmaniasis: Also Beware of These Deceptive Microbes in Non-Endemic Countries! *Arch Dis Child* **2001**, *84*, 373b–3373, doi:10.1136/ADC.84.4.373B.

27. Polley, S.D.; Watson, J.; Chiodini, P.L.; Lockwood, D.N.J. Visceral Leishmaniasis in Traveler to Guyana Caused by *Leishmania* siamensis, London, UK. *Emerg Infect Dis* **2018**, *24*, 155–156, doi:10.3201/EID2401.161428.

28. Ju, O.; Grove, D.I.; Jaksic, W.J.; Dart, G.W. Visceral Leishmaniasis: A Trip to the Greek Islands Is Not Always Idyllic. *Med J Aust* **2004**, *181*, 446–447, doi:10.5694/J.1326-5377.2004.TB06372.X.

29. Hoffmann-Tonn, K.; Geis, S.; Peckelsen, C.; Berna, G.; Fleischmann, E.; Bretzel, G.; Löscher, T. [Acute Febrile Disease with Splenomegaly and Pancytopenia. A 66-Year-Old Greek Patient with a Prosthetic Mitral Valve]. *Internist (Berl)* **2007**, *48*, 731–736, doi:10.1007/S00108-007-1873-2.

30. Stark, D.; van Hal, S.; Lee, R.; Marriott, D.; Harkness, J. Leishmaniasis, an Emerging Imported Infection: Report of 20 Cases from Australia. *J Travel Med* **2008**, *15*, 351–354, doi:10.1111/J.1708-8305.2008.00223.X.

31. Schmid, M.B.; Leichsenring, M.; Keller, C.; Hegasy, G. [Pancytopenia, Fever, and Splenomegaly in a 2-Year-Old Boy]. *Dtsch Med Wochenschr* **2009**, *134*, 1274–1277, doi:10.1055/S-0029-1225275.

32. Ehehalt, U.; Schunk, M.; Jensenius, M.; van Genderen, P.J.J.; Gkrania-Klotsas, E.; Chappuis, F.; Schlagenhauf, P.; Castelli, F.; Lopez-Velez, R.; Parola, P.; et al. Leishmaniasis Acquired by Travellers to Endemic Regions in Europe: A EuroTravNet Multi-Centre Study. *Travel Med Infect Dis* **2014**, *12*, 167–172, doi:10.1016/J.TMAID.2013.12.003.

33. Hamilton, A.; Kelleher, A.; Marriott, D. A Case of Severe Visceral Leishmaniasis Resulting from Travel to Greece. *BMJ Case Rep* **2009**, *2009*, doi:10.1136/BCR.06.2009.2036.

34. Dujardin, A.; de La Blanchardière, A.; Dina, J.; Stefic, K.; Ravel, C.; Bonhomme, J.; Verdon, R.; Fournier, A.L. Case Report: *Leishmania* and HIV Co-Diagnosis: How to Understand Medical History? *Front Immunol* **2021**, *12*, 669723, doi:10.3389/FIMMU.2021.669723.

35. Aardema, H.; Sijpkens, Y.W.J.; Visser, L.G. Pancytopenia in a Simultaneous Pancreas and Kidney Transplant Recipient: An Unexpected Cause - a Case of Visceral Leishmaniasis in a Transplant Recipient. *Clin Nephrol* **2009**, *71*, 460–462, doi:10.5414/CNP71460.

36. Cuperus, F.; Oosterwijk, P.; Vos, A.; Ramijn, J.; van Dobbenburgh, A.; Bisseling, T. [Visceral Leishmaniasis: Not Only a Tropical Disease]. *Ned Tijdschr Geneeskd* **2013**, *157*, A5958.

37. Söbirk, S.K.; Inghammar, M.; Collin, M.; Davidsson, L. Imported Leishmaniasis in Sweden 1993-2016. *Epidemiol Infect* **2018**, *146*, 1267–1274, doi:10.1017/S0950268818001309.

38. Fletcher, K.; Issa, R.; Lockwood, D.N.J. Visceral Leishmaniasis and Immunocompromise as a Risk Factor for the Development of Visceral Leishmaniasis: A Changing Pattern at the Hospital for Tropical Diseases, London. *PLoS One* **2015**, *10*, doi:10.1371/JOURNAL.PONE.0121418.

39. El-Ramli, R.; Koulaouzidis, A. An Unusual Cause for Anaemia. *J Gastrointestin Liver Dis* **2008**, *17*, 353.

40. Seilmaier, M.; Hecht, A.; Guggemos, W.; Adorf, D.; Gehbauer, G. [Pancytopenia, Hepatosplenomegaly and Dry Cough after Breast Cancer]. *Dtsch Med Wochenschr* **2009**, *134*, 1269–1273, doi:10.1055/S-0029-1225274.

41. Alcheikh, A.; Lynar, S.; Brown, C.; Lee, A.; Bryant, C. Unusual Case of Splenomegaly and Pancytopenia in a Returned Traveller. *Intern Med J* **2017**, *47*, 1325–1326, doi:10.1111/IMJ.13604.

42. Eichenberger, A.; Buechi, A.E.; Neumayr, A.; Hatz, C.; Rauch, A.; Huguenot, M.; Diamantis-Karamitopoulou, E.; Staehelin, C. A Severe Case of Visceral Leishmaniasis and Liposomal Amphotericin B Treatment Failure in an Immunosuppressed Patient 15 Years after Exposure. *BMC Infect Dis* **2017**, *17*, 81, doi:10.1186/S12879-017-2192-4.

43. Asbury, K.; Seville, M.T.; Pritt, B.; Scotch, A.; Rosenthal, A.; Grys, T.E.; Kelemen, K. Closing the Brief Case: The Unexpected Souvenir. *J Clin Microbiol* **2018**, *56*, e01414-e01417, doi:10.1128/JCM.01387-17.

44. Adamczick, C.; Dierig, A.; Welzel, T.; Schifferli, A.; Blum, J.; Ritz, N. Double Trouble: Visceral Leishmaniasis in Twins after Traveling to Tuscany - a Case Report. *BMC Infect Dis* **2018**, *18*, 495, doi:10.1186/S12879-018-3394-0.

45. Glans, H.; Hertting, O. [Leishmaniasis a Neglected but Serious Tropical Disease]. *Lakartidningen* **2020**, *117*, 19256.

46. Stefaniak, J.; Malgorzata, P.; Kacprzak, E.; Skoryna-Karcz, B. [Visceral Leishmaniasis]. *Przegl Epidemiol* **2003**, *57*, 341–348.

47. Visser, L.; Verweij, J. [Clinical Reasoning and Decision-Making in Practice. A Young Boy with Fever, Pancytopenia and an Enlarged Spleen]. *Ned Tijdschr Geneeskd* **2006**, *150*, 2169–2170.

48. Williams, E.; Isles, N.S.; Seemann, T.; Kilpatrick, T.; Grigg, A.; Leroi, M.; Howden, B.P.; Kwong, J.C. Case Report: Confirmation by Metagenomic Sequencing of Visceral Leishmaniasis in an Immunosuppressed Returned Traveler. *Am J Trop Med Hyg* **2020**, *103*, 1930–1933, doi:10.4269/AJTMH.19-0841.

49. Koster, K.L.; Laws, H.J.; Troeger, A.; Meisel, R.; Borkhardt, A.; Oommen, P.T. Visceral Leishmaniasis as a Possible Reason for Pancytopenia. *Front Pediatr* **2015**, *3*, 59, doi:10.3389/FPED.2015.00059.

50. Khanlari, B.; Bodmer, M.; Terracciano, L.; Heim, M.H.; Fluckiger, U.; Weisser, M. Hepatitis with Fibrin-Ring Granulomas. *Infection* **2008**, *36*, 381–383, doi:10.1007/S15010-007-6365-8.

51. Hicks, L.; Kant, P.; Tay, P.H.; Vincini, V.; Schuster, H.; Rotimi, O.; Maughan, N.; Jordan, C.; Moss, S.; Everett, S.; et al. Visceral Leishmaniasis Presenting with Intestinal Failure: A Case Report and Literature Review. *Eur J Gastroenterol Hepatol* **2009**, *21*, 117–122, doi:10.1097/MEG.0B013E32830E6FDB.

52. Suvajdžić, N.; Pavlović, M.; Mišić, S.; Čemerikić, V.; Atkinson, H.D.A.; Čolović, M. Secondary Myelofibrosis in Visceral Leishmaniasis--Case Report. *Haematologia (Budap)* **2001**, *31*, 167–171, doi:10.1163/15685590152492981.

53. Robibaro, B.; Funk, G.C.; Dekan, G.; Demetriou, D.; Ziesche, R.; Winkler, S.; Block, L.H. Unusual Microbes in Asthma Exacerbation: *Alcaligenes xylosoxidans* and *Leishmania*. *Eur Respir J* **2009**, *33*, 1216–1219, doi:10.1183/09031936.00123708.

54. Meijer, M.-J.; Kuiper-Kramer, E.; Overbosch, D.; Groeneveld, P.H.P. Fever from the USA or Portugal? *Neth J Med* **2005**, *63*, 367.

55. Peeters, E.; Verhulst, S.; Wojciechowski, M.; Vlieghe, E.; Jorens, P.; van Marck, V.; Ramet, J.; de Dooy, J. Visceral Leishmaniasis in a Child Infected with the Human Immunodeficiency Virus in a Non-Endemic Region. *J Trop Pediatr* **2011**, *57*, 493–495, doi:10.1093/TROPEJ/FMR024.

56. Boussery, G.; Boelaert, M.; van Peteghem, J.; Ejikon, P.; Henckaerts, K. Visceral Leishmaniasis (Kala-Azar) Outbreak in Somali Refugees and Kenyan Shepherds, Kenya. *Emerg Infect Dis* **2001**, *7*, 603–604, doi:10.3201/EID0707.010746.

57. Williams, M.J.; Korsheed, S.; Goddard, A.F.; Venkatesan, P. Hepatosplenomegaly in a Patient Returning from Spain. *Br J Hosp Med (Lond)* **2006**, *67*, 670, doi:10.12968/HMED.2006.67.12.22444.

58. Hagenah, G.C.; Wündisch, T.; Eckstein, E.; Zimmermann, S.; Holst, F.; Grimm, W.; Neubauer, A.; Lohoff, M. [Sepsis-like Disease in an Immunocompromised Patient with a Travel History to Mallorca]. *Internist (Berl)* **2007**, *48*, 727–730, doi:10.1007/S00108-007-1872-3.

59. Neghina, R.; Neghina, A.M.; Merkler, C.; Marincu, I.; Moldovan, R.; Iacobiciu, I. Importation of Visceral Leishmaniasis in Returning Romanian Workers from Spain. *Travel Med Infect Dis* **2009**, *7*, 35–39, doi:10.1016/J.TMAID.2008.10.002.

60. Skram, M.K.; Bjering, S.; Hermansen, N.O.; Dini, L.; Hellebostad, M. A 15-Month-Old Girl with Fever and Pancytopenia. *Tidsskr Nor Laegeforen* **2011**, *131*, 2482–2486, doi:10.4045/TIDSSKR.11.0387.

61. Mccusker, L.; Platt, J. An Unusual Case of Anaemia in an Octogenarian. *Age Ageing* **2012**, *41*, 696–698, doi:10.1093/AGEING/AFS075.

62. Moore, E.M.; Lockwood, D.N. Leishmaniasis. *Clin Med (Lond)* **2011**, *11*, 492-497, doi:10.7861/CLINMEDICINE.11-5-492.

63. Besada, E.; Njålla, R.J.; Nossent, J.C. Imported Case of Visceral Leishmaniasis Presenting as Pancytopenia in a Norwegian Patient Treated with Methotrexate and Etanercept for Psoriasis Arthritis. *Rheumatol Int* **2013**, *33*, 2687–2689, doi:10.1007/S00296-012-2483-4.

64. Watkins, E.R.; Shamasunder, S.; Cascino, T.; White, K.L.; Katrak, S.; Bern, C.; Schwartz, B.S. Visceral Leishmaniasis-Associated Hemophagocytic Lymphohistiocytosis in a Traveler Returning from a Pilgrimage to the Camino de Santiago. *J Travel Med* **2014**, *21*, 429–432, doi:10.1111/JTM.12145.

65. Roberts, T.; Barratt, J.; Sandaradura, I.; Lee, R.; Harkness, J.; Marriott, D.; Ellis, J.; Stark, D. Molecular Epidemiology of Imported Cases of Leishmaniasis in Australia from 2008 to 2014. *PLoS One* **2015**, *10*, e0119212, doi:10.1371/JOURNAL.PONE.0119212.

66. Johnson, S.M.; Gilmour, K.; Samarasinghe, S.; Bamford, A. Haemophagocytic Lymphohistiocytosis Complicating Visceral Leishmaniasis in the UK: A Case for Detailed Travel History, a High Index of Suspicion and Timely Diagnostics. *BMJ Case Rep* **2019**, *12*, e228307, doi:10.1136/BCR-2018-228307.

67. Schwartz, T.; Jensenius, M.; Blomberg, B.; Fladeby, C.; Mæland, A.; Pettersen, F.O. Imported Visceral Leishmaniasis and Immunosuppression in Seven Norwegian Patients. *Trop Dis Travel Med Vaccines* **2019**, *5*, 16 doi:10.1186/S40794-019-0092-X.

68. Minakaran, N.; Soorma, T.; Ladhani, S.N. Visceral Leishmaniasis in a UK Toddler Following a Short Trip to a Popular Holiday Destination in Spain. *Case Rep Infect Dis* **2014**, *2014*, 1–3, doi:10.1155/2014/537052.

69. Field, V.; Gautret, P.; Schlagenhauf, P.; Burchard, G.D.; Caumes, E.; Jensenius, M.; Castelli, F.; Gkrania-Klotsas, E.; Weld, L.; Lopez-Velez, R.; et al. Travel and Migration Associated Infectious Diseases Morbidity in Europe, 2008. *BMC Infect Dis* **2010**, *10*, 330, doi:10.1186/1471-2334-10-330.

70. Trück, J.; Rampton, C.; Kelly, S.J.; Kelly, D. Visceral Leishmaniasis in an Infant Following a Holiday Trip to Spain. *BMJ Case Rep* **2015**, *2015*, doi:10.1136/BCR-2015-209484.

71. Theodosiou, A.; Hiew, H.; Petridou, C. An Acute Presentation of Haemophagocytic Lymphohistiocytosis Due to Visceral Leishmaniasis in a British Adult Returning Traveller. *Acute Med* **2019**, *18*, 184–188.

72. Blomberg, B.; Müller, K.E.; Helgeland, L.; Fladeby, C.; Mørch, K. A Man in His 80s with Arthritis and Persistent Fever. *Tidsskr Nor Laegeforen* **2019**, *139*, doi:10.4045/TIDSSKR.18.0546.

73. Lübbert, C.; Opitz, B.M.; Harms-Zwingenberger, G.; Nietsch, H.H. [Fever, Pancytopenia, and Splenomegaly 8 Months after a Trip to Majorca Island (Spain)]. *Med Klin (Munich)* **2008**, *103*, 29–35, doi:10.1007/S00063-008-1003-5.

74. Mégarbane, B.; Bruneel, F.; Cazals-Hatem, D.; Adle-Biassette, H.; Houze, S.; Wolff, M.; Régnier, B. [A Strange Disease of the Plateaux. Visceral Leishmaniasis]. *Rev Med Interne* **2001**, *22 Suppl 2*, 219–222, doi:10.1016/S0248-8663(01)83653-5.
