# Supplementary material for: Non-Endemic Leishmaniases Reported Globally in Humans between 2000 and 2021—A Comprehensive Review
Source: Pathogens. 2022 Aug 16;11(8):921. doi: 10.3390/pathogens11080921 (PMC9415673; doi:10.3390/pathogens11080921)
Supplement: Supplementary file 1 [file pathogens-11-00921-s001.zip › Supplementary-Table S2.pdf]

**Supplementary Table S2.** References of cases of cutaneous, mucocutaneous, and mucosal leishmaniasis diagnosed, by country of travel or migration.

| Country (or department) | References |
|---|---|
| Afghanistan | [1,2,11–20,3,21–25,4–10] |
| Algeria | [3,9,14,26–31] |
| Argentina | [3,29,32] |
| Belize | [1,3,38–47,7,48–50,12,21,33–37] |
| Bolivia | [1,3,49,51–59,7,60–65,12,14,27,31,33,37,38] |
| Brazil | [1,3,68–75,14,27,37,59,60,65–67] |
| Burkina Faso | [9,14,27,33,60,76,77] |
| Cameroon | [18] |
| Chad | [14,33,60,78] |
| Chile | [79] |
| Colombia | [2,3,23,27,49,60,62,71,80] |
| Costa Rica | [2,3,59,62,71,81–87,14,88–92,16,18,24,27,30,31,37] |
| Croatia | [93,94] |
| Cuba | [33] |
| Cyprus | [3,95] |
| Ecuador | [1,3,74,87,96,14,18,24,27,38,59,60,71] |
| Egypt | [1,3,14,29,31] |
| Eritrea | [3,37,97] |
| Ethiopia | [3,18,30,60,98] |
| France | [1,3,14,37,60,92] |
| French Guiana | [1,2,51,59,65,99–102,3,7,21,26,27,30,31,33] |
| Greece | [3,11,18,38,45,60,103–105] |
| Guatemala | [1,3,14,24,60,71,74,106,107] |
| Guyana | [3,14,71] |
| Honduras | [108] |
| India | [12,27,65,76] |
| Iran | [2,3,20,37] |
| Iraq | [2,3,5,12,20,21,37,60] |
| Israel | [3,9,111–113,14,31,37,60,74,92,109,110] |
| Italy | [1,2,115,3,14,29,31,37,92,104,114] |
| Jordan | [37,45,60,74,76] |
| Kenya | [1,14,37] |
| Kuwait | [5] |
| Lybia | [1,3,60] |
| Macedonia | [14] |
| Mali | [3,9,31,116] |
| Malta | [1–3,24,37,104] |
| Mauritania | [3,9,31,74] |
| Mexico | [2,3,120,16,26,31,60,71,117–119] |
| Montenegro | [18] |
| Morocco | [3,9,74,78,92,113,121–125,14,24,27,30,31,37,38,60] |
| Namibia | [108] |
| Nepal | [33] |
| Nicaragua | [3,126] |
| Niger | [26] |
| Oman | [108] |
| Pakistan | [2,11,12,22,23,37,127,128] |
| Panama | [2,3,14,18,27,61,74,129,130] |
| Paraguay | [131–133] |
| Peru | [1,2,33,37,59,60,65,87,92,113,132,134,3,135–140,12,14,18,24,27,30,31] |
| Portugal | [37,76] |
| Tanzania | [108] |
| Thailand | [60] |

| | |
|---|---|
| Tunisia | [3,9,141,14,18,26,30,31,38,60,78] |
| Turkey | [1,3,142,143] |
| Saudi Arabia | [3,28,37,144–150] |
| Senegal | [3,9,14,31,92,151] |
| Spain | [1,2,103,104,152–159,3,160–162,14,18,31,34,37,38,73] |
| Sri Lanka | [60,163] |
| Sudan | [3,14,24,60] |
| Suriname | [3,7,21,37,66,164] |
| Syria | [1,2,38,60,115,141,165–170,3,171,14,18–20,23,26,37] |
| Turkmenistan | [3,14,20,33] |
| United Arab Emirates | [1,3,38] |
| Uzbekistan | [76,172] |
| Venezuela | [19,24,62,76,173,174] |
| Yemen | [60] |

## References


1. Harms, G.; Schönian, G.; Feldmeier, H. Leishmaniasis in Germany. *Emerg. Infect. Dis.* **2003**, *9*, 872, doi:10.3201/EID0907.030023.
2. Roberts, T.; Barratt, J.; Sandaradura, I.; Lee, R.; Harkness, J.; Marriott, D.; Ellis, J.; Stark, D. Molecular Epidemiology of Imported Cases of Leishmaniasis in Australia from 2008 to 2014. *PLoS One* **2015**, *10*, doi:10.1371/JOURNAL.PONE.0119212.
3. Guery, R.; Walker, S.L.; Harms, G.; Neumayr, A.; Van Thiel, P.; Gangneux, J.P.; Clerinx, J.; Söbirk, S.K.; Visser, L.; Lachaud, L.; et al. Clinical Diversity and Treatment Results in Tegumentary Leishmaniasis: A European Clinical Report in 459 Patients. *PLoS Negl. Trop. Dis.* **2021**, *15*, doi:10.1371/JOURNAL.PNTD.0009863.
4. Rayatt, S.S.; Moss, A.L.H. Cutaneous Leishmaniasis. *Br. J. Plast. Surg.* **2000**, *53*, 443–445, doi:10.1054/BJPS.1999.3239.
5. Update: Cutaneous Leishmaniasis in U.S. Military Personnel --- Southwest/Central Asia, 2002--2004 Available online: https://www.cdc.gov/mmwr/preview/mmwrhtml/mm5312a4.htm (accessed on 13 July 2022).
6. Storer, E.; Wayte, J. Cutaneous Leishmaniasis in Afghani Refugees. *Australas. J. Dermatol.* **2005**, *46*, 80–83, doi:10.1111/J.1440-0960.2005.00146.X.
7. Zeegelaar, J.E.; Steketee, W.H.; Van Thiel, P.P.A.M.; Wetsteyn, J.C.F.M.; Kager, P.A.; Faber, W.R. Changing Pattern of Imported Cutaneous Leishmaniasis in the Netherlands. *Clin. Exp. Dermatol.* **2005**, *30*, 1–5, doi:10.1111/J.1365-2230.2004.01677.X.
8. Ather, S.; Chan, D.S.; Leaper, D.J.; Harding, K.G. Case Report and Literature Review of Leishmaniasis as a Cause of Leg Ulceration in the United Kingdom. *J. Wound Care* **2006**, *15*, 389–391, doi:10.12968/JOWC.2006.15.9.26957.
9. Morizot, G.; Delgiudice, P.; Caumes, E.; Laffitte, E.; Marty, P.; Dupuy, A.; Sarfati, C.; Hadj-Rabia, S.; Darie, H.; Le Guern, A.S.; et al. HEALING OF OLD WORLD CUTANEOUS LEISHMANIASIS IN TRAVELERS TREATED WITH FLUCONAZOLE: DRUG EFFECT OR SPONTANEOUS EVOLUTION? *Am. J. Trop. Med. Hyg.* **2007**, *76*, 48–52, doi:10.4269/AJTMH.2007.76.48.
10. Flaig, M.J.; Rupec, J.; Ruzicka, T.; Rupec, R.A. [Topical Treatment of Persistent Cutaneous Leishmaniasis with Paromomycin]. *Hautarzt.* **2007**, *58*, 689–692, doi:10.1007/S00105-007-1297-X.
11. Stark, D.; Van Hal, S.; Lee, R.; Marriott, D.; Harkness, J. Leishmaniasis, an Emerging Imported Infection: Report of 20 Cases from Australia. *J. Travel Med.* **2008**, *15*, 351–354, doi:10.1111/J.1708-8305.2008.00223.X.
12. Wall, E.C.; Watson, J.; Armstrong, M.; Chiodini, P.L.; Lockwood, D.N. Epidemiology of Imported Cutaneous Leishmaniasis at the Hospital for Tropical Diseases, London, United Kingdom: Use of Polymerase Chain Reaction to Identify the Species. *Am. J. Trop. Med. Hyg.* **2012**, *86*, 115–118, doi:10.4269/AJTMH.2012.10-0558.
13. Fathi, R.; Fathi, A. A Case of Cutaneous Leishmaniasis Found in Indiana. *Dermatol Online J.* **2013**, *19*, 18982.
14. Mosimann, V.; Neumayr, A.; Hatz, C.; Blum, J.A. Cutaneous Leishmaniasis in Switzerland: First Experience with Species-Specific Treatment. *Infection* **2013**, *41*, 1177–1182, doi:10.1007/S15010-013-0500-5.

15. Ntais, P.; Christodoulou, V.; Tsirigotakis, N.; Dokianakis, E.; Dedet, J.P.; Pratlong, F.; Antoniou, M. Will the Introduction of Leishmania Tropica MON-58, in the Island of Crete, Lead to the Settlement and Spread of This Rare Zymodeme? *Acta Trop.* **2014**, *132*, 125–130, doi:10.1016/J.ACTATROPICA.2014.01.003.

16. AK, B.; J, G.; M, L.; BJ, W.; A, M.; J, H.; W, G.; J, V.; S, K.; DO, F.; et al. Travel-Acquired Infections in Canada: CanTravNet 2011-2012. *Can. Commun. Dis. Rep.* **2014**, *40*, 313–325, doi:10.14745/CCDR.V40I16A01.

17. Schüürmann, M.; Kunz, M.; Lübbert, C. Oriental Sore (Old-World Cutaneous Leishmaniasis). *Dtsch. Arztebl. Int.* **2017**, *114*, 754, doi:10.3238/ARZTEBL.2017.0754B.

18. Söbirk, S.K.; Inghammar, M.; Collin, M.; Davidsson, L. Imported Leishmaniasis in Sweden 1993-2016. *Epidemiol. Infect.* **2018**, *146*, 1267–1274, doi:10.1017/S0950268818001309.

19. Imai, K.; Tarumoto, N.; Amo, K.; Takahashi, M.; Sakamoto, N.; Kosaka, A.; Kato, Y.; Mikita, K.; Sakai, J.; Murakami, T.; et al. Non-Invasive Diagnosis of Cutaneous Leishmaniasis by the Direct Boil Loop-Mediated Isothermal Amplification Method and MinION$^{TM}$ Nanopore Sequencing. *Parasitol. Int.* **2018**, *67*, 34–37, doi:10.1016/J.PARINT.2017.03.001.

20. Özbilgin, A.; Gencoglan, G.; Tunali, V.; Çavuş, İ.; Yıldırım, A.; Gündüz, C.; Harman, M. Refugees at the Crossroads of Continents: A Molecular Approach for Cutaneous Leishmaniasis Among Refugees in Turkey. *Acta Parasitol.* **2020**, *65*, 136–143, doi:10.2478/S11686-019-00139-4.

21. Herremans, T.; Pinelli, E.; Casparie, M.; Nozari, N.; Roelfsema, J.; Kortbeek, L. Increase of Imported Leishmaniasis in the Netherlands: A Twelve Year Overview (1996-2007). *Int. Health* **2010**, *2*, 42–46, doi:10.1016/J.INHE.2009.12.005.

22. Mohammadi, A.; Khamesipour, A.; Khatami, A.; Javadi, A.; Nassiri-Kashani, M.; Firooz, A.; Dowlati, Y.; Behnia, M.; Eskandari, S. Cutaneous Leishmaniasis in Suspected Patients Referred to the Center for Research and Training in Skin Diseases and Leprosy, Tehran, Iran from 2008 to 2011 - PubMed. *Iran J Parasitol* **2013**, *8*, 430–436.

23. Iannone, M.; Oranges, T.; Dini, V.; Romanelli, M.; Janowska, A. Wound Management Strategy for Treatment of Localized Cutaneous Leishmaniasis Using the TIME Framework. *Wounds* **2021**, *33*, E6–E9.

24. Field, V.; Gautret, P.; Schlagenhauf, P.; Burchard, G.D.; Caumes, E.; Jensenius, M.; Castelli, F.; Gkrania-Klotsas, E.; Weld, L.; Lopez-Velez, R.; et al. Travel and Migration Associated Infectious Diseases Morbidity in Europe, 2008. *BMC Infect. Dis.* **2010**, *10*, doi:10.1186/1471-2334-10-330.

25. Van Thiel, P.P.A.M.; Leenstra, T.; Kager, P.A.; De Vries, H.J.; Van Vugt, M.; Van Der Meide, W.F.; Bart, A.; Zeegelaar, J.E.; Van Der Sluis, A.; Schallig, H.D.F.H.; et al. Miltefosine Treatment of Leishmania Major Infection: An Observational Study Involving Dutch Military Personnel Returning from Northern Afghanistan. *Clin. Infect. Dis.* **2010**, *50*, 80–83, doi:10.1086/648726.

26. El Hajj, L.; Thellier, M.; Carrière, J.; Bricaire, F.; Danis, M.; Caumes, E. Localized Cutaneous Leishmaniasis Imported into Paris: A Review of 39 Cases. *Int. J. Dermatol.* **2004**, *43*, 120–125, doi:10.1111/J.1365-4632.2004.01991.X.

27. Pérez-Ayala, A.; Norman, F.; Pérez-Molina, J.A.; Herrero, J.M.; Monge, B.; López-Vélez, R. Imported Leishmaniasis: A Heterogeneous Group of Diseases. *J. Travel Med.*

**2009**, *16*, 395–401, doi:10.1111/J.1708-8305.2009.00341.X.

28. Yang, Y.-T.; Zhang, M.; Gao, C.-H.; Shi, F.; Guan, L.-R.; Wang, J.-Y. [Detection and Species Identification of Two Imported Cases of Cutaneous Leishmaniasis] - PubMed. *Zhongguo ji sheng chong xue yu ji sheng chong bing za zhi* **2011**, *29*, 461–464.

29. Neumayr, A.L.C.; Walter, C.; Stoeckle, M.; Braendle, N.; Glatz, K.; Blum, J.A. Successful Treatment of Imported Mucosal Leishmania Infantum Leishmaniasis with Miltefosine after Severe Hypokalemia under Meglumine Antimoniate Treatment. *J. Travel Med.* **2012**, *19*, 124–126, doi:10.1111/J.1708-8305.2011.00572.X.

30. Lavergne, R.A.; Iriart, X.; Martin-Blondel, G.; Chauvin, P.; Menard, S.; Fillaux, J.; Cassaing, S.; Roques-Malecaze, C.; Arnaud, S.; Valentin, A.; et al. Contribution of Molecular Diagnosis to the Management of Cutaneous Leishmaniasis in Travellers. *Clin. Microbiol. Infect.* **2014**, *20*, doi:10.1111/1469-0691.12487.

31. Aissaoui, N.; Hamane, S.; Gits-Muselli, M.; Petit, A.; Benderdouche, M.; Denis, B.; Alanio, A.; Dellière, S.; Bagot, M.; Bretagne, S. Imported Leishmaniasis in Travelers: A 7-Year Retrospective from a Parisian Hospital in France. **2021**, *21*, 1–9.

32. Basile, G.; Cristofaro, G.; Locatello, L.G.; Vellere, I.; Piccica, M.; Bresci, S.; Maggiore, G.; Gallo, O.; Novelli, A.; Di Muccio, T.; et al. Refractory Mucocutaneous Leishmaniasis Resolved with Combination Treatment Based on Intravenous Pentamidine, Oral Azole, Aerosolized Liposomal Amphotericin B, and Intralesional Meglumine Antimoniate. *Int. J. Infect. Dis.* **2020**, *97*, 204–207, doi:10.1016/J.IJID.2020.06.003.

33. Kuna, A.; Gajewski, M.; Bykowska, M.; Pietkiewicz, H.; Olszański, R.; Myjak, P. Imported Cutaneous Leishmaniasis: A 13-Year Experience of a Polish Tertiary Center. *Postep. dermatologii i Alergol.* **2019**, *36*, 104–111, doi:10.5114/ADA.2019.82830.

34. van der Snoek, E.M.; Couwenberg, S.M.; Stijnis, C.; Kortbeek, L.M.; Schadd, E.M. Two Cases of Cutaneous Leishmaniasis in Dutch Military Personnel Treated with Oral Miltefosine. *J. R. Army Med. Corps* **2017**, *163*, 68–70, doi:10.1136/JRAMC-2015-000530.

35. Bailey, M.S.; Langman, G. Misdiagnosis of Cutaneous Leishmaniasis and Recurrence after Surgical Excision. *J. R. Army Med. Corps* **2014**, *160*, 314–316, doi:10.1136/JRAMC-2013-000123.

36. Demers, E.; Forrest, D.M.; Weichert, G.E. Cutaneous Leishmaniasis in a Returning Traveller. *CMAJ* **2013**, *185*, 681–683, doi:10.1503/CMAJ.120694.

37. Bart, A.; van Thiel, P.P.; de Vries, H.J.; Hodiamont, C.J.; Van Gool, T. Imported Leishmaniasis in the Netherlands from 2005 to 2012: Epidemiology, Diagnostic Techniques and Sequence-Based Species Typing from 195 Patients. *Euro Surveill.* **2013**, *18*, doi:10.2807/1560-7917.ES2013.18.30.20544.

38. Poeppl, W.; Oeser, C.; Grabmeier-Pfistershammer, K.; Walochnik, J.; Burgmann, H. Clinical Findings and Management of Imported Cutaneous Leishmaniasis: Report of 14 Cases from Austria. *Travel Med. Infect. Dis.* **2013**, *11*, 90–94, doi:10.1016/J.TMAID.2013.03.002.

39. Van Thiel, P.P.A.M.; Zeegelaar, J.E.; Van Gool, T.; Faber, W.R.; Kager, P.A. Cutaneous Leishmaniasis in Three Dutch Military Cohorts Following Jungle Training in Belize. *Travel Med. Infect. Dis.* **2011**, *9*, 153–160, doi:10.1016/J.TMAID.2011.03.001.

40. Holmes, W.J.M.; Tehrani, H.; Liew, S. Cutaneous Leishmaniasis: A Diagnosis of Suspicion. *J. Hand Surg. Eur. Vol.* **2009**, *34*, 555–556, doi:10.1177/1753193408102773.

41. Schnedl, J.; Auer, H.; Fischer, M.; Tomaso, H.; Pustelnik, T.; Mooseder, G. [Cutaneous Leishmaniasis--an Import from Belize]. *Wien. Klin. Wochenschr.* **2007**, *119*, 102–105, doi:10.1007/S00508-007-0871-7.

42. Vinetz, J.M.; Soong, L. Leishmania Mexicana Infection of the Eyelid in a Traveler to Belize. *Braz. J. Infect. Dis.* **2007**, *11*, 149–152, doi:10.1590/S1413-86702007000100030.

43. Clayton, R.; Grabczynska, S. Mucocutaneous Leishmaniasis Presenting as Facial Cellulitis. *J. Laryngol. Otol.* **2005**, *119*, 567–569, doi:10.1258/0022215054352252.

44. Antonovich, D.D.; Callen, J.P.; Paniker, P.U. No Walk in the Park. *Am. J. Med.* **2005**, *118*, 715–716, doi:10.1016/J.AMJMED.2005.04.016.

45. Loo, W.J.; Chan, S.K.; Rytina, E.; Lockwoood, D.N.J.; Sterling, J.C.; Todd, P. Five Cases of Cutaneous Leishmaniasis in Cambridge, U.K. *Br. J. Dermatol.* **2005**, *153*, 1076–1078, doi:10.1111/J.1365-2133.2005.06921.X.

46. Cardenas, G.A.; Gonzalez-Serva, A.; Cohen, C. Multiple Leg Ulcers in a Traveler. *Cleve. Clin. J. Med.* **2004**, *71*, 109–112, doi:10.3949/CCJM.71.2.109.

47. Haider, S.; Boutross-Tadross, O.; Radhi, J.; Momar, N. Cutaneous Ulcer in a Man Returning from Central America. *Can. Med. Assoc.* **2003**, *4*, 591.

48. Tan, H.H.; Wong, S.S.; Ong, B.H. Cutaneous Leishmaniasis: A Report of Two Cases Seen at a Tertiary Dermatological Centre in Singapore. *Singapore Med J* **2000**, *41*, 179–181.

49. Lawn, S.D.; Whetham, J.; Chiodini, P.L.; Kanagalingam, J.; Watson, J.; Behrens, R.H.; Lockwood, D.N.J. New World Mucosal and Cutaneous Leishmaniasis: An Emerging Health Problem among British Travellers. *QJM* **2004**, *97*, 781–788, doi:10.1093/QJMED/HCH127.

50. Bailey, M.S. Cutaneous Leishmaniasis in British Troops Following Jungle Training in Belize. *Travel Med. Infect. Dis.* **2011**, *9*, 253–254, doi:10.1016/J.TMAID.2011.07.001.

51. Scope, A.; Trau, H.; Anders, G.; Barzilai, A.; Confino, Y.; Schwartz, E. Experience with New World Cutaneous Leishmaniasis in Travelers. *J. Am. Acad. Dermatol.* **2003**, *49*, 672–678, doi:10.1067/S0190-9622(03)01576-7.

52. Scope, A.; Trau, H.; Bakon, M.; Yarom, N.; Nasereddin, A.; Schwartz, E. Imported Mucosal Leishmaniasis in a Traveler. *Clin. Infect. Dis.* **2003**, *37*, 83–87, doi:10.1086/377045.

53. Lawn, S.D.; Yardley, V.; Vega-Lopez, F.; Watson, J.; Lockwood, D.N. New World Cutaneous Leishmaniasis in Returned Travellers: Treatment Failures Using Intravenous Sodium Stibogluconate. *Trans. R. Soc. Trop. Med. Hyg.* **2003**, *97*, 443–445, doi:10.1016/S0035-9203(03)90084-8.

54. Mirzabeigi, M.; Farooq, U.; Baraniak, S.; Dowdy, L.; Ciancio, G.; Vincek, V. Reactivation of Dormant Cutaneous Leishmania Infection in a Kidney Transplant Patient. *J. Cutan. Pathol.* **2006**, *33*, 701–704, doi:10.1111/J.1600-0560.2006.00532.X.

55. Solomon, M.; Baum, S.; Barzilai, A.; Scope, A.; Trau, H.; Schwartz, E. Liposomal Amphotericin B in Comparison to Sodium Stibogluconate for Cutaneous Infection Due to Leishmania Braziliensis. *J. Am. Acad. Dermatol.* **2007**, *56*, 612–616, doi:10.1016/j.jaad.2006.06.044.

56. Mings, S.; Beck, J.C.; Davidson, C.; Ondo, A.L.; Shanler, S.D.; Berman, J. Cutaneous Leishmaniasis with Boggy Induration and Simultaneous Mucosal Disease. *Am. J. Trop. Med. Hyg.* **2009**, *80*, 3–5, doi:10.4269/AJTMH.2009.80.3.

57. González, M.; Benito, F.; García, L.; Iglesias, A. [Mucocutaneous Leishmaniasis: An Imported Illness with ENT Repercussions]. *Acta Otorrinolaringol. Esp.* **2009**, *60*, 298–300, doi:10.1016/J.OTORRI.2009.01.006.

58. Jeddi, F.; Caumes, E.; Thellier, M.; Jauréguiberry, S.; Mazier, D.; Buffet, P.A. Drug Hypersensitivity Syndrome Induced by Meglumine Antimoniate. *Am. J. Trop. Med. Hyg.* **2009**, *80*, 939–940, doi:10.4269/AJTMH.2009.80.939.

59. Harms, G.; Scherbaum, H.; Reiter-Owona, I.; Stich, A.; Richter, J. Treatment of Imported New World Cutaneous Leishmaniasis in Germany. *Int. J. Dermatol.* **2011**, *50*, 1336–1342, doi:10.1111/J.1365-4632.2011.04987.X.

60. Di Muccio, T.; Scalone, A.; Bruno, A.; Marangi, M.; Grande, R.; Armignacco, O.; Gradoni, L.; Gramiccia, M. Epidemiology of Imported Leishmaniasis in Italy: Implications for a European Endemic Country. *PLoS One* **2015**, *10*, doi:10.1371/JOURNAL.PONE.0129418.

61. Crovetto-Martínez, R.; Aguirre-Urizar, J.M.; Orte-Aldea, C.; Araluce-Iturbe, I.; Whyte-Orozco, J.; Crovetto-De La Torre, M.A. Mucocutaneous Leishmaniasis Must Be Included in the Differential Diagnosis of Midline Destructive Disease: Two Case Reports. *Oral Surg. Oral Med. Oral Pathol. Oral Radiol.* **2015**, *119*, e20–e26, doi:10.1016/J.OOOO.2014.09.008.

62. Montalvo, A.M.; Fraga, J.; Blanco, O.; González, D.; Monzote, L.; Soong, L.; Capó, V. Imported Leishmaniasis Cases in Cuba (2006-2016): What Have We Learned. *Trop. Dis. Travel Med. vaccines* **2018**, *4*, doi:10.1186/S40794-018-0067-3.

63. Solomon, M.; Sahar, N.; Pavlotzky, F.; Barzilai, A.; Jaffe, C.L.; Nasereddin, A.; Schwartz, E. Mucosal Leishmaniasis in Travelers with Leishmania Braziliensis Complex Returning to Israel. *Emerg. Infect. Dis.* **2019**, *25*, 642–648, doi:10.3201/EID2504.180239.

64. Murray, H.W.; Eiras, D.P.; Kirkman, L.A.; Chai, R.L.; Caplivski, D. Case Report: Mucosal Leishmaniasis in New York City. *Am. J. Trop. Med. Hyg.* **2020**, *102*, 1319–1322, doi:10.4269/AJTMH.19-0861.

65. Guery, R.; Henry, B.; Martin-Blondel, G.; Rouzaud, C.; Cordoliani, F.; Harms, G.; Gangneux, J.P.; Foulet, F.; Bourrat, E.; Baccard, M.; et al. Liposomal Amphotericin B in Travelers with Cutaneous and Muco-Cutaneous Leishmaniasis: Not a Panacea. *PLoS Negl. Trop. Dis.* **2017**, *11*, doi:10.1371/JOURNAL.PNTD.0006094.

66. SC, R.; T, T.; DD, C.; L, V.; H, A.; R, N.; SR, R. American Cutaneous Leishmaniasis in Guyana, South America. *Ann. Trop. Med. Parasitol.* **2001**, *95*, 245–251, doi:10.1080/00034980120053357.

67. Manfredi, R.; Di Bari, M.A.; Calza, L.; Chiodo, F. American Cutaneous Leishmaniasis as a Rare Imported Disease in Europe: A Case Report Favourably Treated with Antimonial Derivatives. *Eur. J. Epidemiol.* **2001**, *17*, 793–795, doi:10.1023/A:1015622016579.

68. Schleucher, R.D.; Zanger, P.; Gaessler, M.; Knobloch, J. Successful Diagnosis and Treatment 50 Years after Exposure: Is Mucocutaneous Leishmaniasis Still a Neglected Differential Diagnosis? *J. Travel Med.* **2008**, *15*, 466–467, doi:10.1111/J.1708-8305.2008.00261.X.

69. Mulvaney, P.; Aram, G.; Maggiore, P.R.; Kutzner, H.; Carlson, J.A. Delay in Diagnosis: Trauma- and Coinfection-Related Cutaneous Leishmaniasis Because of Leishmania Guyanensis Infection. *J. Cutan. Pathol.* **2009**, *36*, 53–60, doi:10.1111/J.1600-0560.2007.00925.X.

70. Solomon, M.; Benenson, S.; Baum, S.; Schwartz, E. Tropical Skin Infections among Israeli Travelers. *Am. J. Trop. Med. Hyg.* **2011**, *85*, 868–872, doi:10.4269/AJTMH.2011.10-0471.

71. Wise, E.S.; Armstrong, M.S.; Watson, J.; Lockwood, D.N. Monitoring Toxicity Associated with Parenteral Sodium Stibogluconate in the Day-Case Management of Returned Travellers with New World Cutaneous Leishmaniasis [Corrected]. *PLoS Negl. Trop. Dis.* **2012**, *6*, doi:10.1371/JOURNAL.PNTD.0001688.

72. Veraldi, S.; Tavecchio, S. Gifts from the Tropics. *Int. J. Dermatol.* **2013**, *52*, 896–897, doi:10.1111/J.1365-4632.2011.05072.X.

73. Neumayr, A.L.C.; Morizot, G.; Visser, L.G.; Lockwood, D.N.J.; Beck, B.R.; Schneider, S.; Bellaud, G.; Cordoliani, F.; Foulet, F.; Laffitte, E.A.; et al. Clinical Aspects and Management of Cutaneous Leishmaniasis in Rheumatoid Patients Treated with TNF-α Antagonists. *Travel Med. Infect. Dis.* **2013**, *11*, 412–420, doi:10.1016/J.TMAID.2013.05.003.

74. Fernández-Arévalo, A.; Ballart, C.; Muñoz-Basagoiti, J.; Basarte, L.; Lobato, G.; Arnau, A.; Abras, A.; Tebar, S.; Llovet, T.; Lami, P.; et al. Autochthonous and Imported Tegumentary Leishmaniasis in Catalonia (Spain): Aetiological Evolution in the Last Four Decades and Usefulness of Different Typing Approaches Based on Biochemical, Molecular and Proteomic Markers. *Transbound. Emerg. Dis.* **2021**, doi:10.1111/TBED.14107.

75. Shin, J.Y.; Lee, Y.B.; Cho, B.K.; Park, H.J. New World Cutaneous Leishmaniasis Treated with Intralesional Injection of Pentavalent Antimony. *Ann. Dermatol.* **2013**, *25*, 80–83, doi:10.5021/AD.2013.25.1.80.

76. Morizot, G.; Kendjo, E.; Mouri, O.; Thellier, M.; Pérignon, A.; Foulet, F.; Cordoliani, F.; Bourrat, E.; Laffitte, E.; Alcaraz, I.; et al. Travelers with Cutaneous Leishmaniasis Cured without Systemic Therapy. *Clin. Infect. Dis.* **2013**, *57*, 370–380, doi:10.1093/CID/CIT269.

77. Ellner, K.; Darie, H.; Goesch, J.; Consigny, P.H. [61-Year-Old Woman with Formation of Nodules after a Tropical Journey]. *Dtsch. Med. Wochenschr.* **2012**, *137*, 2263–2264, doi:10.1055/S-0032-1305166.

78. Calderaro, A.; Montecchini, S.; Rossi, S.; Gorrini, C.; Dell’Anna, M.L.; Piccolo, G.; Medici, M.C.; Arcangeletti, M.C.; Chezzi, C.; De Conto, F. A 22-Year Survey of Leishmaniasis Cases in a Tertiary-Care Hospital in an Endemic Setting. *Int. J. Environ. Res. Public Health* **2014**, *11*, 2834–2845, doi:10.3390/IJERPH110302834.

79. Navarrete-Dechent, C.; Cevallos, C.; Jercic, M.I.; Saldias-Fuentes, C.; González, S.; Labarca, J. [Liposomal Amphotericin B Treatment of Cutaneous Leishmaniasis Caused by L. Braziliensis: An Imported Case Report]. *Rev. Chilena Infectol.* **2018**, *35*, 612–616, doi:10.4067/S0716-10182018000500612.

80. Delgado, O.; Silva, S.; Coraspe, V.; Rivas, M.A.; Rodriguez-Morales, A.J.; Navarro, P.; Franco-Paredes, C. Cutaneous Leishmaniasis Imported from Colombia to Northcentral Venezuela: Implications for Travel Advice. *Travel Med. Infect. Dis.* **2008**, *6*, 376–379, doi:10.1016/J.TMAID.2008.06.012.

81. Sotiropoulos, G.; Wilbur, B. Two Cases of Cutaneous Leishmaniasis Presenting to the Emergency Department as Chronic Ulcers. *J. Emerg. Med.* **2001**, *20*, 353–356, doi:10.1016/S0736-4679(01)00311-0.

82. TF, H.; P, G.; WF, S.; DA, H.; R, M.; DJ, H. Photo Quiz. Lesion on the Arm of a Returning Traveler. Cutaneous Leishmaniasis. *Clin. Infect. Dis.* **2001**, *33*, 815–815, doi:10.1086/322713.

83. ME, W.; LC, L. Photo Quiz. Healthy Student with a Papule. New World Cutaneous Leishmaniasis. *Clin. Infect. Dis.* **2001**, *33*, 816–816, doi:10.1086/322714.

84. Galioto, P.; Fornaro, V. A Case of Mucocutaneous Leishmaniasis. *Ear Nose Throat J* **2002**, *81*, 46–48.

85. Mateo, M.; Cruz, I.; Flores, M.D.; López-Vélez, R. [Slowly Progressing Skin Ulcers Following a Stay in Costa Rica]. *Enferm. Infecc. Microbiol. Clin.* **2005**, *23*, 243–244, doi:10.1157/13073152.

86. Ismailjee, S.B.; Bernstein, J.M.; Burdette, S.D. Bite the Hand That Sprays You. *Skinmed* **2006**, *5*, 296–299, doi:10.1111/J.1540-9740.2006.05028.X.

87. Zaghi, D.; Panosian, C.; Gutierrez, M.A.; Gregson, A.; Taylor, E.; Ochoa, M.T. New World Cutaneous Leishmaniasis: Current Challenges in Diagnosis and Parenteral Treatment. *J. Am. Acad. Dermatol.* **2011**, *64*, 587–592, doi:10.1016/J.JAAD.2009.08.045.

88. Challener, D.; Abu Saleh, O. A Traveler's Unwanted Souvenir. *Am. J. Med.* **2018**, *131*, e137–e138, doi:10.1016/J.AMJMED.2017.09.033.

89. Mann, S.; Phupitakphol, T.; Davis, B.; Newman, S.; Suarez, J.A.; Henao-Martínez, A.; Franco-Paredes, C. Case Report: Cutaneous Leishmaniasis Due to Leishmania (Viannia) Panamensis in Two Travelers Successfully Treated with Miltefosine. *Am. J. Trop. Med. Hyg.* **2020**, *103*, 1081–1084, doi:10.4269/AJTMH.20-0086.

90. Abadir, A.; Patel, A.; Haider, S. Systemic Therapy of New World Cutaneous Leishmaniasis: A Case Report and Review Article. *Can. J. Infect. Dis. Med. Microbiol. = J. Can. des Mal. Infect. la Microbiol. medicale* **2010**, *21*, doi:10.1155/2010/768645.

91. Flores-Figueroa, J.; Okhuysen, P.C.; Von Sonnenburg, F.; Dupont, H.L.; Libman, M.D.; Keystone, J.S.; Hale, D.C.; Burchard, G.; Han, P. V.; Wilder-Smith, A.; et al. Patterns of Illness in Travelers Visiting Mexico and Central America: The GeoSentinel Experience. *Clin. Infect. Dis.* **2011**, *53*, 523–531, doi:10.1093/CID/CIR468.

92. Silgado, A.; Armas, M.; Sánchez-Montalvá, A.; Goterris, L.; Ubals, M.; Temprana-Salvador, J.; Aparicio, G.; Chicharro, C.; Serre-Delcor, N.; Ferrer, B.; et al. Changes in the Microbiological Diagnosis and Epidemiology of Cutaneous Leishmaniasis in Real-Time PCR Era: A Six-Year Experience in a Referral Center in Barcelona. *PLoS Negl. Trop. Dis.* **2021**, *15*, doi:10.1371/JOURNAL.PNTD.0009884.

93. Leitner, V.; Weingast, J.; Harmankaya, K.; Walochnik, J.; Pehamberger, H.; Petzelbauer, P.; Auer, H.; Binder, M. Leishmaniasis in the Tongue of an Immunocompetent Man. *Am. J. Trop. Med. Hyg.* **2010**, *82*, 597–599, doi:10.4269/AJTMH.2010.09-0698.

94. Marovt, M.; Kokol, R.; Stanimirović, A.; Miljković, J. Cutaneous Leishmaniasis: A Case Report - PubMed. *Acta Dermatovenerologica Alpina, Pannonica Adriat.* **2010**, *19*, 41–43.

95. Poeppl, W.; Walochnik, J.; Pustelnik, T.; Auer, H.; Mooseder, G. Cutaneous Leishmaniasis after Travel to Cyprus and Successful Treatment with Miltefosine. *Am. J. Trop. Med. Hyg.* **2011**, *84*, 562–565, doi:10.4269/AJTMH.2011.10-0645.

96. Ondriska, F.; Bukovinova, P.; Votypka, J.; Nohynkova, E.; Boldis, V. Imported New World Cutaneous Leishmaniasis in a Traveller from Slovakia. *Bratisl. Lek. Listy* **2015**, *116*, 203–206, doi:10.4149/BLL_2015_040.

97. Zanger, P.; Kötter, I.; Raible, A.; Gelanew, T.; Schönian, G.; Kremsner, P.G. Case Report: Successful Treatment of Cutaneous Leishmaniasis Caused by Leishmania Aethiopica with Liposomal Amphothericin B in an Immunocompromised Traveler Returning from Eritrea. *Am. J. Trop. Med. Hyg.* **2011**, *84*, 692–694, doi:10.4269/AJTMH.2011.10-0712.

98. Gilkey, T.; Ulman, C.A.; McGwire, B.; Plaza, J.A. Cutaneous Leishmania Aethiopica Diagnosed in the United States. *Dermatol. Online J.* **2021**, *27*, doi:10.5070/D327854705.

99. Vanbrabant, P.; Van Den Broucke, S.; Soentjens, P. Cutaneous Ulcer after a Stay in the Tropics. *Neth. J. Med.* **2015**, *73*, 44.

100. Goodrich, E.S.; Sears, S.C.; Sorrells, T.; Radike, J.K.; Miladi, A.; Glass, J.S. A Case of Cutaneous Leishmaniasis Guyanensis Mimicking Otitis Externa. *Mil. Med.* **2017**, *182*, e1969–e1972, doi:10.7205/MILMED-D-17-00054.

101. Lachaud, L.; Dedet, J.P.; Marty, P.; Faraut, F.; Buffet, P.; Gangneux, J.P.; Ravel, C.; Bastien, P.; France, W.G. for the N. of H.L. in Surveillance of Leishmaniases in France, 1999 to 2012. *Eurosurveillance* **2013**, *18*, 20534, doi:10.2807/1560-7917.ES2013.18.28.20534.

102. Lescure, F.; Bonnard, P.; Chandenier, J.; Schmit, J.; Douadi, Y. [Atypical Cutaneous Leishmaniasis]. *Press. Med* **2002**, *31*, 259–261.

103. Patel, T.A.; Scadding, G.K.; Phillips, D.E.; Lockwood, D.N. Case Report: Old World Mucosal Leishmaniasis: Report of Five Imported Cases to the Hospital for Tropical Diseases, London, United Kingdom. *Am. J. Trop. Med. Hyg.* **2017**, *97*, 1116–1119, doi:10.4269/AJTMH.17-0162.

104. Ehehalt, U.; Schunk, M.; Jensenius, M.; Van Genderen, P.J.J.; Gkrania-Klotsas, E.; Chappuis, F.; Schlagenhauf, P.; Castelli, F.; Lopez-Velez, R.; Parola, P.; et al. Leishmaniasis Acquired by Travellers to Endemic Regions in Europe: A EuroTravNet Multi-Centre Study. *Travel Med. Infect. Dis.* **2014**, *12*, 167–172, doi:10.1016/J.TMAID.2013.12.003.

105. Berens-Riha, N.; Fleischmann, E.; Pratlong, F.; Bretzel, G.; Von Sonnenburg, F.; Löscher, T. Cutaneous Leishmaniasis (Leishmania Tropica) in a German Tourist after Travel to Greece. *J. Travel Med.* **2009**, *16*, 220–222, doi:10.1111/J.1708-8305.2008.00291.X.

106. Bormann, G.; William, T.; Schulz, A.; Marsch, W.; Gaber, G. [American Cutaneous Leishmaniasis: Special Features in Diagnosis and Therapy]. *Dtsch. Med. Wochenschr.* **2003**, *128*, 2065–2068, doi:10.1055/S-2003-42703.

107. Blaylock, J.M.; Wortmann, G.W. A Case Report and Literature Review of "Chiclero's Ulcer." *Travel Med. Infect. Dis.* **2012**, *10*, 275–278, doi:10.1016/J.TMAID.2012.08.005.

108. Guery, R.; Walker, S.L.; Harms, G.; Neumayr, A.; Van Thiel, P.; Gangneux, J.P.; Clerinx, J.; Söbirk, S.K.; Visser, L.; Lachaud, L.; et al. Clinical Diversity and Treatment Results in Tegumentary Leishmaniasis: A European Clinical Report in 459 Patients. *PLoS Negl. Trop. Dis.* **2021**, *15*, e0009863, doi:10.1371/JOURNAL.PNTD.0009863.

109. Basnett, A.; Nguyen, T.A.; Cannavino, C.; Krakowski, A.C. Ablative Fractional Laser Resurfacing with Topical Paromomycin as Adjunctive Treatment for a Recalcitrant Cutaneous Leishmaniasis Wound. *Lasers Surg. Med.* **2015**, *47*, 788–791, doi:10.1002/LSM.22426.

110. Mongkolrattanothai, K.; Nadipuram, S.M.; Krakowski, A.C.; Stone, M.M.; Krogstad, P.; Lehman, D. Leishmaniasis Gone Viral: Social Media and an Outbreak of Cutaneous Leishmaniasis. *Pediatr. Dermatol.* **2016**, *33*, e276–e277, doi:10.1111/PDE.12910.

111. Kuilder, J.S.; Wismans, P.J.; Baerveldt, E.M.; van Hellemond, J.J.; de Mendonça Melo, M.; van Genderen, P.J.J. Leishmania Major Cutaneous Leishmaniasis in 3 Travelers Returning from Israel to the Netherlands. *Emerg. Infect. Dis.* **2016**, *22*, 2022–2024, doi:10.3201/EID2211.161154.

112. Ganjaei, K.G.; Lawton, K.; Gaur, S. Cutaneous Leishmaniasis in an American Adolescent Returning From Israel. *J. Pediatric Infect. Dis. Soc.* **2018**, *7*, E178–E181, doi:10.1093/JPIDS/PIY045.

113. Taxy, J.B.; Goldin, H.M.; Dickie, S.; Cibull, T. Cutaneous Leishmaniasis: Contribution of Routine Histopathology in Unexpected Encounters. *Am. J. Surg. Pathol.* **2019**, *43*, 195–200, doi:10.1097/PAS.0000000000001177.

114. Newlove, T.; Robinson, M.; Meehan, S.A.; Pomerantz, R. Old World Cutaneous Leishmaniasis. *Dermatol. Online J.* **2012**, *18*, 32, doi:10.18295/squmj.9.2021.133.

115. Wollina, U.; Koch, A.; Schönlebe, J.; Tchernev, G.; Chokoeva, A.; Lotti, T. Non-Healing Facial Lesions: Cutaneous Old World Leishmaniasis in Dresden - PubMed. *J Biol Regul Homeost Agents* **2015**, *29*, 99–102.

116. Kelly, P.; Baudry, T.; Peyron, F. Imported Cutaneous Leishmaniasis in a Short-Term Traveler Returning from Central Mali - The Role of PCR. *Travel Med. Infect. Dis.* **2012**, *10*, 97–100, doi:10.1016/J.TMAID.2012.01.004.

117. Spinicci, M.; Zammarchi, L.; Gramiccia, M.; Di Muccio, T.; Bartolozzi, D.; Corsi, P.; Trotta, M.; Bartoloni, A. Effective Meglumine Antimoniate Intralesional Therapy for Chiclero's Ulcer Refractory to Systemic Liposomal Amphotericin B. *J. Travel Med.* **2021**, *28*, 1–2, doi:10.1093/JTM/TAAA169.

118. Eldin, C.; L'Ollivier, C.; Ranque, S.; Gautret, P.; Parola, P. "Chiclero's Ulcer" Due to Leishmania Mexicana in Travelers Returning from Central America: A Case Report and Review of the Literature. *Pathog. (Basel, Switzerland)* **2021**, *10*, doi:10.3390/PATHOGENS10091112.

119. Tsai, P.H.; Chen, Y.T.; Liau, J.Y.; Huang, M.H.; Hsu, H.M.; Yeong, E.K.; Hung, C.C. Molecular Diagnosis and Therapy for Cutaneous Leishmaniasis of a Returned Traveler from Mexico. *J. Microbiol. Immunol. Infect.* **2021**, *54*, 1154–1158, doi:10.1016/J.JMII.2020.07.007.

120. Cohen, J.; Saavedra, A.; Sax, P.; Lipworth, A. Pink Plaque on the Arm of a Man after a Trip to Mexico: Cutaneous Leishmaniasis. *Dermatol. Online J.* **2015**, *21*, 13030.

121. Nasri, J.; Cajacob, L.; Wirz, E.; Ruf, M.T.; Blum, J.; Mühleisen, B.; Navarini, A.A.; Maul, L. V. Pre-Ulcerative Leishmaniasis Mimicking Chilblains in a Returning Traveller from Southern Europe. *J. Eur. Acad. Dermatol. Venereol.* **2021**, *35*, e503–e505, doi:10.1111/JDV.17233.

122. Knöpfel, N.; Noguera-Morel, L.; Azorin, D.; Sanz, F.; Torrelo, A.; Hernández-Martín, A. Cutaneous Leishmania Tropica in Children: Report of Three Imported Cases Successfully Treated with Liposomal Amphotericin B. *J. Eur. Acad. Dermatol. Venereol.* **2018**, *32*, e8–e10, doi:10.1111/JDV.14434.

123. van Hees, C.; van Hellemond, J.; den Boer, M. [A Boy with an Unexpected Souvenir from Morocco]. *Ned. Tijdschr. Geneeskd.* **2014**, *158*, A7606.

124. Merino, P.; González, F.; Herreros, B.; Picazo, J. [Traveler with Multiple Skin Lesions of Six Months' Evolution]. *Enferm. Infecc. Microbiol. Clin.* **2010**, *28*, 323–324, doi:10.1016/J.EIMC.2009.06.014.

125. Stewardson, A.J.; Leder, K.; Torresi, J.; Johnson, D.F. Two Cases of Old World Cutaneous Leishmaniasis in Australian Travelers Visiting Morocco. *J. Travel Med.* **2010**, *17*, 278–280, doi:10.1111/J.1708-8305.2010.00417.X.

126. EM, T.; JR, M.; A, V. Photo Quiz: A 24-Year-Old Traveler With an Insect Bite and Rash. *Clin. Infect. Dis.* **2015**, *61*, 1314, doi:10.1093/CID/CIV359.

127. Crogan, J.; Gunasekera, H.; Wood, N.; Sheikh, M.; Isaacs, D. Management of Old World Cutaneous Leishmaniasis in Refugee Children. *Pediatr. Infect. Dis. J.* **2010**, *29*, 357–359, doi:10.1097/INF.0B013E3181C2A1FD.

128. Kitano, H.; Sanjoba, C.; Goto, Y.; Iwamoto, K.; Kitagawa, H.; Nomura, T.; Omori, K.; Shigemoto, N.; Hide, M.; Matsumoto, Y.; et al. Complicated Cutaneous Leishmaniasis Caused by an Imported Case of Leishmania Tropica in Japan: A Case Report. *Trop. Med. Health* **2021**, *49*, doi:10.1186/S41182-021-00312-4.

129. Royer, M.; Crowe, M. American Cutaneous Leishmaniasis: A Cluster of 3 Cases during Military Training in Panama. *Arch. Pathol. Lab. Med.* **2002**, *126*, 471.

130. Barry, M.A.; Koshelev, M. V.; Sun, G.S.; Grekin, S.J.; Stager, C.E.; Diwan, A.H.; Wasko, C.A.; Murray, K.O.; Woc-Colburn, L. Cutaneous Leishmaniasis in Cuban Immigrants to Texas Who Traveled through the Darién Jungle, Panama. *Am. J. Trop. Med. Hyg.* **2014**, *91*, 345–347, doi:10.4269/AJTMH.14-0124.

131. Alcântara de Castro, E.A.; Thomaz Soccol, V.; Membrive, N.; Luz, E. Epidemiological and Clinical Study of 332 Cases of Cutaneous Leishmaniasis in the North of Parana State from 1993 to 1998. *Rev. Soc. Bras. Med. Trop.* **2002**, *35*, 445–452, doi:10.1590/S0037-86822002000500004.

132. Eichner, S.; Thoma-Uszynski, S.; Herrgott, I.; Sebald, H.; Debus, A.; Tsianakas, A.; Ehrchen, J.; Harms, G.; Simon, M.; Sunderkötter, C.; et al. Clinical Complexity of Leishmania (Viannia) Braziliensis Infections amongst Travelers. *Eur. J. Dermatol.* **2013**, *23*, 218–223, doi:10.1684/EJD.2013.1934.

133. Monteiro, W.M.; Neitzke-Abreu, H.C.; Ferreira, M.E.M.D.C.; De Melo, G.C.; Barbosa, M.D.G.V.; Lonardoni, M.V.C.; Silveira, T.G.V.; Teodoro, U. [Population Mobility and Production of American Tegumentary Leishmaniasis in the State of Paraná, Southern Brazil]. *Rev. Soc. Bras. Med. Trop.* **2009**, *42*, 509–514, doi:10.1590/S0037-86822009000500007.

134. Brecelj, M.; Pikelj, F.; Gubenšek, F.; Anderluh, G. Polymerase Chain Reaction as a Diagnostic Tool for Detecting Leishmania. *Infection* **2000**, *28*, 111–113, doi:10.1007/S150100050057.

135. Lange, C.G.; Salata, R.A. [Cutaneous Leishmaniasis]. *Med. Klin. (Munich).* **2001**, *96*, 561–562, doi:10.1007/PL00002242.

136. Schönian, G.; Nasereddin, A.; Dinse, N.; Schweynoch, C.; Schallig, H.D.F.H.; Presber, W.; Jaffe, C.L. PCR Diagnosis and Characterization of Leishmania in Local and Imported Clinical Samples. *Diagn. Microbiol. Infect. Dis.* **2003**, *47*, 349–358, doi:10.1016/S0732-8893(03)00093-2.

137. Costa, J.W.; Milner, D.A.; Maguire, J.H. Mucocutaneous Leishmaniasis in a US Citizen. *Oral Surg. Oral Med. Oral Pathol. Oral Radiol. Endod.* **2003**, *96*, 573–577, doi:10.1016/S1079-2104(03)00299-3.

138. Clegg, A.; Hall, C.; Lockwood, D. An Ulcer That Will Not Heal. *BMJ* **2018**, *362*, doi:10.1136/BMJ.K3042.

139. Crone, C.G.; Helleberg, M. Cutaneous Leishmaniasis with Secondary Mucosal Disease in a Traveller Due to Leishmania (Viannia) Braziliensis. *J. Travel Med.* **2020**, *27*, doi:10.1093/JTM/TAZ093.

140. Raghunath, R.S.; Yong, A.S.W.; Igali, L.; Tan, E.; Lockwood, D. Cutaneous Leishmaniasis in a Returning UK Traveller. *Postgrad. Med. J.* **2014**, *90*, 540–541, doi:10.1136/POSTGRADMEDJ-2014-132620.

141. Michelerio, A.; Barruscotti, S.; Bossi, G.; Brazzelli, V. Pediatric Old World Cutaneous Leishmaniasis Treated with Oral Fluconazole: A Case Series. *Pediatr. Dermatol.* **2018**, *35*, 384–387, doi:10.1111/PDE.13493.

142. Raschke, A.; Wetzig, T.; Paasch, U.; Grunewald, S.; Simon, J.C. [Firm Blue-Red Solid Nodules on the Forehead of a 39-Year-Old Patient]. *J. Dtsch. Dermatol. Ges.* **2005**, *3*, 919–920, doi:10.1111/J.1610-0387.2005.05075.X.

143. Watson, I.T.; Patel, R.C.; Jahan-Tigh, R.R. Extensive, Multifocal Cutaneous Leishmaniasis Presenting with Varied Morphologies in an Immunosuppressed Patient. *Am. J. Trop. Med. Hyg.* **2019**, *101*, 281–282, doi:10.4269/AJTMH.18-0998.

144. Ahmed, Z.; Chowdhury, S.; Bhuiyan, S. Cutaneous Leishmaniasis | Mymensingh Medical Journal. *Mymensingh Med. J.* **2009**, *18*, 260–263.

145. Mazumder, S.A.; Pandey, S.; Brewer, S.C.; Baselski, V.S.; Weina, P.J.; Land, M.A.; Fleckenstein, J.M. Lingual Leishmaniasis Complicating Visceral Disease. *J. Travel Med.* **2010**, *17*, 212–214, doi:10.1111/J.1708-8305.2010.00403.X.

146. Rahman, H.; Razzak, M.; Chanda, B.; Bhaskar, K.; Mondal, D. Cutaneous Leishmaniasis in an Immigrant Saudi Worker: A Case Report. *J. Heal. Popul. Nutr.* **2014**, *32*, 372–376.

147. Zhang, M.; Liu, F.; Liu, H.B.; Hu, W.X.; Sang, H. Imported Cutaneous Leishmaniasis Caused by Leishmania Major in a Chinese Laborer Who Worked in Saudi Arabia. *An. Bras. Dermatol.* **2016**, *91*, 365–367, doi:10.1590/ABD1806-4841.20163820.

148. Basher, A.; Nath, P.; Dey, T.; Sayeed, A.A.; Faiz, M.A.; Chowdhury, F.R. Cutaneous Leishmaniasis in Immigrant Workers Returning to Bangladesh - An Emerging Problem. *Travel Med. Infect. Dis.* **2017**, *19*, 62–63, doi:10.1016/J.TMAID.2017.05.013.

149. Khan, M.A.A.; Chowdhury, R.; Nath, R.; Hansen, S.; Nath, P.; Maruf, S.; Abd El Wahed, A.; Mondal, D. Imported Cutaneous Leishmaniasis: Molecular Investigation Unveils Leishmania Major in Bangladesh. *Parasit. Vectors* **2019**, *12*, doi:10.1186/S13071-019-3771-6.

150. Madke, B.; Kharkar, V.; Chikhalkar, S.; Mahajan, S.; Khopkar, U. Successful Treatment of Multifocal Cutaneous Leishmaniasis with Miltefosine. *Indian J. Dermatol.* **2011**, *56*, 587–590, doi:10.4103/0019-5154.87165.

151. González-Llavona, B.; Biosca-Echenique, G.; Soto-Díaz, A.; Naranjo-Díaz, M.J.; Espadafor-López, B.; García-Mellado, V. [Cutaneous Leishmaniasis in a Senegal Patient]. *Actas Dermosifiliogr.* **2007**, *98*, 54–58, doi:10.1016/S1578-2190(07)70392-X.

152. Loo, W.J.; Lishman, S.C.; Lockwood, D.N.J.; Todd, P.M. Case 2. Cutaneous Leishmaniasis, Leishmania Amastigotes. *Clin. Exp. Dermatol.* **2003**, *28*, 567–568, doi:10.1046/J.1365-2230.2003.01360.X.

153. Darné, S.; Sinclair, S.A. A Sandfly in Surrey? A Case of Cutaneous Leishmaniasis in the United Kingdom without History of Recent Travel to an Endemic Area. *Clin. Exp. Dermatol.* **2006**, *31*, 155–156, doi:10.1111/J.1365-2230.2005.01984.X.

154. Terhorst, D.; Blume-Peytavi, U.; Schönian, G.; Schewe, C.; Haas, N.; Burbach, G.J. Leishmaniasis: A Reminder in the Face of Forgotten Travel. *J. Pediatr.* **2012**, *161*, doi:10.1016/J.JPEDS.2012.05.013.

155. Ehlert, N.; Seilmaier, M.; Guggemos, W.; Löscher, T.; Meurer, A.; Wendtner, C.M. [Severe Oral Mucositis in a Patient with HIV Infection]. *Dtsch. Med. Wochenschr.* **2013**, *138*, 1601–1605, doi:10.1055/S-0033-1343296.

156. Siah, T.; Lavender, T.; Charlton, F.; Wahie, S.; Schwab, U. An Unusual Erysipelas-like Presentation. *Dermatol Online J.* **2014**, *20*, 21255.

157. Roberts, R.M.; Mukherjee, J.; Phillips, D. Laryngeal Leishmaniasis in a Patient Taking Inhaled Corticosteroids. *BMJ Case Rep.* **2016**, *2016*, doi:10.1136/BCR-2016-215444.

158. Menten, K.; Soentjens, P.; Caenepeel, P.; Lemkens, P. Mucocutaneous Leishmaniasis of the Nose: A Case Report - PubMed. *B-ENT* **2015**, *11*, 77–80.

159. Suqati, A.A.; Pudszuhn, A.; Hofmann, V.M. Mucocutaneous Leishmaniasis: Case Report and Literature Review of a Rare Endonasal Infection. *Pan Afr. Med. J.* **2020**, *36*, 1–4, doi:10.11604/PAMJ.2020.36.292.24543.

160. Schwartz, T.; Jensenius, M.; Blomberg, B.; Fladeby, C.; Mæland, A.; Pettersen, F.O. Imported Visceral Leishmaniasis and Immunosuppression in Seven Norwegian Patients. *Trop. Dis. Travel Med. vaccines* **2019**, *5*, doi:10.1186/S40794-019-0092-X.

161. Santangeli, L.; McCluney, N.A.; Hathorn, I.; Shakeel, M.; Anderson, C. Leishmaniasis Presenting to the Otolaryngologist: A Rare but Important Cause of Persistent Hoarseness. *J. Laryngol. Otol.* **2009**, *123*, 1181–1183, doi:10.1017/S0022215109004216.

162. Teemul, T.A.; Giles-Lima, M.; Williams, J.; Lester, S.E. Laryngeal Leishmaniasis: Case Report of a Rare Infection. *Head Neck* **2013**, *35*, doi:10.1002/HED.23141.

163. Ito, K.; Takahara, M.; Ito, M.; Oshiro, M.; Takahashi, K.; Uezato, H.; Imafuku, S. An Imported Case of Cutaneous Leishmaniasis Caused by Leishmania (Leishmania) Donovani in Japan. *J. Dermatol.* **2014**, *41*, 926–928, doi:10.1111/1346-8138.12609.

164. Van Der Snoek, E.M.; Lammers, A.M.; Kortbeek, L.M.; Roelfsema, J.H.; Bart, A.; Jaspers, C.A.J.J. Spontaneous Cure of American Cutaneous Leishmaniasis Due to Leishmania Naiffi in Two Dutch Infantry Soldiers. *Clin. Exp. Dermatol.* **2009**, *34*, doi:10.1111/J.1365-2230.2009.03658.X.

165. Saroufim, M.; Charafeddine, K.; Issa, G.; Khalifeh, H.; Habib, R.H.; Berry, A.; Ghosn, N.; Rady, A.; Khalifeh, I. Ongoing Epidemic of Cutaneous Leishmaniasis among Syrian Refugees, Lebanon. *Emerg. Infect. Dis.* **2014**, *20*, 1712–1715, doi:10.3201/EID2010.140288.

166. Bradshaw, S.; Litvinov, I. V. Dermal Leishmaniasis in a 25-Year-Old Syrian Refugee. *CMAJ* **2017**, *189*, E1397, doi:10.1503/CMAJ.170844.

167. Hijawi, K.J.F.; Hijjawi, N.S.; Ibbini, J.H. Detection, Genotyping, and Phylogenetic Analysis of Leishmania Isolates Collected from Infected Jordanian Residents and Syrian Refugees Who Suffered from Cutaneous Leishmaniasis. *Parasitol. Res.* **2019**, *118*, 793–805, doi:10.1007/S00436-019-06222-Z.

168. Van Kesteren, L.; Maniewski, U.; Bottieau, E.; Cnops, L.; Huits, R. Cutaneous Leishmaniasis in Syrian Refugee Children: A Case Series. *Pediatr. Infect. Dis. J.* **2020**, *39*, E154–E156, doi:10.1097/INF.0000000000002676.

169. Talas, J.; Mielcarek, K.; Wu, J.; Brunner, M.; Steinhoff, M.; Zouboulis, C.C. [Cutaneous Leishmaniasis in Germany-Still a Travel-Related Disease]. *Hautarzt.* **2022**, *73*, 146–151, doi:10.1007/S00105-021-04890-6.

170. Çizmeci, Z.; Karakuş, M.; Karabela, Ş.N.; Erdoğan, B.; Güleç, N. Leishmaniasis in Istanbul; A New Epidemiological Data about Refugee Leishmaniasis. *Acta Trop.* **2019**, *195*, 23–27, doi:10.1016/J.ACTATROPICA.2019.04.008.

171. Bizri, N.A.; Alam, W.; Khoury, M.; Musharrafieh, U.; Ghosn, N.; Berri, A.; Bizri, A.R. The Association Between the Syrian Crisis and Cutaneous Leishmaniasis in Lebanon. *Acta Parasitol.* **2021**, *66*, 1240–1245, doi:10.1007/S11686-021-00395-3.

172. Zhao, Y.L.; Li, S.H.; Zhu, X.; Zhou, R.M.; Yang, C.Y.; Liu, Y.; Niu, Y.N.; Lu, D.L.; Zhang, H.W.; Zhao, D.Y. Investigating the Aggregation of Imported Cutaneous Leishmaniasis in Henan, Central China. *Biomed. Environ. Sci.* **2021**, *34*, 247–249, doi:10.3967/BES2021.032.

173. Montalvo, A.M.; De Armas, Y.; Fraga, J.; Blanco, O.; Menéndez, R.; Montoto, V.; Capó de Paz, V. Molecular and Histological Tools to Diagnose an Imported Case of American Cutaneous Leishmaniasis in Cuba. *Int. J. Dermatol.* **2015**, *54*, 1175–1179, doi:10.1111/IJD.12915.

174. Downing, C.P.; Woc-Colburn, L.; Tyring, S.K. Nasal Erythema and Crusting after a Trip to the Venezuelan Rainforest. *JAMA* **2014**, *312*, 1250–1251, doi:10.1001/JAMA.2014.3610.
