# Supplementary material for: Non-Endemic Leishmaniases Reported Globally in Humans between 2000 and 2021—A Comprehensive Review
Source: Pathogens. 2022 Aug 16;11(8):921. doi: 10.3390/pathogens11080921 (PMC9415673; doi:10.3390/pathogens11080921)
Supplement: Supplementary file 1 [file pathogens-11-00921-s001.zip › Supplementary-Table S3.pdf]

Supplementary Table S3. References of identifications of species/complex by country in the Old and New Worlds.

	Species/Complex									
**Country**	***L. aethiopica***	***L. braziliensis* complex**	***L. guyanensis* complex**	***L. donovani* complex**	***L. lainsoni***	***L. major***	***L. mexicana* complex**	***L. naiffi***	***L. tropica***	***L. (Viannia) sp.***
Afghanistan				(1)		(1–5)			(1–3,5–12)	
Algeria				(6,7,13,14)		(6,14–16)				
Argentina		(17)								(6)
Belize		(4,8,18–23)					(1,21,24–26)			(1,6,27)
Bolivia		(6,8,28–37)	(28,38)				(1)			(1,6,27,39)
Brazil		(6,8,28,37,40–42)	(6,43)	(14)	(6)					
Burkina Faso						(7,28,44)			(7)	
Chad						(28,44,45)				
Chile		(46)								
Colombia		(5,28,47)								(39)
Costa Rica		(5,6,32,37,48,49)	(6,15,50–56)				(47)			(6)
Croatia				(57)						
Cyprus				(6,58)						
Ecuador		(6,8,28,32,37,40)	(40,53,59)							(2,6)
Egypt	(14)			(6,14)					(6,8)	
Eritrea	(60)									
Ethiopia	(2,15,28,61)								(6)	
France				(6,8,28)						
French Guiana		(5,6,8,15,29,32,37)	(6,15,62,63)				(6)	(6,15)		
Greece				(3,6,28)					(19,64)	
Guatemala		(40,65)					(8,28,66)			
Guyana										(67)
Iran						(6)			(5,6)	
Iraq						(1,5,28,68)				
Israel						(7,69–72)			(28,40,69)	
Italy				(5,6,8,14,73)						
Jordan						(40)			(19,28)	
Lybia				(6)		(28)				

Mali				(6,74)			(6)	
Malta			(5,6,8)					
Mauritania				(6,7,40)				
Mexico	(28)				(5,6,28,75–78)			(6)
Montenegro			(2)					
Morocco			(6,79)	(6,7,15,28,32,38,40,45,80)			(6,28,81)	
Nicaragua		(82)						
Pakistan			(1,6)	(1)			(1,3,6,83)	
Panama		(40,84,85)						
Paraguay	(86)							
Peru	(5,6,8,28,37,69,86–90)	(6,15,53)			(5)			(1,2,91,92)
Tunisia			(6,7,38)	(2,6,7,15,28,38,45,93)			(6,28)	
Turkey			(6,67)				(6)	
Turkmenistan				(44,94)				
Saudi Arabia				(16,95,96)			(97)	
Senegal				(6)				
Spain			(5,6,6,13,14,22,98–104)					
Sri Lanka			(105)					
Sudan				(6,28)				
Suriname		(6)				(106)		
Syria				(5,28,107,108)			(2,6,28,93,107–112)	
Venezuela	(111,113)							
Yemen				(28)				

References

1. Wall, E.C.; Watson, J.; Armstrong, M.; Chiodini, P.L.; Lockwood, D.N. Epidemiology of Imported Cutaneous Leishmaniasis at the Hospital for Tropical Diseases, London, United Kingdom: Use of Polymerase Chain Reaction to Identify the Species. *Am J Trop Med Hyg* **2012**, *86*, 115–118, doi:10.4269/AJTMH.2012.10-0558.
2. Söbirk, S.K.; Inghammar, M.; Collin, M.; Davidsson, L. Imported Leishmaniasis in Sweden 1993-2016. *Epidemiol Infect* **2018**, *146*, 1267–1274, doi:10.1017/S0950268818001309.
3. Stark, D.; van Hal, S.; Lee, R.; Marriott, D.; Harkness, J. Leishmaniasis, an Emerging Imported Infection: Report of 20 Cases from Australia. *J Travel Med* **2008**, *15*, 351–354, doi:10.1111/J.1708-8305.2008.00223.X.
4. van Thiel, P.P.A.M.; Leenstra, T.; Kager, P.A.; de Vries, H.J.; van Vugt, M.; van der Meide, W.F.; Bart, A.; Zeegelaar, J.E.; van der Sluis, A.; Schallig, H.D.F.H.; et al. Miltefosine Treatment of *Leishmania major* Infection: An Observational Study Involving Dutch Military Personnel Returning from Northern Afghanistan. *Clin Infect Dis* **2010**, *50*, 80–83, doi:10.1086/648726.
5. Roberts, T.; Barratt, J.; Sandaradura, I.; Lee, R.; Harkness, J.; Marriott, D.; Ellis, J.; Stark, D. Molecular Epidemiology of Imported Cases of Leishmaniasis in Australia from 2008 to 2014. *PLoS One* **2015**, *10*, e0119212, doi:10.1371/JOURNAL.PONE.0119212.
6. Guery, R.; Walker, S.L.; Harms, G.; Neumayr, A.; van Thiel, P.; Gangneux, J.P.; Clerinx, J.; Söbirk, S.K.; Visser, L.; Lachaud, L.; et al. Clinical Diversity and Treatment Results in Tegumentary Leishmaniasis: A European Clinical Report in 459 Patients. *PLoS Negl Trop Dis* **2021**, *15*, e0009863, doi:10.1371/JOURNAL.PNTD.0009863.
7. Morizot, G.; Delgiudice, P.; Caumes, E.; Laffitte, E.; Marty, P.; Dupuy, A.; Sarfati, C.; Hadj-Rabia, S.; Darie, H.; le Guern, A.S.; et al. Healing Of Old World Cutaneous Leishmaniasis In Travelers Treated With Fluconazole: Drug Effect Or Spontaneous Evolution? *The American Journal of Tropical Medicine and Hygiene* **2007**, *76*, 48–52, doi:10.4269/AJTMH.2007.76.48.
8. Harms, G.; Schönian, G.; Feldmeier, H. Leishmaniasis in Germany. *Emerg Infect Dis* **2003**, *9*, 872-875, doi:10.3201/EID0907.030023.
9. Flaig, M.J.; Rupec, J.; Ruzicka, T.; Rupec, R.A. [Topical Treatment of Persistent Cutaneous Leishmaniasis with Paromomycin]. *Hautarzt* **2007**, *58*, 689–692, doi:10.1007/S00105-007-1297-X.
10. Fathi, R.; Fathi, A. A Case of Cutaneous Leishmaniasis Found in Indiana. *Dermatol Online Journal* **2013**, *19*, 18982.
11. Ntais, P.; Christodoulou, V.; Tsirigotakis, N.; Dokianakis, E.; Dedet, J.P.; Pratlong, F.; Antoniou, M. Will the Introduction of *Leishmania tropica* MON-58, in the Island of Crete, Lead to the Settlement and Spread of This Rare Zymodeme? *Acta Trop* **2014**, *132*, 125–130, doi:10.1016/J.ACTATROPICA.2014.01.003.
12. Islam, S. Rapidly Progressing Facial Leishmaniasis: Effective Treatment with Liposomal Amphotericin B and a Review of the Management of Old World Cutaneous Leishmaniasis. *Paediatr Int Child Health* **2018**, *38*, 158–161, doi:10.1080/20469047.2017.1296216.
13. Patel, T.A.; Scadding, G.K.; Phillips, D.E.; Lockwood, D.N. Case Report: Old World Mucosal Leishmaniasis: Report of Five Imported Cases to the Hospital for Tropical Diseases, London, United Kingdom. *Am J Trop Med Hyg* **2017**, *97*, 1116–1119, doi:10.4269/AJTMH.17-0162.
14. Neumayr, A.L.C.; Morizot, G.; Visser, L.G.; Lockwood, D.N.J.; Beck, B.R.; Schneider, S.; Bellaud, G.; Cordoliani, F.; Foulet, F.; Laffitte, E.A.; et al. Clinical Aspects and Management of Cutaneous Leishmaniasis in Rheumatoid Patients Treated with TNF-α Antagonists. *Travel Med Infect Dis* **2013**, *11*, 412–420, doi:10.1016/J.TMAID.2013.05.003.
15. Lavergne, R.A.; Iriart, X.; Martin-Blondel, G.; Chauvin, P.; Menard, S.; Fillaux, J.; Cassaing, S.; Roques-Malecaze, C.; Arnaud, S.; Valentin, A.; et al. Contribution of Molecular Diagnosis to the Management of Cutaneous Leishmaniasis in Travellers. *Clin Microbiol Infect* **2014**, *20*, doi:10.1111/1469-0691.12487.

16. Yang, Y.-T.; Zhang, M.; Gao, C.-H.; Shi, F.; Guan, L.-R.; Wang, J.-Y. [Detection and Species Identification of Two Imported Cases of Cutaneous Leishmaniasis]. *Zhongguo Ji Sheng Chong Xue Yu Ji Sheng Chong Bing Za Zhi* **2011**, *29*, 461–464.

17. Basile, G.; Cristofaro, G.; Locatello, L.G.; Vellere, I.; Piccica, M.; Bresci, S.; Maggiore, G.; Gallo, O.; Novelli, A.; di Muccio, T.; et al. Refractory Mucocutaneous Leishmaniasis Resolved with Combination Treatment Based on Intravenous Pentamidine, Oral Azole, Aerosolized Liposomal Amphotericin B, and Intralesional Meglumine Antimoniate. *Int J Infect Dis* **2020**, *97*, 204–207, doi:10.1016/J.IJID.2020.06.003.

18. Haider, S.; Boutross-Tadross, O.; Radhi, J.; Momar, N. Cutaneous Ulcer in a Man Returning from Central America. *CMAJ* **2003**, *4*, 591.

19. Loo, W.J.; Lishman, S.C.; Lockwood, D.N.J.; Todd, P.M. Case 2. Cutaneous Leishmaniasis, *Leishmania* Amastigotes. *Clin Exp Dermatol* **2003**, *28*, 567–568, doi:10.1046/J.1365-2230.2003.01360.X.

20. Clayton, R.; Grabczynska, S. Mucocutaneous Leishmaniasis Presenting as Facial Cellulitis. *J Laryngol Otol* **2005**, *119*, 567–569, doi:10.1258/0022215054352252.

21. Bailey, M.S. Cutaneous Leishmaniasis in British Troops Following Jungle Training in Belize. *Travel Med Infect Dis* **2011**, *9*, 253–254, doi:10.1016/J.TMAID.2011.07.001.

22. van der Snoek, E.M.; Couwenberg, S.M.; Stijnis, C.; Kortbeek, L.M.; Schadd, E.M. Two Cases of Cutaneous Leishmaniasis in Dutch Military Personnel Treated with Oral Miltefosine. *J R Army Med Corps* **2017**, *163*, 68–70, doi:10.1136/JRAMC-2015-000530.

23. Holmes, W.J.M.; Tehrani, H.; Liew, S. Cutaneous Leishmaniasis: A Diagnosis of Suspicion. *J Hand Surg Eur Vol* **2009**, *34*, 555–556, doi:10.1177/1753193408102773.

24. Demers, E.; Forrest, D.M.; Weichert, G.E. Cutaneous Leishmaniasis in a Returning Traveller. *CMAJ* **2013**, *185*, 681–683, doi:10.1503/CMAJ.120694.

25. Bailey, M.S.; Langman, G. Misdiagnosis of Cutaneous Leishmaniasis and Recurrence after Surgical Excision. *J R Army Med Corps* **2014**, *160*, 314–316, doi:10.1136/JRAMC-2013-000123.

26. Vinetz, J.M.; Soong, L. *Leishmania mexicana* Infection of the Eyelid in a Traveler to Belize. *Braz J Infect Dis* **2007**, *11*, 149–152, doi:10.1590/S1413-86702007000100030.

27. Lawn, S.D.; Yardley, V.; Vega-Lopez, F.; Watson, J.; Lockwood, D.N. New World Cutaneous Leishmaniasis in Returned Travellers: Treatment Failures Using Intravenous Sodium Stibogluconate. *Trans R Soc Trop Med Hyg* **2003**, *97*, 443–445, doi:10.1016/S0035-9203(03)90084-8.

28. di Muccio, T.; Scalone, A.; Bruno, A.; Marangi, M.; Grande, R.; Armignacco, O.; Gradoni, L.; Gramiccia, M. Epidemiology of Imported Leishmaniasis in Italy: Implications for a European Endemic Country. *PLoS One* **2015**, *10*, e0129418, doi:10.1371/JOURNAL.PONE.0129418.

29. Scope, A.; Trau, H.; Anders, G.; Barzilai, A.; Confino, Y.; Schwartz, E. Experience with New World Cutaneous Leishmaniasis in Travelers. *J Am Acad Dermatol* **2003**, *49*, 672–678, doi:10.1067/S0190-9622(03)01576-7.

30. Scope, A.; Trau, H.; Bakon, M.; Yarom, N.; Nasereddin, A.; Schwartz, E. Imported Mucosal Leishmaniasis in a Traveler. *Clin Infect Dis* **2003**, *37*, 83–87, doi:10.1086/377045.

31. Solomon, M.; Baum, S.; Barzilai, A.; Scope, A.; Trau, H.; Schwartz, E. Liposomal Amphotericin B in Comparison to Sodium Stibogluconate for Cutaneous Infection Due to *Leishmania braziliensis*. *J Am Acad Dermatol* **2007**, *56*, 612–616, doi:10.1016/j.jaad.2006.06.044.

32. Pérez-Ayala, A.; Norman, F.; Pérez-Molina, J.A.; Herrero, J.M.; Monge, B.; López-Vélez, R. Imported Leishmaniasis: A Heterogeneous Group of Diseases. *J Travel Med* **2009**, *16*, 395–401, doi:10.1111/J.1708-8305.2009.00341.X.

33. Mings, S.; Beck, J.C.; Davidson, C.; Ondo, A.L.; Shanler, S.D.; Berman, J. Cutaneous Leishmaniasis with Boggy Induration and Simultaneous Mucosal Disease. *Am J Trop Med Hyg* **2009**, *80*, 3–5, doi:10.4269/AJTMH.2009.80.3.

34. González, M.; Benito, F.; García, L.; Iglesias, A. [Mucocutaneous Leishmaniasis: An Imported Illness with ENT Repercussions]. *Acta Otorrinolaringol Esp* **2009**, *60*, 298–300, doi:10.1016/J.OTORRI.2009.01.006.

35. Solomon, M.; Sahar, N.; Pavlotzky, F.; Barzilai, A.; Jaffe, C.L.; Nasereddin, A.; Schwartz, E. Mucosal Leishmaniasis in Travelers with *Leishmania braziliensis* Complex Returning to Israel. *Emerg Infect Dis* **2019**, *25*, 642–648, doi:10.3201/EID2504.180239.

36. Murray, H.W.; Eiras, D.P.; Kirkman, L.A.; Chai, R.L.; Caplivski, D. Case Report: Mucosal Leishmaniasis in New York City. *Am J Trop Med Hyg* **2020**, *102*, 1319–1322, doi:10.4269/AJTMH.19-0861.

37. Harms, G.; Scherbaum, H.; Reiter-Owona, I.; Stich, A.; Richter, J. Treatment of Imported New World Cutaneous Leishmaniasis in Germany. *Int J Dermatol* **2011**, *50*, 1336–1342, doi:10.1111/J.1365-4632.2011.04987.X.

38. Poeppl, W.; Oeser, C.; Grabmeier-Pfistershammer, K.; Walochnik, J.; Burgmann, H. Clinical Findings and Management of Imported Cutaneous Leishmaniasis: Report of 14 Cases from Austria. *Travel Med Infect Dis* **2013**, *11*, 90–94, doi:10.1016/J.TMAID.2013.03.002.

39. Lawn, S.D.; Whetham, J.; Chiodini, P.L.; Kanagalingam, J.; Watson, J.; Behrens, R.H.; Lockwood, D.N.J. New World Mucosal and Cutaneous Leishmaniasis: An Emerging Health Problem among British Travellers. *QJM* **2004**, *97*, 781–788, doi:10.1093/QJMED/HCH127.

40. Fernández-Arévalo, A.; Ballart, C.; Muñoz-Basagoiti, J.; Basarte, L.; Lobato, G.; Arnau, A.; Abras, A.; Tebar, S.; Llovet, T.; Lami, P.; et al. Autochthonous and Imported Tegumentary Leishmaniasis in Catalonia (Spain): Aetiological Evolution in the Last Four Decades and Usefulness of Different Typing Approaches Based on Biochemical, Molecular and Proteomic Markers. *Transbound Emerg Dis* **2021**, doi:10.1111/TBED.14107.

41. Schleucher, R.D.; Zanger, P.; Gaessler, M.; Knobloch, J. Successful Diagnosis and Treatment 50 Years after Exposure: Is Mucocutaneous Leishmaniasis Still a Neglected Differential Diagnosis? *J Travel Med* **2008**, *15*, 466–467, doi:10.1111/J.1708-8305.2008.00261.X.

42. Manfredi, R.; di Bari, M.A.; Calza, L.; Chiodo, F. American Cutaneous Leishmaniasis as a Rare Imported Disease in Europe: A Case Report Favourably Treated with Antimonial Derivatives. *Eur J Epidemiol* **2001**, *17*, 793–795, doi:10.1023/A:1015622016579.

43. Mulvaney, P.; Aram, G.; Maggiore, P.R.; Kutzner, H.; Carlson, J.A. Delay in Diagnosis: Trauma- and Coinfection-Related Cutaneous Leishmaniasis Because of *Leishmania guyanensis* Infection. *J Cutan Pathol* **2009**, *36*, 53–60, doi:10.1111/J.1600-0560.2007.00925.X.

44. Kuna, A.; Gajewski, M.; Bykowska, M.; Pietkiewicz, H.; Olszański, R.; Myjak, P. Imported Cutaneous Leishmaniasis: A 13-Year Experience of a Polish Tertiary Center. *Postepy Dermatol Alergol* **2019**, *36*, 104–111, doi:10.5114/ADA.2019.82830.

45. Calderaro, A.; Montecchini, S.; Rossi, S.; Gorrini, C.; Dell'Anna, M.L.; Piccolo, G.; Medici, M.C.; Arcangeletti, M.C.; Chezzi, C.; de Conto, F. A 22-Year Survey of Leishmaniasis Cases in a Tertiary-Care Hospital in an Endemic Setting. *Int J Environ Res Public Health* **2014**, *11*, 2834–2845, doi:10.3390/IJERPH110302834.

46. Navarrete-Dechent, C.; Cevallos, C.; Jercic, M.I.; Saldias-Fuentes, C.; González, S.; Labarca, J. [Liposomal Amphotericin B Treatment of Cutaneous Leishmaniasis Caused by *L. braziliensis*: An Imported Case Report]. *Rev Chilena Infectol* **2018**, *35*, 612–616, doi:10.4067/S0716-10182018000500612.

47. Montalvo, A.M.; Fraga, J.; Blanco, O.; González, D.; Monzote, L.; Soong, L.; Capó, V. Imported Leishmaniasis Cases in Cuba (2006-2016): What Have We Learned. *Trop Dis Travel Med Vaccines* **2018**, *4*, 7, doi:10.1186/S40794-018-0067-3.

48. Mateo, M.; Cruz, I.; Flores, M.D.; López-Vélez, R. [Slowly Progressing Skin Ulcers Following a Stay in Costa Rica]. *Enfermedades infecciosas y microbiologia clinica* **2005**, *23*, 243–244, doi:10.1157/13073152.

49. ME, W.; LC, L. Photo Quiz. Healthy Student with a Papule. New World Cutaneous Leishmaniasis. *Clin Infect Dis* **2001**, *33*, 816–816, doi:10.1086/322714.

50. Galioto, P.; Fornaro, V. A Case of Mucocutaneous Leishmaniasis. *Ear Nose Throat J* **2002**, *81*, 46–48.

51. TF, H.; P, G.; WF, S.; DA, H.; R, M.; DJ, H. Photo Quiz. Lesion on the Arm of a Returning Traveler. Cutaneous Leishmaniasis. *Clin Infect Dis* **2001**, *33*, 815–815, doi:10.1086/322713.

52. Ismailjee, S.B.; Bernstein, J.M.; Burdette, S.D. Bite the Hand That Sprays You. *Skinmed* **2006**, *5*, 296–299, doi:10.1111/J.1540-9740.2006.05028.X.

53. Zaghi, D.; Panosian, C.; Gutierrez, M.A.; Gregson, A.; Taylor, E.; Ochoa, M.T. New World Cutaneous Leishmaniasis: Current Challenges in Diagnosis and Parenteral Treatment. *J Am Acad Dermatol* **2011**, *64*, 587–592, doi:10.1016/J.JAAD.2009.08.045.

54. Challener, D.; Abu Saleh, O. A Traveler's Unwanted Souvenir. *Am J Med* **2018**, *131*, e137–e138, doi:10.1016/J.AMJMED.2017.09.033.

55. Mann, S.; Phupitakphol, T.; Davis, B.; Newman, S.; Suarez, J.A.; Henao-Martínez, A.; Franco-Paredes, C. Case Report: Cutaneous Leishmaniasis Due to *Leishmania* (*Viannia*) *panamensis* in Two Travelers Successfully Treated with Miltefosine. *Am J Trop Med Hyg* **2020**, *103*, 1081–1084, doi:10.4269/AJTMH.20-0086.

56. Abadir, A.; Patel, A.; Haider, S. Systemic Therapy of New World Cutaneous Leishmaniasis: A Case Report and Review Article. *Can J Infect Dis Med Microbiol* **2010**, *21*, e79-e83, doi:10.1155/2010/768645.

57. Leitner, V.; Weingast, J.; Harmankaya, K.; Walochnik, J.; Pehamberger, H.; Petzelbauer, P.; Auer, H.; Binder, M. Leishmaniasis in the Tongue of an Immunocompetent Man. *Am J Trop Med Hyg* **2010**, *82*, 597–599, doi:10.4269/AJTMH.2010.09-0698.

58. Poeppl, W.; Walochnik, J.; Pustelnik, T.; Auer, H.; Mooseder, G. Cutaneous Leishmaniasis after Travel to Cyprus and Successful Treatment with Miltefosine. *Am J Trop Med Hyg* **2011**, *84*, 562–565, doi:10.4269/AJTMH.2011.10-0645.

59. Ondriska, F.; Bukovinova, P.; Votypka, J.; Nohynkova, E.; Boldis, V. Imported New World Cutaneous Leishmaniasis in a Traveller from Slovakia. *Bratisl Lek Listy* **2015**, *116*, 203–206, doi:10.4149/BLL_2015_040.

60. Zanger, P.; Kötter, I.; Raible, A.; Gelanew, T.; Schönian, G.; Kremsner, P.G. Case Report: Successful Treatment of Cutaneous Leishmaniasis Caused by *Leishmania aethiopica* with Liposomal Amphothericin B in an Immunocompromised Traveler Returning from Eritrea. *Am J Trop Med Hyg* **2011**, *84*, 692–694, doi:10.4269/AJTMH.2011.10-0712.

61. Gilkey, T.; Ulman, C.A.; McGwire, B.; Plaza, J.A. Cutaneous *Leishmania aethiopica* Diagnosed in the United States. *Dermatol Online J* **2021**, *27*, doi:10.5070/D327854705.

62. Vanbrabant, P.; van den Broucke, S.; Soentjens, P. Cutaneous Ulcer after a Stay in the Tropics. *Neth J Med* **2015**, *73*, 44.

63. Goodrich, E.S.; Sears, S.C.; Sorrells, T.; Radike, J.K.; Miladi, A.; Glass, J.S. A Case of Cutaneous Leishmaniasis *guyanensis* Mimicking Otitis Externa. *Mil Med* **2017**, *182*, e1969–e1972, doi:10.7205/MILMED-D-17-00054.

64. Berens-Riha, N.; Fleischmann, E.; Pratlong, F.; Bretzel, G.; von Sonnenburg, F.; Löscher, T. Cutaneous Leishmaniasis (*Leishmania tropica*) in a German Tourist after Travel to Greece. *J Travel Med* **2009**, *16*, 220–222, doi:10.1111/J.1708-8305.2008.00291.X.

65. Bormann, G.; William, T.; Schulz, A.; Marsch, W.; Gaber, G. [American Cutaneous Leishmaniasis: Special Features in Diagnosis and Therapy]. *Dtsch Med Wochenschr* **2003**, *128*, 2065–2068, doi:10.1055/S-2003-42703.

66. Blaylock, J.M.; Wortmann, G.W. A Case Report and Literature Review of "Chiclero's Ulcer." *Travel Med Infect Dis* **2012**, *10*, 275–278, doi:10.1016/J.TMAID.2012.08.005.

67. Guery, R.; Henry, B.; Martin-Blondel, G.; Rouzaud, C.; Cordoliani, F.; Harms, G.; Gangneux, J.P.; Foulet, F.; Bourrat, E.; Baccard, M.; et al. Liposomal Amphotericin B in Travelers with Cutaneous and Muco-Cutaneous Leishmaniasis: Not a Panacea. *PLoS Negl Trop Dis* **2017**, *11*, doi:10.1371/JOURNAL.PNTD.0006094.

68. Update: Cutaneous Leishmaniasis in U.S. Military Personnel --- Southwest/Central Asia, 2002--2004 Available online: https://www.cdc.gov/mmwr/preview/mmwrhtml/mm5312a4.htm (accessed on 13 July 2022).

69. Taxy, J.B.; Goldin, H.M.; Dickie, S.; Cibull, T. Cutaneous Leishmaniasis: Contribution of Routine Histopathology in Unexpected Encounters. *Am J Surg Pathol* **2019**, *43*, 195–200, doi:10.1097/PAS.0000000000001177.

70. Mongkolrattanothai, K.; Nadipuram, S.M.; Krakowski, A.C.; Stone, M.M.; Krogstad, P.; Lehman, D. Leishmaniasis Gone Viral: Social Media and an Outbreak of Cutaneous Leishmaniasis. *Pediatr Dermatol* **2016**, *33*, e276–e277, doi:10.1111/PDE.12910.

71. Basnett, A.; Nguyen, T.A.; Cannavino, C.; Krakowski, A.C. Ablative Fractional Laser Resurfacing with Topical Paromomycin as Adjunctive Treatment for a Recalcitrant Cutaneous Leishmaniasis Wound. *Lasers Surg Med* **2015**, *47*, 788–791, doi:10.1002/LSM.22426.

72. Kuilder, J.S.; Wismans, P.J.; Baerveldt, E.M.; van Hellemond, J.J.; de Mendonça Melo, M.; van Genderen, P.J.J. Leishmania Major Cutaneous Leishmaniasis in 3 Travelers Returning from Israel to the Netherlands. *Emerg Infect Dis* **2016**, *22*, 2022–2024, doi:10.3201/EID2211.161154.

73. Newlove, T.; Robinson, M.; Meehan, S.A.; Pomerantz, R. Old World Cutaneous Leishmaniasis. *Dermatol Online J* **2012**, *18*, 32, doi:10.18295/squmj.9.2021.133.

74. Kelly, P.; Baudry, T.; Peyron, F. Imported Cutaneous Leishmaniasis in a Short-Term Traveler Returning from Central Mali - The Role of PCR. *Travel Med Infect Dis* **2012**, *10*, 97–100, doi:10.1016/J.TMAID.2012.01.004.

75. Spinicci, M.; Zammarchi, L.; Gramiccia, M.; di Muccio, T.; Bartolozzi, D.; Corsi, P.; Trotta, M.; Bartoloni, A. Effective Meglumine Antimoniate Intralesional Therapy for Chiclero's Ulcer Refractory to Systemic Liposomal Amphotericin B. *J Travel Med* **2021**, *28*, 1–2, doi:10.1093/JTM/TAAA169.

76. Eldin, C.; L'Ollivier, C.; Ranque, S.; Gautret, P.; Parola, P. "Chiclero's Ulcer" Due to *Leishmania mexicana* in Travelers Returning from Central America: A Case Report and Review of the Literature. *Pathogens* **2021**, *10*, doi:10.3390/PATHOGENS10091112.

77. Tsai, P.H.; Chen, Y.T.; Liau, J.Y.; Huang, M.H.; Hsu, H.M.; Yeong, E.K.; Hung, C.C. Molecular Diagnosis and Therapy for Cutaneous Leishmaniasis of a Returned Traveler from Mexico. *J Microbiol Immunol Infect* **2021**, *54*, 1154–1158, doi:10.1016/J.JMII.2020.07.007.

78. Cohen, J.; Saavedra, A.; Sax, P.; Lipworth, A. Pink Plaque on the Arm of a Man after a Trip to Mexico: Cutaneous Leishmaniasis. *Dermatol Online J* **2015**, *21*, 13030.

79. van Hees, C.; van Hellemond, J.; den Boer, M. [A Boy with an Unexpected Souvenir from Morocco]. *Nederlands Tijdschrift voor Geneeskunde* **2014**, *158*, A7606.

80. Nasri, J.; Cajacob, L.; Wirz, E.; Ruf, M.T.; Blum, J.; Mühleisen, B.; Navarini, A.A.; Maul, L. v. Pre-Ulcerative Leishmaniasis Mimicking Chilblains in a Returning Traveller from Southern Europe. *J Eur Acad Dermatol Venereol* **2021**, *35*, e503–e505, doi:10.1111/JDV.17233.

81. Knöpfel, N.; Noguera-Morel, L.; Azorin, D.; Sanz, F.; Torrelo, A.; Hernández-Martín, A. Cutaneous *Leishmania tropica* in Children: Report of Three Imported Cases Successfully Treated with Liposomal Amphotericin B. *J Eur Acad Dermatol Venereol* **2018**, *32*, e8–e10, doi:10.1111/JDV.14434.

82. EM, T.; JR, M.; A, V. Photo Quiz: A 24-Year-Old Traveler with an Insect Bite and Rash. *Clin Infect Dis* **2015**, *61*, 1314, doi:10.1093/CID/CIV359.

83. Kitano, H.; Sanjoba, C.; Goto, Y.; Iwamoto, K.; Kitagawa, H.; Nomura, T.; Omori, K.; Shigemoto, N.; Hide, M.; Matsumoto, Y.; et al. Complicated Cutaneous Leishmaniasis Caused by an Imported Case of *Leishmania tropica* in Japan: A Case Report. *Trop Med Health* **2021**, *49*, doi:10.1186/S41182-021-00312-4.

84. Barry, M.A.; Koshelev, M. v.; Sun, G.S.; Grekin, S.J.; Stager, C.E.; Diwan, A.H.; Wasko, C.A.; Murray, K.O.; Woc-Colburn, L. Cutaneous Leishmaniasis in Cuban Immigrants to Texas Who Traveled through the Darién Jungle, Panama. *Am J Trop Med Hyg* **2014**, *91*, 345–347, doi:10.4269/AJTMH.14-0124.

85. Crowe, A.; Slavin, J.; Stark, D.; Aboltins, C. A Case of Imported *Leishmania infantum* Cutaneous Leishmaniasis; an Unusual Presentation Occurring 19 Years after Travel. *BMC Infect Dis* **2014**, *14*, 597, doi:10.1186/S12879-014-0597-X.

86. Eichner, S.; Thoma-Uszynski, S.; Herrgott, I.; Sebald, H.; Debus, A.; Tsianakas, A.; Ehrchen, J.; Harms, G.; Simon, M.; Sunderkötter, C.; et al. Clinical Complexity of *Leishmania* (*Viannia*) *braziliensis* Infections amongst Travelers. *Eur J Dermatol* **2013**, *23*, 218–223, doi:10.1684/EJD.2013.1934.

87. Schönian, G.; Nasereddin, A.; Dinse, N.; Schweynoch, C.; Schallig, H.D.F.H.; Presber, W.; Jaffe, C.L. PCR Diagnosis and Characterization of *Leishmania* in Local and Imported Clinical Samples. *Diagn Microbiol Infect Dis* **2003**, *47*, 349–358, doi:10.1016/S0732-8893(03)00093-2.

88. Lange, C.G.; Salata, R.A. [Cutaneous Leishmaniasis]. *Med Klin (Munich)* **2001**, *96*, 561–562, doi:10.1007/PL00002242.

89. Brecelj, M.; Pikelj, F.; Gubenšek, F.; Anderluh, G. Polymerase Chain Reaction as a Diagnostic Tool for Detecting *Leishmania*. *Infection* **2000**, *28*, 111–113, doi:10.1007/S150100050057.

90. Crone, C.G.; Helleberg, M. Cutaneous Leishmaniasis with Secondary Mucosal Disease in a Traveller Due to *Leishmania* (*Viannia*) *braziliensis*. *J Travel Med* **2020**, *27*, doi:10.1093/JTM/TAZ093.

91. Clegg, A.; Hall, C.; Lockwood, D. An Ulcer That Will Not Heal. *BMJ* **2018**, *362*, doi:10.1136/BMJ.K3042.

92. Raghunath, R.S.; Yong, A.S.W.; Igali, L.; Tan, E.; Lockwood, D. Cutaneous Leishmaniasis in a Returning UK Traveller. *Postgrad Med J* **2014**, *90*, 540–541, doi:10.1136/POSTGRADMEDJ-2014-132620.

93. Michelerio, A.; Barruscotti, S.; Bossi, G.; Brazzelli, V. Pediatric Old World Cutaneous Leishmaniasis Treated with Oral Fluconazole: A Case Series. *Pediatr Dermatol* **2018**, *35*, 384–387, doi:10.1111/PDE.13493.

94. Larréché, S.; Launay, G.; Weibel Galluzzo, C.; Bousquet, A.; Eperon, G.; Pilo, J.E.; Ravel, C.; Chappuis, F.; Dupin, M.; Mérens, A. Cluster of Zoonotic Cutaneous Leishmaniasis (*Leishmania major*) in European Travelers Returning from Turkmenistan. *J Travel Med* **2013**, *20*, 400–402, doi:10.1111/JTM.12065.

95. Zhang, M.; Liu, F.; Liu, H.B.; Hu, W.X.; Sang, H. Imported Cutaneous Leishmaniasis Caused by *Leishmania major* in a Chinese Laborer Who Worked in Saudi Arabia. *An Bras Dermatol* **2016**, *91*, 365–367, doi:10.1590/ABD1806-4841.20163820.

96. Khan, M.A.A.; Chowdhury, R.; Nath, R.; Hansen, S.; Nath, P.; Maruf, S.; Abd El Wahed, A.; Mondal, D. Imported Cutaneous Leishmaniasis: Molecular Investigation Unveils *Leishmania major* in Bangladesh. *Parasit Vectors* **2019**, *12*, doi:10.1186/S13071-019-3771-6.

97. Rahman, H.; Razzak, M.; Chanda, B.; Bhaskar, K.; Mondal, D. Cutaneous Leishmaniasis in an Immigrant Saudi Worker: A Case Report. *Journal of Health, Population and Nutrition* **2014**, *32*, 372–376.

98. Schwartz, T.; Jensenius, M.; Blomberg, B.; Fladeby, C.; Mæland, A.; Pettersen, F.O. Imported Visceral Leishmaniasis and Immunosuppression in Seven Norwegian Patients. *Trop Dis Travel Med Vaccines* **2019**, *5*, 16, doi:10.1186/S40794-019-0092-X.

99. Suqati, A.A.; Pudszuhn, A.; Hofmann, V.M. Mucocutaneous Leishmaniasis: Case Report and Literature Review of a Rare Endonasal Infection. *Pan Afr Med J* **2020**, *36*, 1–4, doi:10.11604/PAMJ.2020.36.292.24543.

100. Menten, K.; Soentjens, P.; Caenepeel, P.; Lemkens, P. Mucocutaneous Leishmaniasis of the Nose: A Case Report. *B-ENT* **2015**, *11*, 77–80.

101. Ehlert, N.; Seilmaier, M.; Guggemos, W.; Löscher, T.; Meurer, A.; Wendtner, C.M. [Severe Oral Mucositis in a Patient with HIV Infection]. *Dtsch Med Wochenschr* **2013**, *138*, 1601–1605, doi:10.1055/S-0033-1343296.

102. Terhorst, D.; Blume-Peytavi, U.; Schönian, G.; Schewe, C.; Haas, N.; Burbach, G.J. Leishmaniasis: A Reminder in the Face of Forgotten Travel. *J Pediatr* **2012**, *161*, 966, doi:10.1016/J.JPEDS.2012.05.013.

103. Darné, S.; Sinclair, S.A. A Sandfly in Surrey? A Case of Cutaneous Leishmaniasis in the United Kingdom without History of Recent Travel to an Endemic Area. *Clin Exp Dermatol* **2006**, *31*, 155–156, doi:10.1111/J.1365-2230.2005.01984.X.

104. Siah, T.; Lavender, T.; Charlton, F.; Wahie, S.; Schwab, U. An Unusual Erysipelas-like Presentation. *Dermatol Online Journal* **2014**, *20*, 21255.

105. Ito, K.; Takahara, M.; Ito, M.; Oshiro, M.; Takahashi, K.; Uezato, H.; Imafuku, S. An Imported Case of Cutaneous Leishmaniasis Caused by *Leishmania* (*Leishmania*) *donovani* in Japan. *J Dermatol* **2014**, *41*, 926–928, doi:10.1111/1346-8138.12609.

106. van der Snoek, E.M.; Lammers, A.M.; Kortbeek, L.M.; Roelfsema, J.H.; Bart, A.; Jaspers, C.A.J.J. Spontaneous Cure of American Cutaneous Leishmaniasis Due to *Leishmania naiffi* in Two Dutch Infantry Soldiers. *Clin Exp Dermatol* **2009**, *34*, doi:10.1111/J.1365-2230.2009.03658.X.

107. Saroufim, M.; Charafeddine, K.; Issa, G.; Khalifeh, H.; Habib, R.H.; Berry, A.; Ghosn, N.; Rady, A.; Khalifeh, I. Ongoing Epidemic of Cutaneous Leishmaniasis among Syrian Refugees, Lebanon. *Emerg Infect Dis* **2014**, *20*, 1712–1715, doi:10.3201/EID2010.140288.

108. Hijawi, K.J.F.; Hijjawi, N.S.; Ibbini, J.H. Detection, Genotyping, and Phylogenetic Analysis of *Leishmania* Isolates Collected from Infected Jordanian Residents and Syrian Refugees Who Suffered from Cutaneous Leishmaniasis. *Parasitol Res* **2019**, *118*, 793–805, doi:10.1007/S00436-019-06222-Z.

109. Talas, J.; Mielcarek, K.; Wu, J.; Brunner, M.; Steinhoff, M.; Zouboulis, C.C. [Cutaneous Leishmaniasis in Germany-Still a Travel-Related Disease]. *Der Hautarzt; Zeitschrift fur Dermatologie, Venerologie, und verwandte Gebiete* **2022**, *73*, 146–151, doi:10.1007/S00105-021-04890-6.

110. van Kesteren, L.; Maniewski, U.; Bottieau, E.; Cnops, L.; Huits, R. Cutaneous Leishmaniasis in Syrian Refugee Children: A Case Series. *Pediatr Infect Dis J* **2020**, *39*, E154–E156, doi:10.1097/INF.0000000000002676.

111. Imai, K.; Tarumoto, N.; Amo, K.; Takahashi, M.; Sakamoto, N.; Kosaka, A.; Kato, Y.; Mikita, K.; Sakai, J.; Murakami, T.; et al. Non-Invasive Diagnosis of Cutaneous Leishmaniasis by the Direct Boil Loop-Mediated Isothermal Amplification Method and MinION™ Nanopore Sequencing. *Parasitol Int* **2018**, *67*, 34–37, doi:10.1016/J.PARINT.2017.03.001.

112. Bradshaw, S.; Litvinov, I. v. Dermal Leishmaniasis in a 25-Year-Old Syrian Refugee. *CMAJ* **2017**, *189*, E1397, doi:10.1503/CMAJ.170844.

113. Downing, C.P.; Woc-Colburn, L.; Tyring, S.K. Nasal Erythema and Crusting after a Trip to the Venezuelan Rainforest. *JAMA* **2014**, *312*, 1250–1251, doi:10.1001/JAMA.2014.3610.
