# Supplementary figures and images for: Non-Endemic Leishmaniases Reported Globally in Humans between 2000 and 2021—A Comprehensive Review
Source: Pathogens. 2022 Aug 16;11(8):921. doi: 10.3390/pathogens11080921 (PMC9415673; doi:10.3390/pathogens11080921)

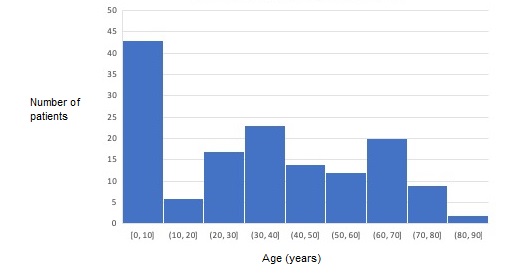

Supplement: Supplementary file 1 [file pathogens-11-00921-s001.zip › Supplementary Figure S1.jpg]

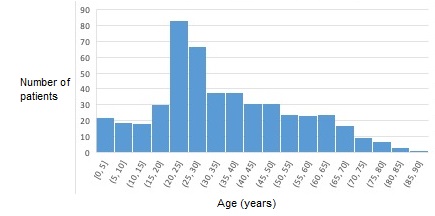

Supplement: Supplementary file 1 [file pathogens-11-00921-s001.zip › Supplementary Figure S2.jpg]
